# Forecasting mangrove ecosystem degradation utilizing quantifiable eco-physiological resilience -A study from Indian Sundarbans

**DOI:** 10.1038/s41598-020-63586-4

**Published:** 2020-04-21

**Authors:** Mst Momtaj Begam, Rajojit Chowdhury, Tapan Sutradhar, Chandan Mukherjee, Kiranmoy Chatterjee, Sandip Kumar Basak, Krishna Ray

**Affiliations:** 1 Environmental Biotechnology Group, Department of Botany, West Bengal State University, Berunanpukuria, Malikapur, Barasat Kolkata, 700126 India; 2 Sarat Centenary College, Dhaniakhali, Hooghly, 712302 West Bengal India; 3 Department of Statistics, Bidhannagar College, Salt Lake City, Sector 1, Block EB, Kolkata, 700064 India

**Keywords:** Ecology, Ecology, Environmental sciences

## Abstract

Sundarbans mangrove forest, the world’s largest continuous mangrove forests expanding across India and Bangladesh, in recent times, is immensely threatened by degradation stress due to natural stressors and anthropogenic disturbances. The degradation across the 19 mangrove forests in Indian Sundarbans was evaluated by eight environmental criteria typical to mangrove ecosystem. In an attempt to find competent predictors for mangrove ecosystem degradation, key eco-physiological resilience trait complex specific for mangroves from 4922 individuals for physiological analyses with gene expression and 603 individuals for leaf tissue distributions from 16 mangroves and 15 associate species was assessed along the degradation gradient. The degradation data was apparently categorized into four and CDFA discriminates 97% of the eco-physiological resilience data into corresponding four groups. Predictive Bayesian regression models and mixed effects models indicate osmolyte accumulation and thickness of water storage tissue as primary predictors of each of the degradation criteria that appraise the degradation status of mangrove ecosystem. RDA analyses well represented response variables of degradation explained by explanatory resilience variables. We hypothesize that with the help of our predictive models the policy makers could trace even the cryptic process of mangrove degradation and save the respective forests in time by proposing appropriate action plans.

## Introduction

The Sundarbans stretches along the coast of Bangladesh and India and forms the largest contiguous mangrove forest in the world.The Indian part of Sundarbans received its formal designation recently in 2019 as Ramsar site (https://rsis.ramsar.org/ris/2370) and the Government of Bangladesh had already designated their part of the mangrove forests as Ramsar in 1992 (https://rsis.ramsar.org/ris/560) thus bringing the entire mangrove swamp under the domain of Ramsar wise use framework. UNESCO announced the Sundarbans a World Heritage Site in 1997^1^. In India, the stretch of Sundarbans is extended in southern part of the state of West Bengal along the estuarine coastline. It is the abode of highly diverse true mangrove species and some typical back mangroves referred as mangrove associates that do not possess the true mangrove characters but have the adequate potential to adapt to the mangrove environment. A heterogeneous assemblage of representatives from divergent and unrelated families migrating from mesophytic environment towards this estuarine extremophilic ecosystem and climaxing in a convergent evolution bring in uniqueness in this mangrove niche.Mangroves are among coastal foundation species that structure the coastal floral and faunal communities by modifying their habitats leaving a major influence on surrounding ecosystem structure and function^2^. Hence mangrove degradation is thought to impact the coastal ecosystem greatly. At the present moment small mangrove patches in Indian Sundarbans are facing immense threats of degradation^3^. This rapid degradation is caused due to increase in anthropogenic interferences such as conversion for urbanization, pisciculture, agriculture, salt farming, tourism, mining, refineries, dam and road constructions; changes in hydrological regimes; coastal pollution; siltation; exploitation of fishery resources; cattle grazing; incessant deforestation^4^. Natural stressors like increase in sediment salinity, increasing anaerobic conditions due to sea level rise, increased level of sulfide in estuarine sediments, continuous erosion by high tidal forces and cyclonic storms also equally aggravate the degradation process of this mangrove ecosystem^3^. The most visible consequence of degradation is the poor stunted growth forms of mangrove stands with obvious decline in density and forest coverage^3^. Nutrient limitation, salinity rise, anoxicity increase and sulfide build-up, negatively controlled forest structure causing declines of forest coverage from ~98% to ~11% in Indian Sundarbans^3^. Mangrove species usually develop natural eco-physiological resilience against the degraded environments following their acclimation capabilities for maintaining homeostasis. In highest level of degradation, the mangrove ecosystem homeostasis collapses to such an extent that it can no longer continue the normal processes of secondary succession and slowly advances towards the verge of extinction. For example, as a consequence of salinity rise in mangrove forests of Indian Sundarbans, alteration in species distribution occurred causing disappearance of salt sensitive *Heritiera fomes*, *Xylocarpus* spp., and *Phoenix paludosa *from many forests and concomitant expansion of *Excoecaria agallocha* and *Avicennia* spp. occurred largely into degraded forests due to their high adaptive capability across the Indian Sundarbans^3^.

Next to direct degradation stressors (natural or anthropogenic) is cryptic ecological degradation processes. Researchers show, changes in Sri Lankan inland freshwater management cryptically affect the coastal zone by introducing an excess of fresh water^5^. The resilience from mangroves against this cryptic process culminates in adverse shifts in the composition of mangrove species, dominance of fresh water loving mangrove associate *Acrostichum aureum* L. at the expense of typical, functional, valuable true mangrove species but without loss of spatial extent^5^. The authors emphasize that such cryptic ecological degradation must be acknowledged by policy and decision makers if mangrove protection is an aim. The research team argues that early detection of such cryptic processes should be adopted and is essential for the prevention of further mangrove degradation^5^. The cryptic operation of ecosystem stressors is not always comprehensible to the scientists at its preliminary state, but the plant physiological system is so sensitive that it can easily apprehend the perturbations in ecosystem environment at its onset and strives in response to develop natural resilience to ascertain their survival. Our research involves exploitation of this natural eco-physiological resilience of mangroves in response to onset of degradation process to predict the degradation factors and identify the stage of degradation.

**Figure 1:**
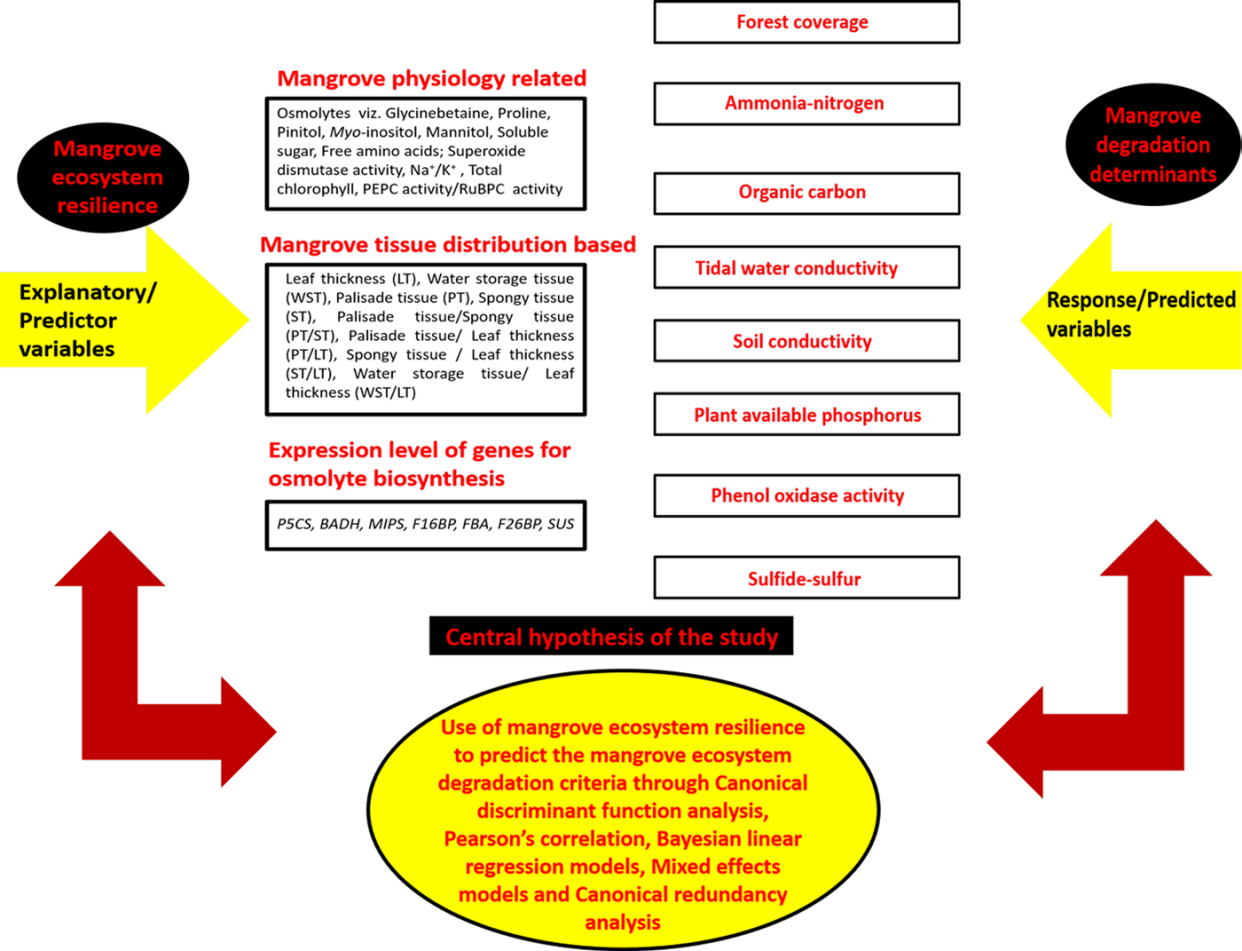
The integral design of the study relating the predictor (explanatory) and predicted (response) variables to the central hypothesis. All the predictor variables fit in a typical mangrove eco-physiological resilience trait complex whereas the response variables are mainly the degradation criteria fundamental to mangrove ecosystem degradation.

The concept of ecological resilience or ecosystem resilience^6,7^ reflects the ability of a system to absorb disturbance and reorganize while undergoing change and yet retain the same controls on function and structure. As resilience declines, the synergistic effects of negative pressures can make ecosystems more vulnerable to changes with sudden shift from desired to less desired states^8^. Loss of resilience is not always a perceptible gradual degradation;sometimes this loss can result in a sudden shift triggered by a stochastic event or a threshold is suddenly achieved collapsing the resilience^9–12^. Until the resilience threshold is exceeded by the disturbance, the ecosystem may not give any indication of vulnerability. Our research addresses this dynamic interplay between disturbance and resilience. We hypothesize that in response to certain degradation criteria relating to mangrove ecosystem, mangrove species develop natural eco-physiological resilience. We have selected some of the quantifiable components of this eco-physiological resilience and measured them across a degradation gradient. We used these measured resilience components to predict the degradation criteria of mangrove forests (Fig. 1). As long as ecosystem resilience persists, the major undesirable shift in ecosystem is not observable. Our objective is to attract the attention of the ecosystem managers for saving mangroves from degradation by predicting the degradation parameters based on the trend of eco-physiological resilience of mangrove species.

This study was conducted across 19 small mangrove forests of Indian Sundarbans presently at various stages of degradation^3^. These forests included many pristine undisturbed mangrove habitats as controls as well as other mangrove forests under different facets of degradation. We measured the degradation gradient on the basis of eight quantifiable mangrove ecosystem evaluators viz. soil ammonia nitrogen (NH_4_-N), organic carbon, plant available phosphorus (P), soil and tidal water conductivity, soil phenol oxidase activity, sulfide content and forest cover percentage (Fig. 1).

Across this degradation gradient mangrove eco-physiology was expected to be variably affected.

We considered the organismal responses of acclimation as eco-physiological resilience and quantify these acclimatory capacities across the degradation gradient. Acclimation is defined as adjustment of physiological and phenotypic conditions that accrues a net benefit^13^. The individuals and species may differ in their acclimation maxima and acclimation capacities in populations of native organisms are to be evaluated in complexes of traits, not in individual traits, as effective strategies for coping with the environment^13^. We primarily evaluated the degree of acclimation (as a complex of typical mangrove eco-physiological attributes) such as total Na^+^/K^+^ ratio, levels of accumulated osmolytes (proline, free amino acids, soluble sugars, sugar alcohols like mannitol, inositol, pinitol and quaternary ammonium compounds, QACs such as glycinebetaine), activity ratio of PEP carboxylase (PEPC) and RuBP carboxylase (RuBPC), total chlorophyll concentration, superoxide dismutase (SOD) activity, leaf thickness (LT), palisade tissue thickness (PT), spongy tissue thickness (ST), water storage tissue thickness (WST), palisade–spongy tissue thickness ratio (PT/ST), palisade tissue–leaf thickness ratio (PT/LT), spongy tissue–leaf thickness ratio (ST/LT) and water storage tissue-leaf thickness ratio (WST/LT) from different species of mangroves and associates (Fig. 1). Gene expression levels were also tested for seven genes (*P5CS* for proline synthesis, *BADH* for glycinebetaine synthesis, *MIPS* for myo-inositol synthesis, *SUS*, *F1, 6BP*, *F*2, *6BP*, *FBA* for soluble sugar synthesis) associated with biosynthetic pathways of osmolytes across a degradation gradient. These quantifiable traits were considered as measurable eco-physiological resilience shown by the mangrove and associate species in response to degradation gradient across different forests of Indian Sundarbans. These resilience mechanisms assist the species to survive under degraded condition. We hypothesize that the pattern of resilience might provide clues to predict the degradation factors (Fig. 1) concerning a particular forest and the prediction outcomes bear great potential in adopting proper precautionary measures in advance to check the degradation of a mangrove forest.

## Results

### Categorization of degradation phases and corresponding resilience

**Figure 2:**
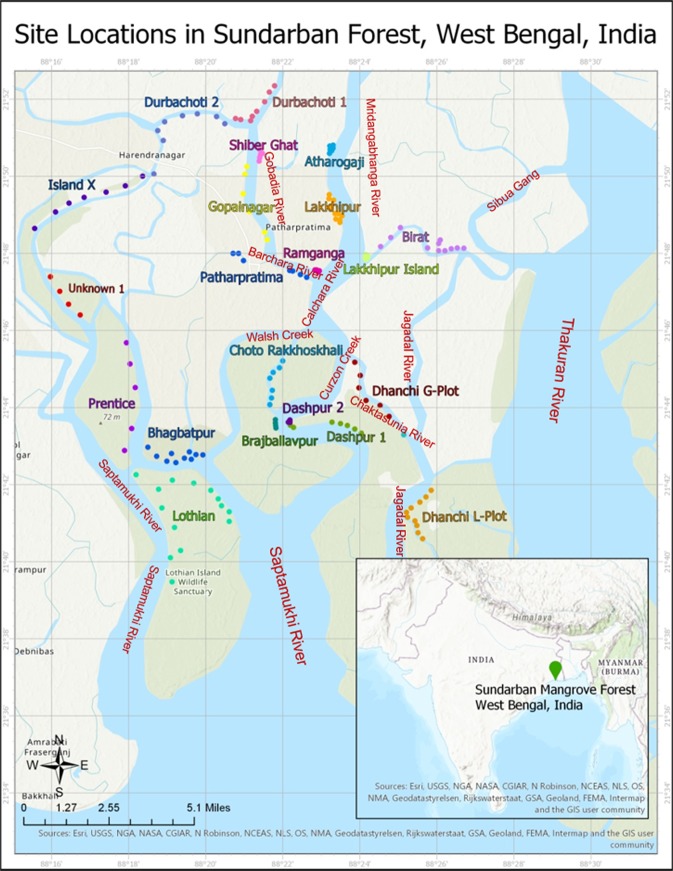
Locations of 19 small mangrove forests in Indian Sundarbans. Sites of sediment and leaf sample collection are shown for each forest in different colours. All the forests are located on the shoreline of different rivers. Major rivers are named in the map^67^. Light green coloured islands are protected areas under Sundarban Biosphere Reserve (India). The map was created using ArcGIS Pro 2.4 (https://www.esri.com/en-us/arcgis/products/arcgis-pro/overview) where the base map feature was used to create the layout of the map and the location data collected in the study were translated into x, y coordinates on the map.

The ecosystem degradation variables of soil ammonia-nitrogen, organic carbon, plant available inorganic phosphorus, conductivity of soil and tidal water, soil phenol oxidase activity, sulfide and forest coverage across 19 mangrove forests (Fig. 2) were evaluated. Depending on the recorded data (for sediment variables with little variation across the seasons except for tidal water)^3^ the categorization of the degraded state of the forests was carried out superficially to postulate four degradation stages such as pristine (control), intermediate degradation 1, intermediate degradation 2 and maximal degradation (Table S1). From pristine (control) towards maximally degraded forests a steady decline in nutrient variables and forest % covers along with rise in salinity and sulfide was clearly viewed (Tables S1, S6A). Corresponding data on eco-physiological resilience were developed across these four degradation categories (Table S6B). We performed a canonical discriminant function analysis (CDFA) (Fig. S1) to substantiate the validity of linear combination of the eco-physiological resilience data with the four different degradation stages. Probable overlapping of recorded values among the four degradation classes was checked and finally eco-physiological resilience data was correctly discriminated in to four degradation stages through CDFA. In our study the output data set for CDFA shows significance (p = 0.000) with a smaller value of 0.009 for Wilks’ lambda indicating a greater discriminatory ability for the functions (Table S2). For eco-physiological resilience, 97.0% of originally grouped data are correctly classified (Fig. S1). These results strongly reflect that our data categorization is statistically appropriate and mangrove ecosystem degradation gradient can strongly discriminate the corresponding data of eco-physiological resilience into clear four degradation heads.

### Pearson’s correlation analyses

Pearson’s correlation analyses between all the degradation factors and eco-physiological resilience data (Table 1) reveal that all the osmolytes except pinitol did demonstrate a very strong linear correlation to each of the attributes of degradation. Pinitol showed a moderate correlation. Osmolytes, having strong positive correlation with the tidal water and soil conductivity and sulfides, showed strong negative correlation with other environmental factors. For the earlier mentioned three factors, the osmolytes having strong negative correlation showed strong positive correlation with rest of the degradation factors. The other acclimation responses including total Na^+^/K^+^ ratio, activity ratio of PEPC and RuBPC, total chlorophyll concentration and SOD activity demonstrated very low correlation with all the degradation components. Thus these analyses proved osmotic acclimation in mangroves to be the key eco-physiological resilience response across the degradation gradient. Not only that, leaf thickness (LT), water storage tissue (WST) and WST/LT exhibited very strong negative correlation with all the criteria of degradation except for the tidal water and soil conductivity and sulfides; with these three factors LT, WST and WST/LT displayed strong positive correlation. On the other hand PT/LT and ST/LT showed exactly the reverse orientation maintaining high correlation with all the degradation factors. In mangrove leaves WST is actually responsible for maintaining succulence and is prime determinant of LT of the leaves. This succulence under hypersaline environment is a distinguished eco-physiological resilience trait. All the correlation analyses established WST to be primarily directly linked to the degradation factors with LT, PT/LT, ST/LT and WST/LT closely following behind in an indirect mode to be correlated to the degradation gradient. For palisade tissue (PT), spongy tissue (ST) and PT/ST, an overall low correlation with degradation components was observed. The trend of correlation of gene expression with degradation criteria exactly matched with the respective osmolytes for biosynthesis of which these genes are responsible. *P5CS*, *MIPS*, *SUS*, *F16BP*, *F*2*6BP* and *FBA* showed strong positive correlation with tidal water and soil conductivity and sulfides but strong negative correlation with all other factors while *BADH* showed exactly the opposite pattern of strong correlation.

**Table 1:** Pearson’s correlation coefficient analysis between degradation criteria (response variables) and eco-physiological resilience (explanatory variables) *p < 0.05, **p < 0.01, ***p < 0.001.

Response variables Explanatory variables	Forest Coverage	Ammonia-nitrogen	Organic carbon	Tidal water conductivity	Soil conductivity	Plant available phosphorus	Phenol oxidase activity	Sulfide-sulfur
Glycinebetaine	0.851***	0.798***	0.653***	−0.653***	−0.410***	0.739***	0.764***	−0.720***
Proline	−0.794***	−0.736***	−0.617***	0.683***	0.732***	−0.721***	−0.701***	0.806***
Pinitol	0.455***	0.419***	0.268**	−0.345***	−0.335***	0.367***	0.429***	−0.417***
*Myo*-inositol	−0.813***	−0.776***	−0.722***	0.691***	0.599***	−0.719***	−0.738***	0.729***
Soluble sugar	−0.886***	−0.792***	−0.695***	0.637***	0.597***	−0.779***	−0.792***	0.757***
Free amino acids	−0.746***	−0.698***	−0.605***	0.462***	0.608***	−0.663***	−0.657***	0.771***
Mannitol	0.792***	0.746***	0.634***	−0.568***	−0.319***	0.714***	0.663***	−0.659***
Superoxide dismutase	−0.258**	−0.179*	−0.131	0.344***	0.266**	−0.234**	−0.238**	0.238**
Na^+^/K^+^	−0.206*	−0.238**	−0.159	0.15	0.228**	−0.111	−0.212*	0.186*
Total chlorophyll	0.202*	0.235**	0.220*	−0.07	−0.167	0.165	0.204*	−0.185*
PEPC activity/RuBPC activity	0.106	0.175*	0.169	−0.12	−0.123	0.163	0.123	−0.028
Leaf thickness (LT)	−0.859***	−0.808***	−0.746***	0.702***	0.593***	−0.768***	−0.770***	0.770***
Water storage tissue (WST)	−0.855***	−0.806***	−0.738***	0.724***	0.629***	−0.758***	−0.765***	0.799***
Palisade tissue (PT)	−0.417***	−0.462***	−0.385***	0.266**	0.164	−0.408***	−0.348***	0.336***
Spongy tissue (ST)	0.350***	0.442***	0.299***	−0.390***	−0.369***	0.313***	0.272**	−0.505***
Palisade tissue/Spongy tissue (PT/ST)	−0.348***	−0.333***	−0.240**	0.241**	0.283**	−0.202*	−0.241**	0.349***
Palisade tissue/Leaf thickness (PT/LT)	0.756***	0.712***	0.628***	−0.642***	−0.443***	0.683***	0.743***	−0.625***
Spongy tissue /Leaf thickness (ST/LT)	0.735***	0.801***	0.666***	−0.657***	−0.439***	0.713***	0.658***	−0.667***
Water storage tissue/Leaf thickness (WST/LT)	−0.844***	−0.858***	−0.736***	0.736***	0.498***	−0.789***	−0.789***	0.734***
*P5CS*	−0.918***	−0.867***	−0.759***	0.803***	0.698***	−0.805***	−0.826***	0.841***
*BADH*	0.939***	0.874***	0.755***	−0.768***	−0.606***	0.815***	0.886***	−0.809***
*MIPS*	−0.909***	−0.867***	−0.744***	0.772***	0.683***	−0.819***	−0.816***	0.871***
*F16BP*	−0.939***	−0.897***	−0.789***	0.743***	0.620***	−0.872***	−0.848***	0.817***
*FBA*	−0.931***	−0.859***	−0.771***	0.786***	0.653***	−0.840***	−0.836***	0.844***
*F26BP*	−0.939***	−0.872***	−0.743***	0.779***	0.719***	−0.806***	−0.843***	0.863***
*SUS*	−0.943***	−0.871***	−0.744***	0.783***	0.683***	−0.817***	−0.845***	0.862***

### Regression models and prediction of degradation determinants

**Figure 3:**
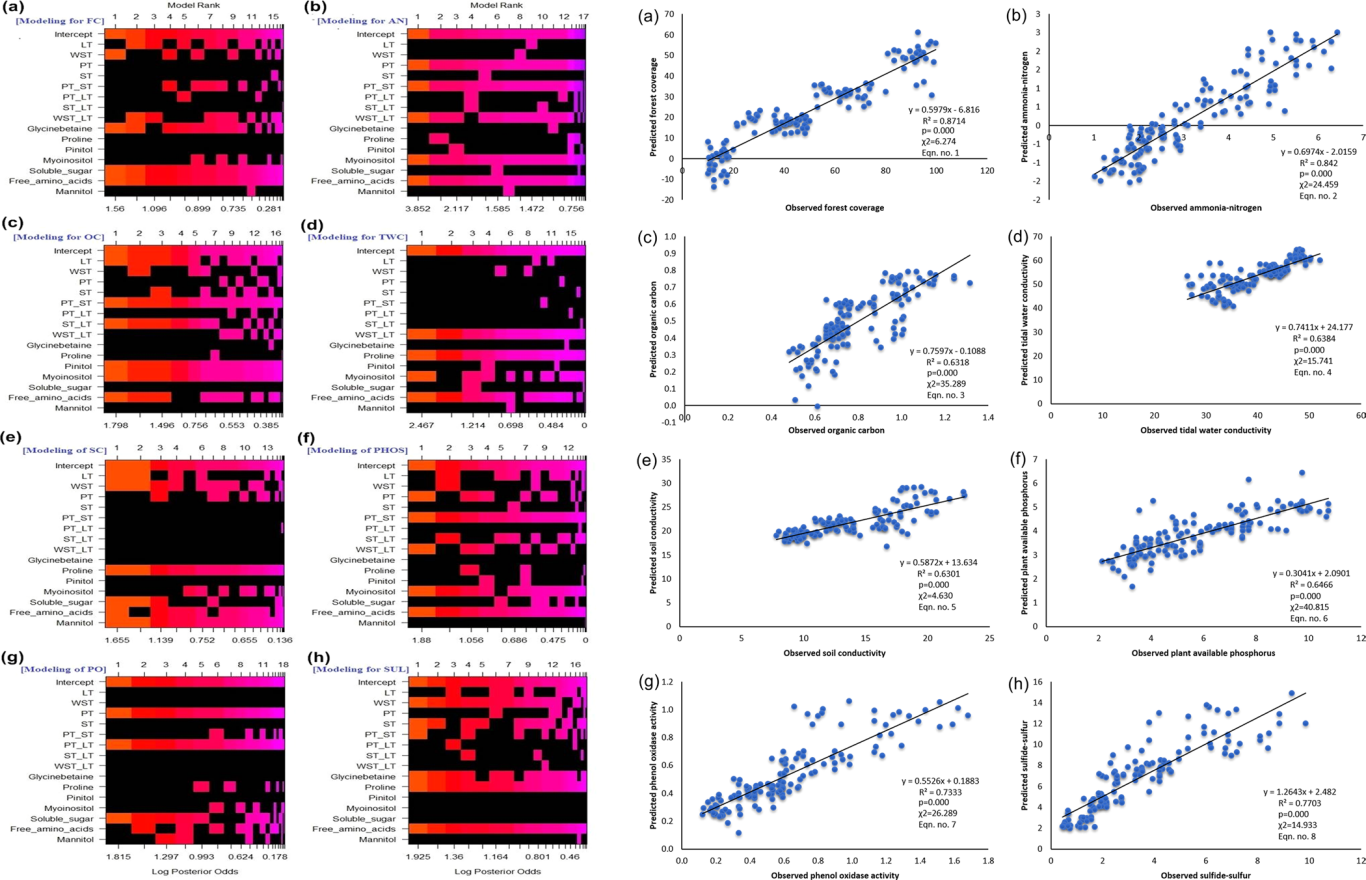
(**a**) Graphical plots representing the model selection strategy in Bayesian Linear Regression analysis based on their ranking in terms of Log Posterior Odds. Columns indexed by 1, 2, 3, …. refer the competitive models arranged in order of their ranks and rows refer different eco-physiological resiliences along with the intercept. In each column (i.e. model), presence (absence) of each of the total 16 predictors (eco-physiological resiliences) was indicated by ‘red’ (‘black’). Response variables in linear relation to predictors: (**a**) FC-forest cover, (**b**) AN-ammonia-nitrogen, (**c**) OC-organic carbon, (**d**) TWC-tidal water conductivity, (**e**) SC-soil conductivity, (**f**) PHOS-phosphorus, (**g**) PO-phenol oxidase, (**h**) SUL-sulfide-sulfur. (**b**) Graphical plots representing the Observed versus Predicted data on different degradation criteria. Predicted data for these eight plots are based on the linear regression equations derived in Table 2A: (**a**) Equation no. 1 for forest coverage (**b**) Equation no. 2 for ammonia-N (**c**) Equation no. 3 for organic carbon (**d**) Equation no. 4 for tidal water conductivity (**e**) Equation no. 5 for soil conductivity (**f**) Equation no. 6 for plant available phosphorus (**g**) Equation no. 7 for phenol oxidase activity (**h**) Equation no. 8 for sulfide-sulfur.

We analyzed the eco-physiological resilience data from 4922 individuals comprising 16 mangroves and 15 mangrove associate species (for physiological analyses) and leaves of 603 individuals of 8 mangrove and 3 mangrove associate species (for tissue distribution analyses) from Indian Sundarbans. We performed regression analysis for a smaller representative dataset of sample size *n* = 135 (as we have the lowest number of observations for variable pinitol assay) (Table S6B) and conceived the linear regression models including all the factors of eco-physiological resilience (Table 2A). This regression analysis was comprised of finding most efficient linear model over a very large number of plausible linear regression models as the number of predictors (i.e. eco-physiological resilience) here is very large. For this model selection problem, we adopted an efficient modern technique called Bayesian Adaptive Sampling. The package, BAS, designed in R-programming environment, uses an adaptive sampling algorithm to sample without replacement from the class of all possible models or MCMC sampling which is recommended for sampling problems with a large number of predictors. To identify the best suitable model over a very large number of competitive models in Bayesian linear regression analysis, we used the Fig. 3A obtained through the ‘BAS’ package in R programming. This figure was based on the fitted Bayesian linear regression equations (last column, Table 2A). From each of the eight subfigures in the Fig. 3A, the first regression model (referred by 1^st^ column, Fig. 3A) was selected as it showed first ranking due to maximum Log Posterior Odds over all the competitive models. It was found that among the all variables, the osmolytes and WST/LT, WST primarily contributed to predict most of all the degradation components (3^rd^ column, Table 2A). Posterior probabilities, given in the fourth column of Table 2A, had depicted as measures of significance of the respective predictors in all of the eight models for eight degradation determinants. Higher posterior probability implies higher significance of the respective predictor in a model. *R*^2^ values for all the regression equations were valid enough to qualify for a distinct linear relationship. Based on the fitted linear regression equations (last column, Table 2A), we prepared the observed versus predicted graph for each of the eight degradation components from different forests (Fig. 3B). The observed versus predicted plots were also found to be very linear for each of the linear regression models with χ^2^ value at significance ≤0.001, and the corresponding *R*^2^ values are close to 1. Interestingly, gene expression levels for osmolyte biosynthesis were also found to hold a very significant linear relationship with degradation criteria in Bayesian regression models (Table 2B) except for the response variable – soil conductivity. This analysis supported strongly the linear relationship of osmolyte acclimation with degradation factors and also made us conclude that the osmolyte biosynthetic gene expression individually also possessed great potential to predict the degraded mangrove ecosystem criteria.

**Table 2:** (A) Bayesian linear regression models showing the relationships between the predictor eco-physiological resilience (explanatory variables) and the degradation determinants (response variables) along with the posterior probabilities of the significance of linear model parameters. Estimates of the linear model parameters are obtained by posterior means.

Equation no.	Degradation determinants (Response variables)	Eco-physiological resilience (Predictors) in addition to intercept	Posterior probabilities of inclusion of each of all predictors	*R*^*2*^	Posterior probabilities of the model	Regression equation
(**A**)						
**1**	Forest coverage	Intercept, WST, GB, SS, FAA	1.000, 0.470, 0.723, 0.976, 0.957	0.884	0.035	Forest coverage = 48.89 − 0.039*WST + 0.908*GB − 3.402*SS − 2.298*FAA
**2**	Ammonia-nitrogen	Intercept, PT, PT/ST, WST/LT, INO, FAA	1.000, 0.972, 0.927, 0.857, 0.946, 0.959	0.844	0.241	Ammonia-nitrogen = 3.171 − 0.021*PT + 0.009* PT/ST − 2.488* WST/LT − 0.029*INO − 0.144* FAA
**3**	Organic carbon	Intercept, LT, PT/ST, ST/LT, PINI, INO, FAA	1.000,0.709,0.954, 0.663, 0.898,	0.695	0.031	Organic carbon = 0.799 − 0.001* LT + 0.000* PT/ST + 0. 143* ST/LT − 0.007*INO − 0.013* FAA
**4**	Tidal water conductivity	Intercept, WST/LT, PRO, INO, FAA	1.000,0.908, 0.992, 0.647, 0.823	0.645	0.078	Tidal water conductivity = 39.63 + 16.48* WST/LT + 2.521* PRO + 0.092*INO − 0.649*FAA
**5**	Soil conductivity	Intercept, LT, WST, PRO, SS, FAA, MAN	1.000, 0.457, 0.599, 0.999, 0.562, 0.655, 0.991	0.647	0.029	Soil conductivity = 13.59 − 0.007* LT + 0.016*WST + 2.132*PRO + 0.277*SS + 0.274*FAA + 0.0006*MAN
**6**	Plant available phosphorus	Intercept, PT, PT/ST, WST/LT, INO, FAA	1.000, 0.538, 0.997, 0.496, 0.793, 0.888	0.764	0.037	Plant available phosphorus = 5.842–0.016*PT + 0.029*PT/ST − 0.771*WST/LT − 0.042*INO − 0.232*FAA
**7**	Phenol oxidase activity	Intercept, PT, PT/LT, SS	1.000, 0.783, 0.814, 0.758	0.734	0.035	Phenol oxidase activity = 0.628 − 0.005*PT + 1.18*PT/LT − 0.035*SS
**8**	Sulfide-sulfur	Intercept, WST, ST, PT/ST, GB, PRO, FAA	1.000, 0.824, 0.726, 0.476, 0.775, 0.901, 0.999	0.808	0.040	Sulfide-sulfur = 3.423 + 0.024*WST – 0.023*ST – 0.006*PT/ST − 0.085*GB + 0.46*PRO + 0.375*FAA
(**B**)						
**Equation no**.	**Degradation determinants (Response variables)**	**Gene variables (Predictors) in addition to intercept**	**Posterior probabilities of inclusion of each of all predictors**	***R***^***2***^	**Posterior probabilities of the model**	**Regression equation**
**9**	Forest coverage	Intercept, *F16BP*, *F26BP*, *SUS*	1.000, 0.999, 0.894, 0.853	0.927	0.536	Forest coverage = 48.886 − 3.467*F16BP − 1.417*F26BP − 2.023*SUS
**10**	Ammonia-nitrogen	Intercept, *P5CS*, *F16BP*	1.000, 0. 453, 0.993	0.822	0.241	Ammonia-nitrogen = 3.171 − 0.038*P5CS − 0.269*F16BP
**11**	Organic carbon	Intercept, *F16BP*, *FBA*	1.000, 0.994, 0.491	0.638	0.234	Organic carbon = 0.799–0.035*F16BP – 0.009*FBA
**12**	Tidal water conductivity	Intercept, *BADH*	1.000, 0.994	0.679	0.584	Tidal water conductivity = 39.633 + 0.949*BADH
**13**	Soil conductivity	Intercept, *BADH*, *FBA*	1.000, 0.999, 0.592	0.590	0.253	Soil conductivity = 13.585 + 0.730*BADH − 0.279*FBA
**14**	Plant available phosphorus	Intercept, *F16BP*, *FBA*	1.000, 0.999, 0.682	0.772	0.289	Plant available phosphorus = 5.842 − 0.510*F16BP − 0.148*FBA
**15**	Phenol oxidase activity	Intercept, *F16BP*, *F26BP*	1.000, 0.976, 0.519	0.746	0.212	Phenol oxidase activity = 0.628 − 0.050*F16BP − 0.014*F26BP
**16**	Sulfide-sulfur	Intercept, *MIPS*, *F26BP*	1.000, 0.980, 0.803	0.780	0.470	Sulfide-sulfur = 3.423 + 0.221*MIPS + 0.138*F26BP

(B) Bayesian linear regression models showing the relationships between the predictor gene expression variables (explanatory variables) and the degradation determinants (response variables) along with the posterior probabilities of the significance of linear model parameters. Estimates of the linear model parameters are obtained by posterior means.

### Mixed effects models and prediction of degradation components

Generalized linear mixed effect models were formulated (Tables 3, S3) from the same dataset (Table S6A and B) on which Bayesian linear regression was applied. It predicted different degradation criteria as response component with WST (factorized) as random factor, LT (factorized) and free amino acid (factorized) as fixed factors and soluble sugar and ST/LT as covariates under explanatory components. In linear mixed-effects model, the correlations between the degradation criteria and the eco-physiological resilience were exploited to model the degradation status. The results indicated that the most of the models have *R*^2^ values closer to 1, justifying the validity of predictive ability of the respective models (Tables 3, S3). The utilization of both the random effects and fixed effects along with covariate effects increased the prediction accuracy of the models and helped to explain the underlying heterogeneity in the data on different degradation criteria.The presence of WST and LT as random factor and fixed factor respectively in all the models implied the predictive potential of these two eco-physiological resilience factors. Similarly, consistent inclusion of free amino acids and soluble sugars also in the models explained the significance of osmotic acclimation physiology of mangroves having adequate prediction ability to intimate the degradation status of a mangrove forest.

**Table 3:** Summary of the analysis of variance in Generalized linear mixed effect models on different degradation determinants (response variables) with the five common eco-physiological resilience (explanatory) components – (*i*) WST (factorized) as random factor; (*ii*) LT (factorized) and (*iii*) Free Amino Acid (factorized) as fixed factors; and (*iv*) Soluble Sugar and (*v*) ST/LT as covariates. WST- water storage tissue, LT - leaf thickness, ST/LT- spongy tissue–leaf thickness ratio. Significant values (*P* < 0.05) are depicted in bold.

Degradation determinants (Response Variables)	Test-statistics values for different explanatory components (eco-physiological resilience) and associated p-value for corresponding hypothesis testing												*R*^2^
	Constant		WST (Random factor)		LT (Fixed factor)		Free Amino Acid (Fixed factor)		Soluble Sugar (Covariate)		ST/LT (Covariate)		
	T	*p*	F	*p*	F	*p*	F	*p*	F	*p*	F	*p*	
Forest coverage	17.07	**0.000**	13.78	**0.000**	10.48	**0.000**	3.33	**0.013**	29.43	**0.000**	2.58	0.111	0.942
Ammonia-nitrogen	12.21	**0.000**	13.75	**0.000**	0.85	0.430	1.03	0.396	6.37	**0.013**	8.35	**0.005**	0.890
Organic carbon	12.34	**0.000**	2.51	**0.019**	1.35	0.264	0.63	0.641	5.54	**0.020**	3.10	0.081	0.640
Tidal water conductivity	15.39	**0.000**	4.66	**0.000**	0.44	0.644	1.47	0.215	0.02	0.899	0.52	0.474	0.667
Soil conductivity	5.61	**0.000**	4.35	**0.000**	2.05	0.133	3.78	**0.006**	9.31	**0.003**	1.83	0.178	0.643
Plant available phosphorus	11.06	**0.000**	4.13	**0.000**	0.74	0.478	0.56	0693	15.29	**0.000**	1.15	0.287	0.760
Phenol oxidase activity	7.92	**0.000**	3.87	**0.001**	0.70	0.496	1.76	0.141	0.82	0.368	1.08	0.300	0.816
Sulfide-sulfur	5.70	**0.000**	1.38	0.219	3.68	**0.028**	2.32	0.061	0.06	0.813	2.37	0.127	0.832

### RDA analyses

**Figure 4:**
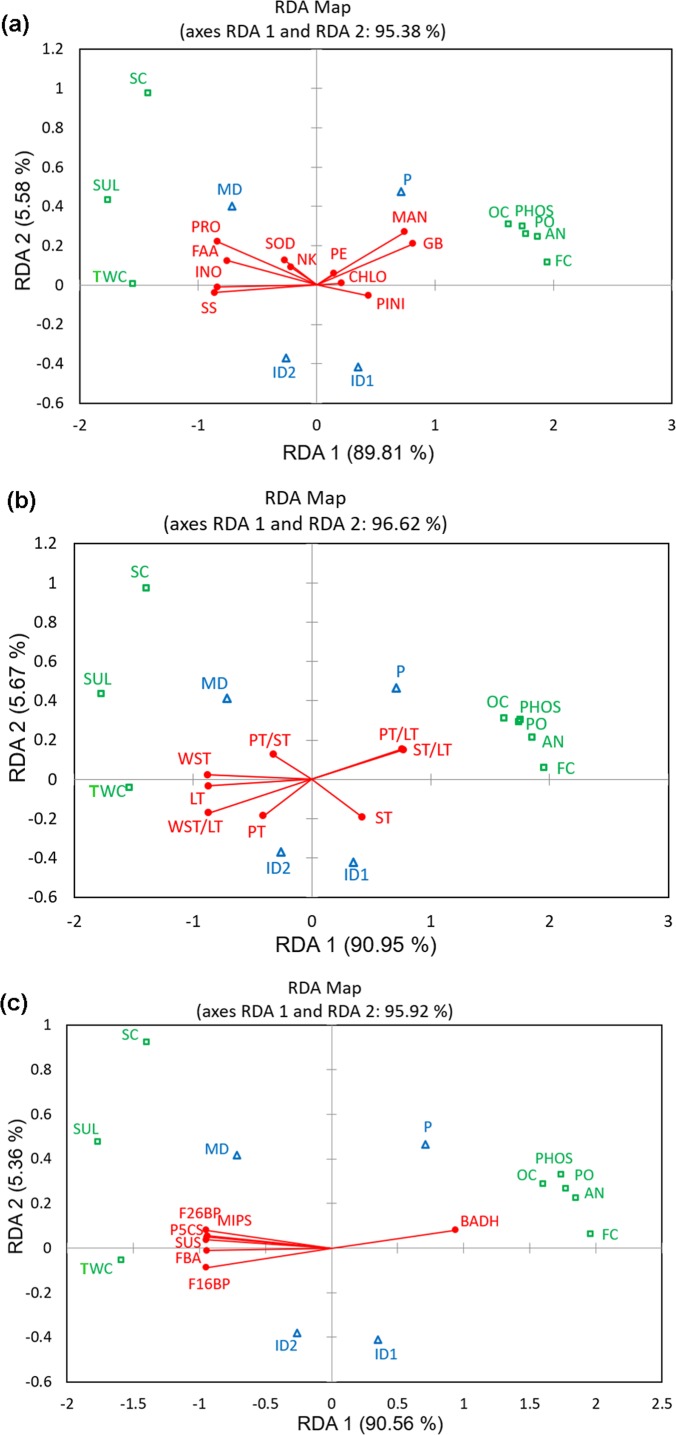
Biplot generated by canonical redundancy analysis (RDA) illustrating the effects of degradation factors (response variables) on the eco-physiological resilience components (explanatory variables). Response variables (*in green*): SC-soil conductivity, SUL-sulfide-sulfur, TWC-tidal water conductivity, PHOS-plant available phosphorus, OC-organic carbon, AN-NH_4_-N, PO- phenol oxidase activity, FC-forest coverage. Explanatory Variables (Qualitative) (*in blue*): P-pristine, ID1-Intermediate degradation 1, ID2-Intermediate degradation 2 and MD-Maximal degradation. Different set of Explanatory variables (Quantitative) in three different figures (*in red*): (*a*) PRO-proline, FAA-free amino acids, INO-inositol, SS-soluble sugar, MAN-mannitol, GB- glycinebetaine, PINI-pinitol, PE- activity ratio of PEPC/RUBPC, CHLO-total chlorophyll concentration, SOD-superoxide dismutase activity, NK-total Na^+^/K^+^ ratio; (*b*) LT- leaf thickness, PT-palisade tissue thickness, ST -spongy tissue thickness, WST -water storage tissue thickness, PT/ST- palisade–spongy tissue thickness ratio, PT/LT -palisade tissue–leaf thickness ratio, ST/LT -spongy tissue–leaf thickness ratio, WST/LT- water storage tissue-leaf thickness ratio; (*c*) gene expression: *P5CS* for proline synthesis, *BADH* for glycinebetaine synthesis, *MIPS* for myo-inositol synthesis, *SUS*, *F1, 6BP*, *F2, 6BP*, *FBA* for soluble sugar synthesis.

We performed canonical redundancy analysis (RDA) to explain the interactive display of key degradation parameters having influence on the eco-physiological resilience components. Figure 4a–c explained 95.38%, 96.62% and 95.92% of total variations respectively. In all the three RDAs, constrained variances are much higher than the unconstrained variances (Table S4) that suggests much of the variation in the response variables are “redundant” (i.e. “explained” by) and accounted for by the set of explanatory variables of resilience components. In all the Fig. 4a–c, all the degradation components are found to be associated closely to the eco-physiological parameters proving high correlation between these two sets of data. Validating the Pearson’s analysis all the osmolytes except mannitol and glycinebetaine maintained positive correlation with tidal water and soil conductivity and sulfides at the maximally degraded sites. With all other degradation criteria they showed negative correlation at the pristine forest sites (Fig. 4a). Mannitol and glycinebetaine displayed the reverse (Fig. 4a) relationship. Figure 4a projected all the osmolytes as the major contributors of eco-physiological resilience. The short lengths of arrows for SOD activity, total chlorophyll, activity ratio of PEPC/RuBPC, total Na^+^/K^+^ ratio, pinitol accumulation, all indicated their less significance as key contributors of eco-physiological resilience trait complex (Fig. 4a). In Fig. 4b, WST, LT and WST/LT demonstrated strong positive correlation with tidal water and soil conductivity and sulfides but negative correlation with all other degradation factors. On the other hand PT/LT and ST/LT indicated the exact opposite pattern. PT, ST and PT/ST indicated by comparatively shorter arrows represent insignificant contribution towards eco-physiological resilience trait complex. Gene expression exactly corresponded with the respective osmolyte parameters (the genes responsible for the biosynthesis of respective osmolytes) (Fig. 4c). The resilience data though are correctly discriminated into four degradation stages by CDFA, expression of resilience is more closely associated with the pristine (control) and maximally degraded habitats.

### Succulence, a visible eco-physiological resilience trait

Increase in water storage tissue across increasing degradation gradient increases leaf thickness and in turn generates succulence in mangrove leaves, a major resilience mechanism to assure sustenance of mangroves with salinity rise. In Fig. S2, a sharp increase is observed for WST by modification of hypodermal layers and its extension beyond vascular tissue in the central region of transverse sections of leaves for eight mangroves and associate species in maximally degraded mangrove forests (S2a,c,e,g,i,k,m,o) in comparison to control pristine mangrove habitats (S2b,d,f,h,j,l,n,p). It unequivocally demonstrates this resilience trait to be most visibly linked against degradation amongst all other studied eco-physiological resilience components.

## Discussion

**Figure 5:**
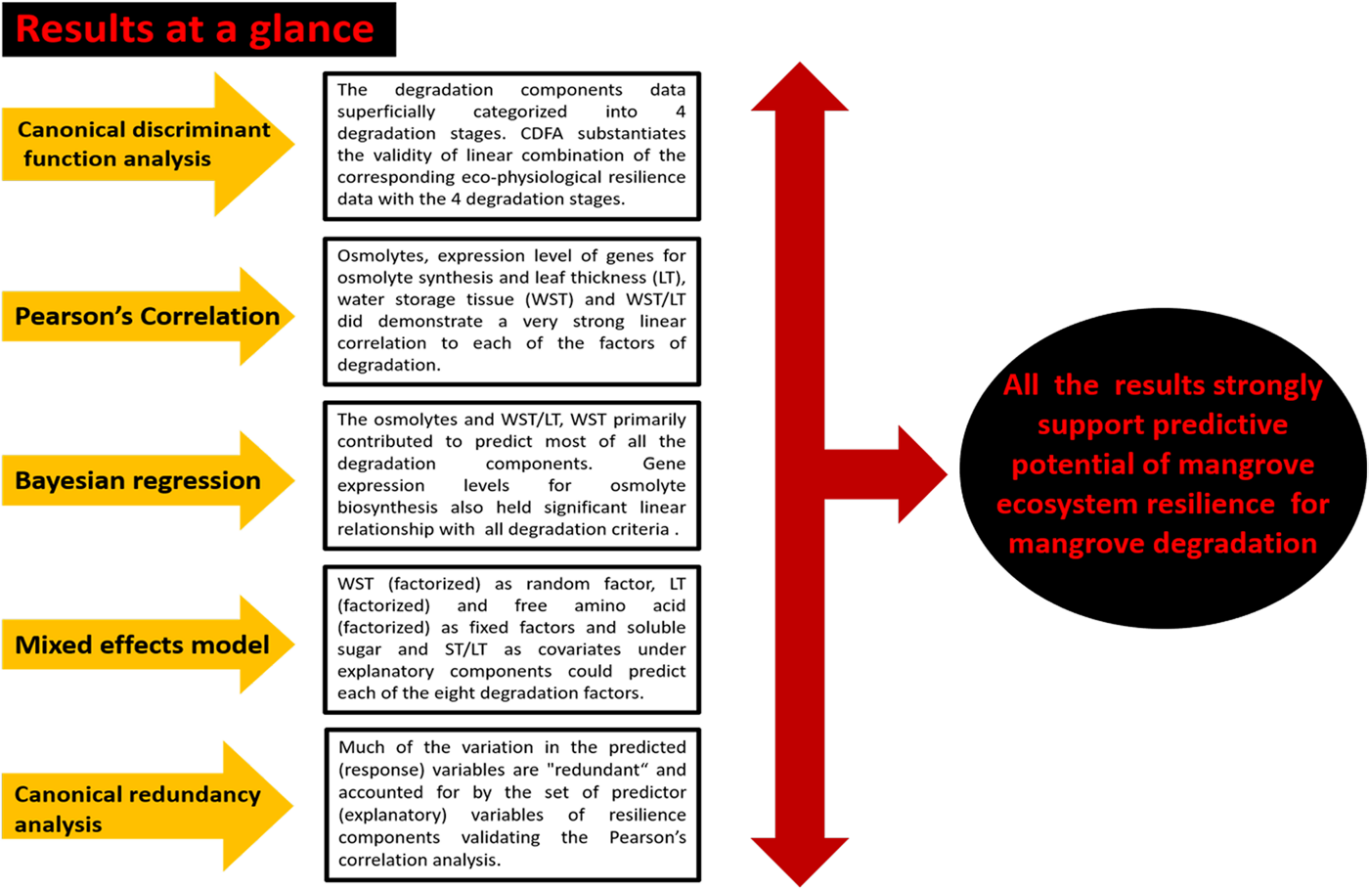
The results of the study conducted in a nutshell. The eco-physiological resilience data developed from 4922 individuals (physiological and gene expression analyses) and 603 individuals (tissue distribution analyses) of 16 mangroves and 15 mangrove associate species are statistically modelled with osmolyte accumulation, gene expression for osmolyte biosynthesis, water storage tissue (WST) and leaf thickness (LT) as efficient predictors of mangrove ecosystem degradation. Data on eight degradation determinants from 19 mangrove forests displayed significantly strong linear relationship with corresponding ecosystem resilience validating the predictive potential of our proposed statistical models.

With the advent of statistical methodology, the development of predictive habitat forecasting models has rapidly increased in ecology. Such models are static and probabilistic in nature, since they statistically relate the species or communities to their present environment. A wide range of predictive models has been developed to involve areas as diverse as biogeography, conservation biology, climate change research, and habitat or species management^14^. Multiple regression and mixed effect models are very popular and are often used for species distribution predictive geographical modeling. These models are predictive tools to assess the impact of environmental change on the distribution of organisms, to test biogeographic hypotheses, to improve floristic and faunistic atlases, or to set up conservation priorities^14^. By model formulation, we deduce a suitable algorithm for predicting response variables and for estimating the model coefficients. Our regression models relate response variables as factors of mangrove ecosystem degradation to a combination of ecosystem resilience predictors (explanatory variables)(Fig. 5). The same approach was followed for mixed effect modeling on the same dataset after considering some variables as factors (e.g., WST, LT, free amino acid) and categorized the respective data accordingly (Fig. 5). Both types of predictive models seemed to be strongly appropriate for our dataset. These models are fundamentally probabilistic in nature and their most ideal application was aimed for prediction purposes of mangrove ecosystem degradation. Most published static modeling studies use only one of the many statistical methods and little information is available on the respective predictive capacity of their approach^14^. We successfully applied two different statistical modeling techniques based on the same explanatory variables and our observed versus predicted data well demonstrate the respective predictive potential of each of the models (Fig. 5).

There has been major progress in the development of species distribution models (SDMs) that can link species distribution abundances to environmental data^15–19^. A major barrier to the development of eco-physiologically grounded SDMs has been in linking data on the limiting behavioural, morphological and physiological traits of organisms with GIS datasets on climate and sited locations^15–19^. In our models we directly incorporated the eco-physiological resilience as explanatory variables and predict degraded environment as response variables (Fig. 1). Organisms are most responsive to environmental conditions and it is quite logical to involve their eco-physiological consequences to derive mechanistic models; hence eco-physiological processes are now being used in conjunction with GIS data on climate and terrain to make inference on species distributions^15–19^. While there have been some attempts to incorporate physiological processes into species distribution models in both the plants and animals, typically they have not been accounted for this dynamic interaction between organism and environment^15–19^. The objective of this whole research was utilization of integrated eco-physiological cues to deterministically predict appropriate response towards degradation of a forest; these responses have direct contribution to mangrove forest degradation. Our hypothesis was to predict the environmental threats of a mangrove forest before it is too late for taking protective action. The resilience mechanism initiates in mangroves long before the environment becomes actually degraded. Exceeding the threshold of resilience, results in actual degraded situation. But this threshold is unknown to us. Our studies only assessed the eco-physiological resilience of mangroves under four distinct hypothetical degradation states and tried to fit them in predictive models (Fig. 5).

The selection of eight environmental factors for evaluating degradation status of a mangrove ecosystem demonstrates an integrated environment typical to mangroves. Ammonia-nitrogen measured here is the primary available form of soil nitrogen in mangrove ecosystem^20–22^ whereas organic carbon is a well established indicator for evaluating soil fertility of any ecosystem. Plant available phosphorus in mangrove forest sediments is a prime limiting factor for mangrove growth^23^. Soil and tidal water conductivity is an indicator of salinity of the ecosystem, a significant component of mangrove environment. Phenol oxidase activity has been used here as a determinant of anoxic environment as this enzyme acts only aerobically and oxygen constraints on this enzyme can minimize the activity of other hydrolytic enzymes responsible for decomposition and nutrient cycling^24^. Oxygen constraints and anaerobic condition created by tidal water logging is indispensable in mangrove ecosystems. Sulfide deposition in mangrove sediments is characteristically operated by anaerobic sulfate reducing bacteria and is again dependent on anaerobic condition caused by water logging in mangrove environment. This sulfide is considered to be toxic for mangrove colonization^25^. Forest cover of any forest is an obvious deterministic criterion to understand the intensity of degradation. Increasing degradation of mangrove forests could be assigned due to decline of forest cover and ammonia nitrogen, organic carbon, plant available phosphorus, phenol oxidase activity in sediments along with simultaneous increase in soil and tidal water conductivity and sulfide accumulation^3^.

The composite eco-physiological resilience traits evaluated in this study are fundamental to mangrove ecosystem functioning. One of the key physiological features in salinity tolerance of mangroves is their ability to maintain a low cytosolic Na^+^/K^+^ ratio by directing the excess cytosolic Na^+^ to the vacuole^26^. In low water potential niche, common protective response in mangroves is to accumulate a variety of compatible solutes which are zwitterionic and that do not interfere with metabolism and cause osmotic acclimation^27,28^. These are called osmolytes that accumulate in cytosol as well as in cell organelles^29^. Among the osmolytes, glycinebetaine is the most abundantly accumulated QAC occurring in plants, mainly localized in chloroplasts and plays a vital role in chloroplast osmotic adjustment protecting the photosystem II complex^29^. All the free amino acids, especially proline is osmotically very active, contributes to membrane stability and serves as a sink for excess reductants^28,29^. Soluble sugars contribute up to 50% of the total osmotic potential for osmotic adjustment^29^. The accumulation of polyols like mannitol, myo-inositol and pinitol facilitate osmotic acclimation promoting scavenging for reactive oxygen species^29^. In addition, expression of seven genes responsible for osmolyte biosynthesis was considered in this study to support the osmolyte accumulation resilience. *MIPS*, *BADH* and *P5CS *are responsible for biosynthesis of myo-inositol, glycine-betaine and proline respectively. Cytosolic *F1, 6BP*, *FBA*, *F2, 6BP*, *SUS* plays the more important role in balancing the allocation of carbon between sucrose and starch and favors sucrose synthesis in the cytosol. Apart from osmolytes, under typical mangrove environment of high temperatures and high salinity, stomata remains open for only a short period to prevent hydraulic dysfunction^30^. Hence, faster photosynthetic carboxylation by PEPC becomes an inevitable necessity and has been reported to be a natural choice for carbon assimilation in mangroves^30^. This dependence of mangrove species on PEPC can be estimated quantitatively from the activity ratio of PEPC and RuBPC. It is also reported that mangroves have less chlorophyll than other glycophytic species because of the ‘dilution’ of chlorophyll by presence of excess water storage tissue (succulence) in leaves^30^. This limiting chlorophyll content also supports the need for a faster and more efficient CO_2_ assimilation^30^. In addition, under stressed environments natural photoprotection by superoxide detoxification is carried out by superoxide dismutase (SOD) activity^31^. Palisade and spongy tissues are the main custodians of photosynthesis because of their higher chlorophyll content. But the presence of water storage tissue arising mostly out of modified hypodermal layers is a significant adaptive resilience in mangrove species as a signature of their extremophilic lifestyle. Water storage tissue as known by its name helps the mangroves to display resilience in physiologically dry high saline ecosystem by storing water and maintaining succulence^32^. These multitudes of eco-physiological traits across degradation gradients are assumed to respond to buffer the ecological perturbation.

Mangrove ecosystems occupy one of the most human-affected regions of the world, the continent-ocean interface. The combined effects of anthropogenic and natural stressors jeopardize the role of mangroves as a functional habitat providing vital ecosystem services. About 90% of the global mangroves are growing in developing countries of South-East Asia and South America and they are at great risk facing degradation and fragmentation of the habitats^33^. Degradation of mangrove ecosystem is widespread and till date has been reported from countries like Malayasia^34^, Indonesia^35^, Ecuador^36^, Philippines^37^, Sri Lanka^38^, Brazil^39^, Pakistan^40^ and China^41^. The conservation of the mangrove environment and its ecosystem services depends upon identifying regions that are at great risk and need to be saved from negative impact of further degradation. The prediction of the mangrove habitat degradation is conclusively established by our predictive models (Fig. 5). These resilience traits have the potential to provide an early indication about the cryptic operation of ecosystem stressors. To the best of our knowledge this is the first attempt to use the eco-physiological trait complex as distinctive predictors of ecosystem degradation. The early detection of ecosystem degradation would obviously facilitate the policy makers to be on guard and expedite the programs for ecosystem restoration. We assume the predictors to be applicable similarly for all the mangrove forests of the world that are confronted with threats of degradation.

## Methods

### Site description

Our study area between 21°40′0″N and 22°0′0″N and 88°20′0″E to 88°30′0″E was located in the western part of Indian Sundarbans (Fig. 2). The studied area was situated on the shorelines of an intricate river system network. We surveyed 19 mangrove forests^3^ (Table S1) and sediments as well as leaf samples were collected from these regional islands. All surveyed sampling points are shown in Fig. 2. The studied mangrove forests are found to be at different degradation stages, starting from pristine ecologically undisturbed condition to exceedingly disturbed degraded state^3^ as judged on the basis of the studied degradation criteria (Table S1). We have apparently grouped the forests under four categories such as pristine (control), intermediate degradation 1, intermediate degradation 2 and maximally degraded state (Table S1).

### Measuring evaluators of mangrove ecosystem degradation

Five composite sediment samples (composited out of ten cores) from 0–60 cm depths from each forest were collected^3^ and brought to the laboratory keeping in ice-box. A part of soil was stored at 4 °C for phenol oxidase activity and sulfide estimation with rest of the part air dried at room temperature (28 °C). NH_4_-N was assayed on extraction by 2 M KCl and using phenate method^42–44^ measuring absorbance at 640 nm. Organic carbon was determined by spectrophotometric method by measuring the amount of carbon in sucrose present in the soil^45^. 1 g of composite sediment sample each from vertical profile 0–15 cm, 15–30 cm, 30–45 cm, and 45–60 cm was reacted with 1/6 M K_2_Cr_2_O_7_ and concentrated sulfuric acid containing 1.25% Ag_2_SO_4_^45^. The suspension developed a green color when incubated for 30 min, and the color intensity was measured at 660 nm. Soluble P was extracted with modified Morgan extractant^46^ and assayed by molybdenum-blue method^47^. Conductivity of the tidal water and sediment cores was measured^3^ from each of the 19 mangrove forests by conductivity meter (Chemiline CL250, Labline Technology Pvt. Ltd., Ahmedabad, India). Phenol oxidase activity was determined by oxidation of L-DOPA^48,49^. Liberation of sulfide was assayed by phosphoric acid steam distillate^50^. All the spectrophotometric studies were performed using SmartSpec Plus spectrophotometer (Bio-Rad, California, USA). All the stated environmental variables were evaluated from both dry and wet seasons. Forest cover of the mangrove forests was measured from 10 random quadrats of dimensions 10 m × 10 m each from the referred 19 forests. The average Tree Basal Area (TBA) for each species was multiplied with its total number of individuals and expressed as a percentage to determine final percent forest coverage^3^.

### Evaluation of mangrove eco-physiological trait complex

Leaf samples from reproductively mature total 4922 individuals of 16 true mangrove species and 15 mangrove associate species were collected from 19 mangrove forests and were preserved at 4 °C until assayed for osmolytes and enzyme activities. Some of the collected specimens of each sample were air dried for estimation of total Na^+^ and K^+^ content. Air-dried leaf samples extracted with 1 N ammonium acetate solution (pH 7.0) and incubated for 24 hours were used for Na^+^ and K^+^ determination by flame photometer (Frontline, India) calibrated with 0–100 ppm of standard solutions of NaCl and KCl. Proline assay was performed^51^ by selective extraction with aqueous sulfosalicylic acid. Assay for glycine-betaine was carried out^52^ based on the fact that at low temperature betaine made a betaine-periodite complex with iodide in acidic medium. Mannitol was assayed^53^ by oxidation with sodium periodate in presence of sodium metathiosulfate. Soluble sugar and starch was quantified^54^ in hot acidic medium where soluble sugars were dehydrated to hydroxymethyl furfural. Free inositol was assayed^55,56^ from air dried plant tissue extracted with 0.04 N HCl. Total free amino acids were assayed^57^ by reaction with ninhydrin, that decarboxylated the alpha-amino acids. Pinitol estimation was carried out by a modified protocol^58,59^ using gas chromatography (GC). PEPC was assayed^60^ by extracting the plant enzyme in 100 mM MOPS extraction buffer (pH 7.4) with 5 mM DTT, BSA (10 mg ml^−1^) and polyvinylpolypyrrolidone (100 mg ml^−1^). RuBPC activity was assayed at 30 °C^61^. Both PEPC and RuBPC activity are expressed as µmol NADH oxidized min^−1^ µg^−1^ of protein. For chlorophyll estimation, the fresh leaves were extracted with 100% acetone, absorbance at 662 nm and 645 nm was measured and pigment concentration was calculated using standard formulae^62^. Superoxide dismutase activity assay was performed^63,64^ where nitro blue tetrazolium (NBT) was used to intercept O_2_^−^ generated photochemically with riboflavin in presence of light. All the spectrophotometric studies were performed using SmartSpec Plus spectrophotometer (Bio-Rad, California, USA).

Leaf samples of mature total 603 individuals of 8 true mangroveand 3 mangrove associate species were collected in 10–12 replicates from each study site and were preserved in formaldehyde, acetic acid and 50% ethanol (FAA) solution (1:1:18, v/v). Free hand transverse sections of fixed leaf samples were obtained using razor blades and the sections were stained with 0.1% toluidine blue solution. All the traits (PT, ST, LT, and WST) were measured in μm and observations were performed using trinocular research microscope Dewinter Classic 1624424, Dewinter India and compatible DGI 510 CCD camera and Digicam software.

### Gene expression by Quantitative Real Time PCR

We utilized published Real Time PCR primer for reference gene 18S rRNA, as the control gene for the expression study^65^ (Table S1). Due to inadequate mangrove species specific mRNA/cDNA sequences in NCBI database for our target seven genes, we relied upon the same from very close relatives of the genera to design primers with multiple alignments by CLUSTALW from conserved region. The primers were designed with the help of the SCITOOLS site of Integrated DNA Technologies (https://eu.idtdna.com/scitools/applications/realtimepcr/) (Table S5). The total RNA was extracted from portion of the leaves of 7 true mangroves and11 mangrove associate species with Purezol RNA isolation reagent (Bio-Rad Laboratories, Hercules, California, US) and was quantified by the NanoDrop UV spectrophotometer (Eppendorf Company, Eppendorf, Hamburg, Germany). 1 μg RNA from each sample was taken for immediate cDNA synthesis by a cDNA synthesis kit using random hexamer approach (BioBharati LifeScience Private Limited, Kolkata,West Bengal, India). 1 μl of each cDNA was used for quantitative real-time PCR (QRT PCR) using iTaq Universal SYBR Green Supermix (Bio-Rad Laboratories, Hercules, California, US). The PCR conditions were set as follows: Pre-incubation (1 cycle) −94 °C for 7 minutes; 3 step amplification (45 cycles) −94 °C for 15 seconds, 55 °C for 40 seconds, and 72 °C for 30 seconds; melting (1 cycle) −95 °C for 10 seconds, 65 °C for 60 seconds, and 97 °C for 1 seconds. Final data were analyzed and accurate normalization was done with reference gene^66^.

### Statistical analyses

All the experimental evaluations were carried out for three biological replicates each with three technical replicates. All the graphical presentations were prepared in SigmaPlot 13.0 software. A canonical discriminant function analysis (CDFA) was performed to authenticate eco-physiological resilience data categorization corresponding to four different hypothetical degradation states. All the studied environmental parameters of degradation were correlated with eco-physiological variables evaluated across 19 mangrove forests using Pearson’s Correlation Coefficient analysis to measure of the strength of the association between the two variables (in SPSS 25.0 software). Bayesian linear regression analysis was performed in R-programming software (version 3.6.1) to predict each degradation factor based on eco-physiological resilience components (predictors) across different degradation states. For this purpose, we adopted Bayesian Adaptive Sampling (BAS) package in R-programming to establish the most suitable regression model among the all possible linear regression models based on model averaging technique. Linear mixed effects models were formulated to assess the effect of experimental factors (eco-physiological variables) on the dependable variables (degradation determinant variables) with WST (factorized) as random factor, LT (factorized) and free amino acid (factorized) as fixed factors, and soluble sugar and ST/LT as covariates. All linear mixed effect models were estimated using the restricted maximum likelihood method performed in Minitab version 19.0. Redundancy analysis (RDA) was conducted to extract and summarize the variation in a set of response variables that can be explained by a set of explanatory variables performed in XLSTAT 19 software.

## Data Availability

All data generated or analyzed during this study are included in this published article and its Supplementary Information file.

## Supplememtary infromation

Supplememtary infromation.

## References

[1] Sarker SK, Reeve R, Thompson J, Paul NK, Matthiopoulos J (2016). Are we failing to protect threatened mangroves in the Sundarbans world heritage ecosystem?. Scientific Reports.

[2] Cavanaugh KC (2015). Integrating physiological threshold experiments with climate modeling to project mangrove species’ range expansion. Global Change Biology.

[3] Chowdhury R (2019). Effects of nutrient limitation, salinity increase, and associated stressors on mangrove forest cover, structure, and zonation across Indian Sundarbans. Hydrobiologia.

[4] Kathiresan K (2018). Mangrove forests of India. Current Science.

[5] Dahdouh-Guebas F (2005). Transitions in ancient inland freshwater resource management in Sri Lanka affect biota and human populations in and around coastal lagoons. Current Biology.

[6] Holling CS (1973). Resilience and stability of ecological systems. Annual Review of Ecology and Systematics.

[7] Gunderson LH (2000). Ecological resilience: in theory and application. Annual Review of Ecology and Systematics.

[8] Fischer J (2009). Integrating resilience thinking and optimisation for conservation. Trends in Ecology and Evolution.

[9] Scheffer M, Carpenter S, Foley JA, Folke C, Walker B (2001). Catastrophic shifts in ecosystems. Nature.

[10] Dent CL, Cumming GS, Carpenter SR (2002). Multiple states in river and lake ecosystems. Philosophical transactions of the Royal Society of London B.

[11] Scheffer M, Carpenter SR (2003). Catastrophic regime shifts in ecosystems: linking theory to observation. Trends in Ecology and Evolution.

[12] Petraitis PS, Dudgeon SR (2004). Detection of alternative stable states in marine communities. Journal of Experimental Marine Biology and Ecology.

[13] Gutschick VP, BassiriRad H (2003). Extreme events as shaping physiology, ecology, and evolution of plants: toward a unified definition and evaluation of their consequences. New Phytologist.

[14] Guisan A, Zimmermann NE (2000). Predictive habitat distribution models in ecology. Ecological Modelling.

[15] Kearney M, Porter W (2009). Mechanistic niche modelling: combining physiological and spatial data to predict species’ ranges. Ecology Letters.

[16] Kearney MR, Wintle BA, Porter WP (2010). Correlative and mechanistic models of species distribution provide congruent forecasts under climate change. Conservation Letters.

[17] Kotta, J. *et al*. Integrating experimental and distribution data to predict future species patterns. *Scientific Reports* **9**, 10.1038/s41598-018-38416-3 (2019). 10.1038/s41598-018-38416-3 PMC6372580 30755688

[18] Urban, M. C. *et al*. Improving the forecast for biodiversity under climate change. *Science* **353**, 10.1126/science.aad8466 (2016). 10.1126/science.aad8466 27609898

[19] Martínez B, Arenas F, Trilla A, Viejo RM, Carreño F (2015). Combining physiological threshold knowledge to species distribution models is key to improving forecasts of the future niche for macroalgae. Global Change Biology.

[20] Twilley RW, Lugo AE, Patterson-Zucca C (1986). Litter production and turnover in basin mangrove forests in southwest Florida. Ecology.

[21] Alongi DM (1994). The role of bacteria in nutrient recycling in tropical mangrove and other coastal benthic ecosystems. Hydrobiologia.

[22] Kristensen E, Bouillon S, Dittmar T, Marchand C (2008). Organic carbon dynamics in mangrove ecosystems: a review. Aquatic Botany.

[23] Krauss KW (2008). Environmental drivers in mangrove establishment and early development: a review. Aquatic Botany.

[24] Freeman C, Ostle N, Kang H (2001). An enzymic ‘latch’ on a global carbon store. Nature.

[25] Nickerson NH, Thibodeau FR (1985). Association between pore water sulphide concentrations and the distribution of mangroves. Biogeochemistry.

[26] Flowers TJ, Munns R, Colmer TD (2015). Sodium chloride toxicity and the cellular basis of salt tolerance in halophytes. Annals of Botany.

[27] Munns R, Tester M (2008). Mechanisms of salinity tolerance. Annual Review of Plant Biology.

[28] Slama I, Abdelly C, Bouchereau A, Flowers T, Savouré A (2015). Diversity, distribution and roles of osmoprotective compounds accumulated in halophytes under abiotic stress. Annals of Botany.

[29] Ashraf M, Harris PJC (2004). Potential biochemical indicators of salinity tolerance in plants. Plant Science.

[30] Joshi, G. V., Sontakke, S., Bhosale, L. & Waghmode, A. P. Photosynthesis and photorespiration in mangroves. In:TeasH.J.(eds)*Physiology and management of mangroves. Tasks for vegetation science* 9,1-14, Springer, Dordrecht. 10.1007/978-94-009-6572-0_1 (1984).

[31] Cheeseman JM, Herendeen LB, Cheeseman AT, Clough BF (1997). Photosynthesis and photoprotection in mangroves under field conditions. Plant, CellEnvironment.

[32] Naskar, K. & Mandal, R. Ecology and biodiversity of Indian Sundarban. *New Delhi:Daya publishing house* (1999).

[33] UNEP (2014). The Importance of Mangroves to People: A Call toAction. van Bochove, J., Sullivan, E., Nakamura, T. (Eds). United Nations Environment Programme World Conservation Monitoring Centre, Cambridge. 128 (2014).

[34] Amir AA (2018). Mitigate risk for Malaysia’s mangroves. Science.

[35] Nurkin B (1994). Degradation of mangrove forests in South Sulawesi, Indonesia. Hydrobiologia.

[36] Lacerda LD, Marins RV (2002). River damming and changes in mangrove distribution. ISME /GLOMIS Electronic Journal.

[37] Primavera JH (2000). Development and conservation of Philippine mangroves: institutional issues. Ecological Economics.

[38] Katupotha, K. N. J. Degradation of mangrove swamps in Sri Lanka. *Proceedings of the Seventh Annual Forestry and Environment Symposium. University of Sri Jayewardenepura*, *Sri Lanka*, 10.31357/fesympo.v0i0.1603.g773 (2001).

[39] Ferreira AC, Lacerda LD (2016). Degradation and conservation of Brazilian mangroves, status and perspectives. Ocean &Coastal Management.

[40] Shah AA, Ibrahim K, Jusoff K (2007). Degradation of Indus Delta Mangroves in Pakistan. International Journal of Geology.

[41] Meng X, Xia P, Li Z, Meng D (2016). Mangrove degradation and response to anthropogenic disturbance in Maowei Sea (SW China) since 1926 AD: Mangrove-derived OM and pollen. Organic Geochemistry.

[42] Dorich RA, Nelson DW (1983). Direct colorimetric measurement of ammonium in potassium chloride extracts of soils. Soil Science Society of America Journal.

[43] Solórzano L (1969). Determination of ammonia in natural waters by the Phenolhypochlorite method. Limnology and Oceanography.

[44] Park G, Oh H, Ahn S (2009). Improvement of the ammonia analysis by the phenate method in water and wastewater. Bulletin of the Korean Chemical Society.

[45] Datta NP, Khera MS, Saini TR (1962). A Rapid Colorimeteric Procedure for the Determination of the Organic Carbon in the Soils. Journal of the Indian Society of Soil Science.

[46] McIntosh JL (1969). Brayand Morgan soil extractants modified for testing acid soils from different parent materials. Agronomy Journal.

[47] Krishnaswamy U, Muthusamy M, Perumalsamy L (2009). Studies on the efficiency of the removal of phosphate using bacterial consortium for the biotreatment of phosphate wastewater. European Journal of Applied Sciences.

[48] Bach CE (2013). Measuring phenol oxidase and peroxidase activities with pyrogallol, l-DOPA, and ABTS: Effect of assay conditions and soil type. Soil Biology & Biochemistry.

[49] Gallo M, Amonette R, Lauber C, Sinsabaugh RL, Zak DR (2004). Microbial community structure and oxidative enzyme activity in nitrogen-amended north temperate forest soils. Microbial Ecology.

[50] EnvironmentAgency, UK. The determination of easily liberated sulphide in soils and similar matrices. Methods for the Examination of Waters and Associated Materials, 10–17, https://assets.publishing.service.gov.uk/government/uploads/system/uploads/attachment_data/file/316780/Sulphide-228.pdf (2010).

[51] Bates LS, Waldren RP, Teare ID (1973). Rapid determination of free proline for water-stress studies. Plant and Soil.

[52] Grieve CM, Grattan SR (1983). Rapid assay for determination of water soluble quaternary ammonium compounds. Plant and Soil.

[53] Sa’nchez J (1998). Colorimetric assay of alditols in complex biological samples. Journal of Agricultural and Food Chemistry.

[54] DuBois M, Gilles KA, Hamilton JK, Rebers PA, Smith F (1956). Colorimetric method for determination of sugars and related substances. Analytical Chemistry.

[55] Darbre A, Norris FW (1956). Vitamins in germination. Determination of free and combined inositol in germinating oats. Biochemical Journal.

[56] Gaitonde MK, Griffiths M (1966). A spectrophotometric method for the determination of microquantities of free inositol in biological material. Analytical Biochemistry.

[57] Moore S, Stein WH (1948). Photometric ninhydrin method for use in the chromatography of amino acids. Journal of Biological Chemistry.

[58] Garland S, Goheen S, Donald P, McDonald L, Campbell J (2009). Application of derivatization gas chromatography/mass spectrometry for the identification and quantitation of pinitol in plant roots. Analytical Letters.

[59] McDonald LW, Goheen SC, Donald PA, Campbell JA (2012). Identification and quantitation of various inositols and O-methylinositols present in plant roots related to soybean cyst nematode host status. Nematropica.

[60] Smith AM, Hylton CM, Rawsthorne S (1989). Interference by Phosphatases in the Spectrophotometric Assay for Phosphoenolpyruvate Carboxylase. Plant Physiology.

[61] Du Y-C (1996). Animproved spectrophotometric determination of the activity of ribulose 1,5-bisphosphate carboxylase. Japanese Journal of Crop Science.

[62] Lichtenthaler, H. K. & Buschmann, C. Chlorophylls and Carotenoids: Measurement and Characterization by UV-VIS Spectroscopy. *Current Protocols in Food Analytical Chemistry*, 10.1002/0471142913.faf0403s01 (2001).

[63] Beyer WF, Fridovich I (1987). Assaying for superoxide dismutase activity: some large consequences of minor changes in conditions. Analytical Biochemistry.

[64] Beauchamp C, Fridovich I (1971). Superoxide dismutase: improved assays and an assay applicable to acrylamide gels. Analytical Biochemistry.

[65] Wang R (2011). Anatomical and Physiological Plasticity in *Leymus chinensis* (Poaceae) along Large-Scale Longitudinal Gradient in Northeast China. PLoS ONE.

[66] Livak KJ, Schmittgen TD (2001). Analysis of relative gene expression data using real-time quantitative PCR and the 2−⊿⊿Ct method. Methods.

[67] Das, G. K. Estuarine morphodynamics of the Sunderbans. Springer International Publishing Switzerland, 10.1007/978-3-319-11343-2 (2015).

