# Supplementary material for: Forecasting mangrove ecosystem degradation utilizing quantifiable eco-physiological resilience -A study from Indian Sundarbans
Source: Sci Rep. 2020 Apr 21;10:6683. doi: 10.1038/s41598-020-63586-4 (PMC7174328; doi:10.1038/s41598-020-63586-4)
Supplement: Supplementary file 1 — Supplememtary infromation. [file 41598_2020_63586_MOESM1_ESM.pdf]

Supplementary Information

Forecasting mangrove ecosystem degradation utilizing quantifiable eco-physiological resilience -A study from Indian Sundarbans

Authors:

Mst Momtaj Begam[1‡], Rajojit Chowdhury[1‡], Tapan Sutradhar[1‡§], Chandan Mukherjee[1], Kiranmoy Chatterjee[3], Sandip Kumar Basak[2*], Krishna Ray[1*]

[1]Environmental Biotechnology Group, Department of Botany, West Bengal State University, Berunanpukuria, Malikapur, Barasat, Kolkata 700126, India

[2]Sarat Centenary College, Dhaniakhali, Hooghly 712302, West Bengal, India

[3]Department of Statistics, Bidhannagar College, Salt Lake City, Sector 1, Block EB, Kolkata 700064, India

[‡] Authors contributed equally to this work

[§]Author deceased

[*]Corresponding authors

[*]Corresponding authors' email addresses:

sandipbasak9592@gmail.com, kray91@gmail.com

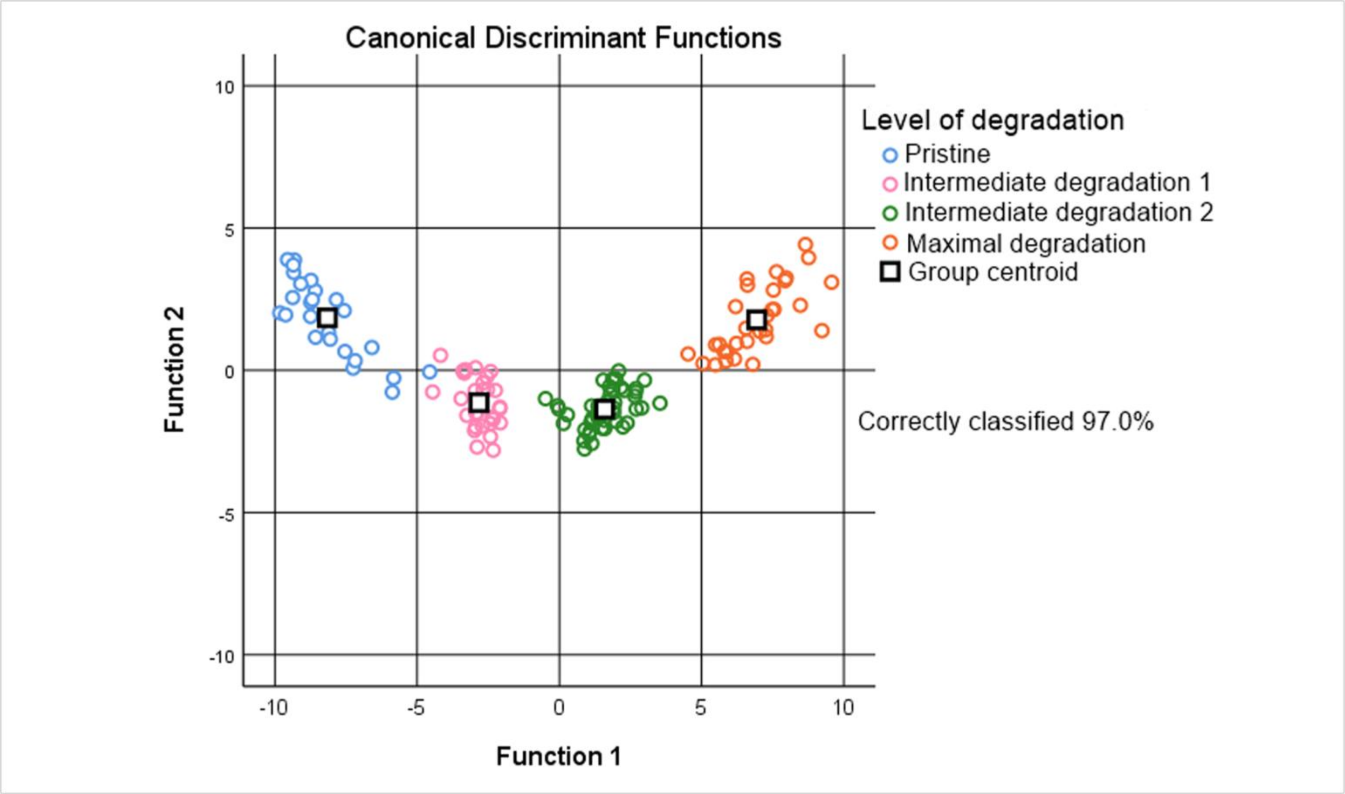

Figure S1: Canonical Discriminant Function Analysis (CDFA) of eco-physiological resilience data. 97% of this data is correctly classified under four different degradation states of pristine, intermediate degradation 1, intermediate degradation 2 and maximally degraded situation

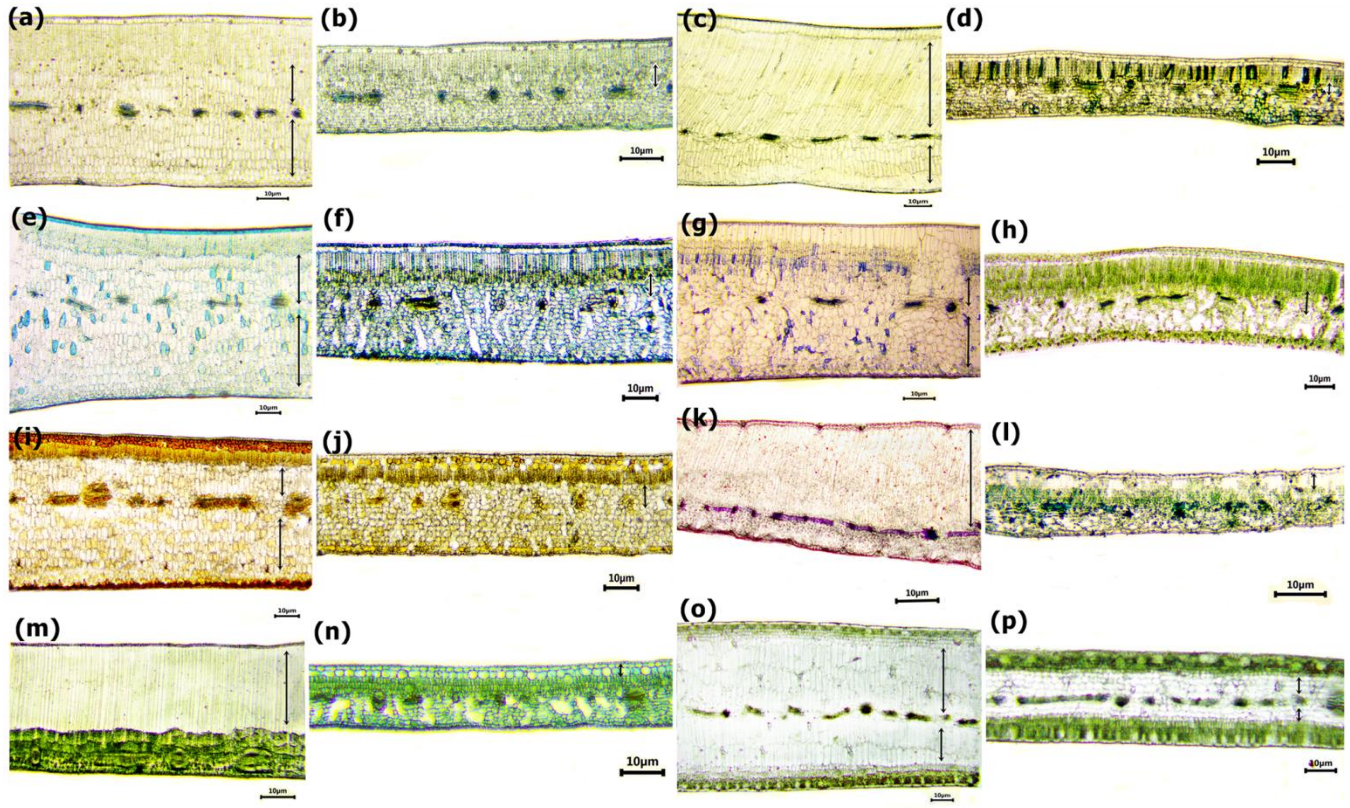

Figure S2: Increase of water storage tissue thickness (WST) as observed from transverse sections of leaves of mangroves and associate species from maximally degraded mangrove forests over control pristine mangrove forests-a distinctly visible eco-

physiological resilience trait. Transverse sections of leaves of (a) *Bruguiera cylindrica*, (c).*Excoecaria agallocha*, (e) *Bruguiera gymnorrhiza* (g) *Finlaysonia obovata* (i) *Ceriops decandra* (k) *Volkameria inermis* (m) *Xylocarpus mekongensis* (o) *Sonneratia caseolaris* are from maximally degraded mangrove sites. Transverse sections of leaves of (b) *Bruguiera cylindrica*, (d) *Excoecaria agallocha*, (f) *Bruguiera gymnorrhiza*, (h) *Finlaysonia obovata*, (j) *Ceriops decandra*, (l) *Volkameria inermis*, (n) *Xylocarpus mekongensis*, (p) *Sonneratia caseolaris* are from pristine (control) mangrove habitats. Location of water storage tissue is indicated with double sided arrows in the pictures.

Table S1:

The measurable variables of the degraded state of mangrove forests to postulate four degradation stages

Name of the mangrove forests	Degradation stages	% Forest Coverage	Ammonia-nitrogen (mg kg^{-1})	Organic carbon (%)	Surface water conductivity (dS m^{-1})	Soil conductivity (dS m^{-1})	Plant available phosphorus (mg kg^{-1})	Phenol oxidase (units)	Sulfide-sulfur (mg kg^{-1})
Bhagabatpur<br>Lakkhipur<br>Durbachoti<br>G-Plot<br>Lothian	Pristine	**80.7 –99.7**	**4.1– 6.4**	**0.9 - 1.3**	**27- 36**	**8 - 19**	**7 - 11**	**0.66 - 1.7**	**0.45 - 1.70**
Birat<br>ShiberGhat<br>L-Plot<br>Dashpur1	Intermediate degradation 1	**52 - 80**	**2.8 - 5.25**	**0.65 - 1**	**27 - 40**	**7.8 - 11.3**	**4.9 - 9.2**	**0.53 -1.1**	**1.26 - 2.8**
Prentice<br>Island X<br>Lakkhipur island<br>Rakkhoshkhali<br>Dashpur 2	Intermediate degradation 2	**21 - 50**	**1.8 - 3.3**	**0.6 - 1**	**26 - 46**	**11 - 18**	**3.2 - 7.7**	**0.3 - 0.7**	**1.8 – 5**
Ramganga<br>Atharogazi	Maximal degradation	**10 - 20**	**1 - 2.3**	**0.5 - 0.9**	**29 - 52**	**15.8 - 23**	**2.1 - 5.3**	**0.1 - 0.54**	**3.8 - 9.9**

Patharprotima<br>Gopalnagar<br>Brajaballavpur									

Table S2: Summary of Canonical Discriminant Functions

Eigenvalues					Wilks' Lambda				
Function	Eigenvalue	% of Variance	Cumulative %	Canonical Correlation	Test of Function(s)	Wilks' Lambda	Chi-square	Df	Sig.
1	26.910[a]	91.2	91.2	.982	1 through 3	.009	584.114	54	.000
2	2.383[a]	8.1	99.2	.839	2 through 3	.242	174.650	34	.000
3	.223[a]	.8	100.0	.427	3	.818	24.754	16	.074
a. First 3 canonical discriminant functions were used in the analysis.									

Table S3: Summary of coefficients ± SE in the best fit mixed effect model

Degradation determinants (Response Variables)	Summary of coefficients ± SE in the best fit mixed effect model<br>Explanatory components (eco-physiological resilience)					
	Constant	WST (Random factor) Variance coefficient ± SE	LT (Fixed factor categorized) Fixed coefficient ± SE	Free Amino Acid (Fixed factor categorized) Fixed coefficient ± SE	Soluble Sugar (Covariate) Fixed coefficient ± SE	ST/LT (Covariate) Fixed coefficient ± SE
Forest coverage	74.029392 ±5.734826	98.825875±76.636812	11.972864±3.996773	10.016651±4.646869	-3.330592±0.629379	-0.022351±0.058071
			6.667690±2.542178	-1.534078±3.582004		
				-5.106429±2.619189		
				0.214273±2.936971		

Ammonia-nitrogen	4.090098±0.416749	0.535225±0.361653	0.430884±0.287572	0.840099±0.333483	-0.089277±0.045476	-0.004045±0.004198
			0.004352±0.182605	-0.128652±0.258882		
				-0.105709±0.188578		
				-0.339836±0.211535		
Organic carbon	0.952487±0.076466	0.001878±0.002994	0.026804±0.062497	0.143880±0.069387	-0.019057±0.010704	-0.000558±0.001000
			0.028826±0.039666	0.007788±0.060966		
				-0.042422±0.041731		
				-0.082053±0.046978		
Tidal water conductivity	39.810213±2.946482	18.820636±19.634747	0.031152±2.203597	-2.930620±2.563656	0.103979±0.347673	-0.051784±0.031988
			-1.225163±1.425709	-2.121885±1.965570		
				-0.468403±1.448975		
				1.184988±1.612724		
Soil conductivity	9.629382±1.644695	2.598160±2.204545	-2.695266±1.304252	2.825924±1.465971	0.594005±0.217882	-0.006902±0.020137
			-1.015533±0.835286	-0.431035±1.231001		
				0.061332±0.866949		
				-1.438203±0.966213		
Plant available phosphorus	8.317281±0.833370	0.988735±0.882638	0.368192±0.646490	1.442375±0.737375	-0.377882±0.105618	0.001856±0.009733
			0.271442±0.418335	0.063678±0.595409		
				-0.082120±0.428329		
				-0.565637±0.475756		
Phenol oxidase activity	0.753078±0.104786	0.008368±0.007196	0.203642±0.083931	0.268588±0.093631	-0.017032±0.014160	0.000345±0.001311
			-0.030875±0.053289	-0.034121±0.080207		
				-0.029562±0.055847		
				-0.097619±0.062433		
Sulfide-sulfur	3.126885±0.566793	0.073184±0.094872	-1.285505±0.469663	-1.187943±0.505243	0.028890±0.081842	0.012419±0.007661
			-0.537517±0.282683	-0.352427±0.471708		
				-0.422380±0.313499		
				0.556755±0.356595		

Table S4: Results of redundancy analysis including F-statistics and p-values derived from permutation tests

Response variables (Y)	Explanatory Variables (Qualitative)	Explanatory Variables (Quantitative)	Inertia			Results of the permutation test		
				Value	%	Permutations	Pseudo F	*p*-value
Degradation determinants	Pristine, Intermediate degradation1, Intermediate degradation2 and Maximal degradation	Eco-physiological resilience A	Total	8.000	100.000	1000	4.063	< **0.0001**
			Constrained	6.420	80.248			
			Unconstrained	1.580	19.752			
		Eco-physiological resilience B	Total	8.000	100.000	1000	3.701	< **0.0001**
			Constrained	6.298	78.729			
			Unconstrained	1.702	21.271			
		Eco-physiological resilience C	Total	8.000	100.000	1000	3.872	< **0.0001**
			Constrained	6.358	79.474			
			Unconstrained	1.642	20.526			

Table S5: Primer sequences used for Quantitative Real Time PCR studies

Name of gene	Primers amplified from the following species	Primers designed from published sequences for the following species	Primer sequences (5'-3') forward/reverse	Amplicon length (bp)	Primer Melting Tm (^{0}C) forward/reverse
18SrRNA	From all the studied species	For all the studied species	ACACCCTGGGAATTGGTTT/ GTATGCGCCAATAAGACCAC	106	64/64
P5CS	*Xylocarpus mekongensis*	*Cicer arietinum*	ATCATACGGGTGCTTCAAGG / CGTCTAGAACTATTACGTGCCG	125	57.3 / 60.3
	Porteresia coarctata	*Oryza sativa*	CAAGTACGAGATTCTCTGATGGG / GAGACCTTCAACACCCACTG	104	60.7 / 59.4
	Sporobolus virginicus	*Aeluropus lagopoides*	CTGCGTAAAGTTGATAGTGCTG / GTCCTGTACTTATGCCAACCTC	103	58.4 / 60.3

	Paspalum vaginatum	*Aeluropus lagopoides*	CTGCGTAAAGTTGATAGTGCTG / GTCCTGTACTTATGCCAACCTC	103	58.4 / 60.3
	Derris trifoliata	*Medicago sativa*	GCCGACCCTTGTAGAGATTTTG / ACAGAGTGCCCCTAATTTTCC	117	60.3 / 57.9
	Dalbergia spinosa	*Medicago sativa*	GCCGACCCTTGTAGAGATTTTG / ACAGAGTGCCCCTAATTTTCC	117	60.3 / 57.9
	Intsia bijuga	*Medicago sativa*	GCCGACCCTTGTAGAGATTTTG / ACAGAGTGCCCCTAATTTTCC	117	60.3 / 57.9
BADH	*Avicennia alba*	*Avicennia marina*	AGTTACGTTGGAGCTTGGTG / GCACCAAGAGTCGAGAAGTAG	140	57.3 / 59.8
	Avicennia marina	*Avicennia marina*	AGTTACGTTGGAGCTTGGTG / GCACCAAGAGTCGAGAAGTAG	140	57.3 / 59.8
	Avicennia officinalis	*Avicennia marina*	AGTTACGTTGGAGCTTGGTG / GCACCAAGAGTCGAGAAGTAG	140	57.3 / 59.8
	Heritiera fomes	*Avicennia marina*	AGTTACGTTGGAGCTTGGTG / GCACCAAGAGTCGAGAAGTAG	140	57.3 / 59.8
	Derris trifoliata	*Medicago sativa*	GATCCCGTCTCGACAGTTATTC / GGCGTCTACATCTTCCTTGG	136	60.3 / 59.4
	Intsia bijuga	*Medicago sativa*	GATCCCGTCTCGACAGTTATTC / GGCGTCTACATCTTCCTTGG	136	60.3 / 59.4
MIPS	*Avicennia alba*	*Avicennia marina*	GATCGTGAAGCCCAAAACTG / TTGCCCATGAAATACCCTCC	149	57.3 / 57.3
	Avicennia marina	*Avicennia marina*	GATCGTGAAGCCCAAAACTG / TTGCCCATGAAATACCCTCC	149	57.3 / 57.3
	Avicennia officinalis	*Avicennia marina*	GATCGTGAAGCCCAAAACTG / TTGCCCATGAAATACCCTCC	149	57.3 / 57.3
	Bruguiera cylindrica	*Avicennia marina*	GATCGTGAAGCCCAAAACTG / TTGCCCATGAAATACCCTCC	149	57.3 / 57.3
	Porteresia coarctata	*Spartina alterniflora*	AGATCTTCATGGGTGGCAAG / CCTCTCCCTCAGTTTTCAGTTG	138	57.3 / 60.3
	Paspalum vaginatum	*Spartina alterniflora*	AGATCTTCATGGGTGGCAAG / CCTCTCCCTCAGTTTTCAGTTG	138	57.3 / 60.3
	Thespesia populnea	*Spartina alterniflora*	AGATCTTCATGGGTGGCAAG / CCTCTCCCTCAGTTTTCAGTTG	138	57.3 / 60.3
F1,6BP	*Porteresia coarctata*	*Porteresia coarctata*	GTTCGTAAAGGCTCTCGTCAG / TCGAGTGGATCAAAGCAGAC	123	59.8 / 57.3
	Myriostachya wightiana,	*Porteresia coarctata*	GTTCGTAAAGGCTCTCGTCAG / TCGAGTGGATCAAAGCAGAC	123	59.8 / 57.3
	Paspalum vaginatum	*Porteresia coarctata*	GTTCGTAAAGGCTCTCGTCAG	123	59.8 / 57.3

			/ TCGAGTGGATCAAAGCAGAC		
	Derris trifoliata	*Porteresia coarctata*	GTTCGTAAAGGCTCTCGTCAG / TCGAGTGGATCAAAGCAGAC	123	59.8 / 57.3
FBA	*Bruguiera cylindrica*	*Bruguiera gymnorhiza*	AAGCCAGAAAGCCCACATAG / TCCATTGCCAGAATACCACG	143	57.3 / 57.3
	Rhizophora mucronata	*Bruguiera gymnorhiza*	AAGCCAGAAAGCCCACATAG / TCCATTGCCAGAATACCACG	143	57.3 / 57.3
	Porteresia coarctata	*Bruguiera gymnorrhiza*	AAGCCAGAAAGCCCACATAG / TCCATTGCCAGAATACCACG	143	57.3 / 57.3
	Sporobolus virginicus	*Bruguiera gymnorrhiza*	AAGCCAGAAAGCCCACATAG / TCCATTGCCAGAATACCACG	143	57.3 / 57.3
	Finlaysonia obovata	*Bruguiera gymnorrhiza*	AAGCCAGAAAGCCCACATAG / TCCATTGCCAGAATACCACG	143	57.3 / 57.3
F2,6BP	*Bruguiera cylindrica*	*Bruguiera gymnorhiza*	AATACACATCTCACACCTCGC / ACCGCCAATTCTACCTCTAAC	81	57.9 / 57.9
	Rhizophora mucronata	*Bruguiera gymnorhiza*	AATACACATCTCACACCTCGC / ACCGCCAATTCTACCTCTAAC	81	57.9 / 57.9
	Porteresia coarctata	*Bruguiera gymnorhiza*	AATACACATCTCACACCTCGC / ACCGCCAATTCTACCTCTAAC	81	57.9 / 57.9
	Sporobolus virginicus	*Bruguiera gymnorhiza*	AATACACATCTCACACCTCGC / ACCGCCAATTCTACCTCTAAC	81	57.9 / 57.9
	Intsia bijuga	*Bruguiera gymnorhiza*	AATACACATCTCACACCTCGC / ACCGCCAATTCTACCTCTAAC	81	57.9 / 57.9
SUS	*Sporobolus virginicus*	*Saccharum officinarum*	GACTGGTGTATACGGATTCTGG / ATCGAAGGACAATGGAACCG	127	60.3 / 57.3
	Derris trifoliata	*Pisum sativum 1*	GTATGAGAGTCACACTGCCTTC / TCAATCTACGCTCGGTTTCG	146	60.3 / 57.3
	Acanthus ilicifolius	*Pisum sativum 1*	GTATGAGAGTCACACTGCCTTC / TCAATCTACGCTCGGTTTCG	146	60.3 / 57.3
	Excoecaria agallocha	*Glycine max 1*	CCTGATCGAGACCTACAAGTTG / CTGTCAAACCAAAAGCCTCG	149	60.3 / 57.3

Table S6A: The data on measurable variables of degradation of mangrove forests used for CDFA, RDA, Bayesian linear regression analysis and Linear mixed effects models

Name of forests	Types of forests	% Forest Coverage	Ammonia-nitrogen (mg kg^{-1})	Organic carbon (%)	Surface water conductivity (dS m^{-1})	Soil conductivity (dS m^{-1})	Plant available phosphorus (mg kg^{-1})	Phenol oxidase activity	Sulfide-sulfur (mg kg-

								**(units)**	**[1])**
Bhagabatpur	Pristine	**98.189**	**5.795**	**1.315**	36.130	8.601	**10.767**	**1.176**	**0.671**
Lakkhipur	Pristine	**94.998**	**6.285**	**1.051**	29.209	8.500	**10.690**	**1.290**	**1.190**
Durbachoti	Pristine	**92.140**	**5.503**	**1.151**	27.228	8.805	**9.674**	**1.139**	**0.669**
G-Plot	Pristine	**87.219**	**5.502**	**1.019**	32.466	8.598	**9.756**	**1.515**	**1.388**
Lothian	Pristine	**83.424**	**5.055**	**1.241**	35.282	8.499	**10.009**	**1.228**	**1.563**
Lakkhipur	Pristine	**98.892**	4.402	1.146	35.510	14.545	8.840	1.389	0.667
Lakkhipur	Pristine	**99.755**	4.277	1.127	30.250	16.197	7.347	0.768	0.676
Lakkhipur	Pristine	**95.920**	4.126	1.100	30.250	13.901	7.263	1.162	1.184
Lakkhipur	Pristine	**96.788**	5.506	1.015	31.290	14.004	9.756	1.513	0.545
Lakkhipur	Pristine	**92.646**	5.394	0.985	30.290	14.076	10.418	1.130	1.327
Durbachoti	Pristine	**97.891**	4.730	0.952	31.250	9.485	9.925	0.829	0.750
Durbachoti	Pristine	**94.788**	4.441	0.913	33.390	9.338	7.587	1.622	1.244
Durbachoti	Pristine	**92.315**	5.506	1.157	34.357	8.803	9.672	1.130	0.513
Durbachoti	Pristine	**93.711**	4.929	1.067	35.300	9.502	8.827	1.429	1.034
Durbachoti	Pristine	**80.754**	4.742	0.977	32.400	10.296	8.068	1.590	1.569
G-Plot	Pristine	**92.646**	4.440	0.956	32.400	8.930	7.027	1.681	0.498
G-Plot	Pristine	**90.588**	6.422	1.132	33.570	9.800	10.769	1.196	1.704
G-Plot	Pristine	**93.187**	5.506	0.993	31.240	9.304	9.334	1.276	0.451
G-Plot	Pristine	**91.588**	6.285	1.029	33.560	7.997	9.756	1.334	1.394
G-Plot	Pristine	**92.187**	**5.603**	**0.926**	34.520	8.619	**9.756**	**1.241**	**0.677**
Lothian	Pristine	**86.814**	**6.123**	**1.241**	32.541	17.950	**9.543**	**0.738**	**1.253**
Lothian	Pristine	**89.688**	**5.874**	**0.987**	33.877	17.798	**9.017**	**0.822**	**0.741**
Lothian	Pristine	**85.155**	**5.291**	**0.962**	31.864	18.903	**8.552**	**0.659**	**0.669**
Lothian	Pristine	**83.391**	**4.977**	**0.956**	33.566	16.700	**9.034**	**0.983**	**1.148**
Lothian	Pristine	**87.833**	**5.906**	**1.082**	33.611	18.397	**10.055**	**0.798**	**1.111**
Birat	Intermediate degradation 1	**71.515**	**4.429**	**0.982**	26.746	9.852	**6.822**	**0.646**	**1.882**
ShiberGhat	Intermediate degradation 1	**65.520**	**4.386**	**0.875**	34.000	9.803	**7.724**	**0.984**	**2.071**
L-Plot	Intermediate degradation 1	**61.916**	**4.909**	**0.873**	30.928	9.304	**7.495**	**0.897**	**2.154**
Dashpur1	Intermediate degradation 1	**53.147**	**5.028**	**0.965**	34.316	10.003	**7.057**	**0.607**	**1.977**
Dashpur1	Intermediate degradation 1	**71.862**	5.030	0.970	27.250	10.298	7.055	0.609	1.636
ShiberGhat	Intermediate degradation 1	**73.583**	3.830	0.766	36.980	10.001	6.477	0.943	2.151
ShiberGhat	Intermediate	**69.099**	3.313	0.741	32.850	10.896	6.239	0.614	1.265

	degradation 1								
ShiberGhat	Intermediate degradation 1	**62.383**	2.813	0.722	36.540	10.004	5.055	0.530	2.075
ShiberGhat	Intermediate degradation 1	**64.407**	4.900	0.872	39.290	10.001	7.496	0.899	1.650
ShiberGhat	Intermediate degradation 1	**64.407**	3.599	0.766	35.240	9.103	6.590	0.710	2.754
L-Plot	Intermediate degradation 1	**69.199**	3.448	0.751	37.450	9.373	6.350	1.131	2.529
L-Plot	Intermediate degradation 1	**62.955**	3.066	0.711	35.250	9.796	5.912	0.883	1.644
L-Plot	Intermediate degradation 1	**71.099**	4.390	0.876	36.630	9.296	7.730	0.987	1.950
L-Plot	Intermediate degradation 1	**61.256**	4.263	0.872	37.850	9.299	7.035	0.761	2.034
L-Plot	Intermediate degradation 1	**62.383**	**4.051**	**0.822**	27.385	9.101	**6.139**	**0.713**	**1.569**
Dashpur1	Intermediate degradation 1	**58.256**	**3.473**	**0.799**	38.540	10.827	**5.379**	**0.578**	**2.066**
Dashpur1	Intermediate degradation 1	**65.108**	**3.423**	**1.031**	31.520	11.301	**8.827**	**0.937**	**1.531**
Dashpur1	Intermediate degradation 1	**66.199**	**3.900**	**0.989**	36.580	11.099	**9.165**	**0.598**	**2.096**
Dashpur1	Intermediate degradation 1	**65.955**	**3.599**	**0.978**	29.520	10.904	**8.574**	**0.596**	**1.978**
Dashpur1	Intermediate degradation 1	**54.612**	**3.448**	**0.959**	38.240	10.004	**8.152**	**0.714**	**1.514**
Birat	Intermediate degradation 1	**51.984**	**4.929**	**1.076**	39.250	8.576	**4.944**	**0.685**	**2.156**
Birat	Intermediate degradation 1	**52.844**	**4.942**	**1.015**	37.250	9.535	**5.282**	**0.766**	**1.701**
Birat	Intermediate degradation 1	**57.632**	**4.440**	**1.001**	35.950	9.351	**4.944**	**0.735**	**1.942**
Birat	Intermediate degradation 1	**54.844**	**3.486**	**0.976**	34.940	9.429	**5.620**	**0.936**	**1.864**
Birat	Intermediate degradation 1	**56.711**	**4.477**	**0.652**	39.740	8.559	**5.620**	**0.898**	**2.722**
ShiberGhat	Intermediate degradation 1	**55.906**	**5.255**	**0.699**	39.180	7.798	**4.944**	**0.596**	**2.725**

ShiberGhat	Intermediate degradation 1	**74.331**	**3.688**	**0.733**	36.722	9.549	**6.598**	**0.599**	**1.944**
ShiberGhat	Intermediate degradation 1	**64.122**	**4.271**	**0.719**	38.951	8.580	**6.887**	**0.672**	**1.948**
Dashpur1	Intermediate degradation 1	**79.980**	**4.209**	**0.854**	38.544	10.649	**7.512**	**0.833**	**1.877**
Dashpur1	Intermediate degradation 1	**72.184**	**3.922**	**0.811**	39.088	9.741	**7.788**	**0.588**	**2.109**
Dashpur1	Intermediate degradation 1	**73.430**	**3.870**	0.751	39.512	9.157	**8.033**	**0.782**	**1.855**
Prentice	Intermediate degradation 2	**48.772**	**2.516**	**0.720**	26.416	16.575	**5.616**	**0.579**	**3.804**
Island X	Intermediate degradation 2	**42.841**	**2.798**	**0.733**	36.000	17.504	**4.614**	**0.587**	**2.487**
Lakkhipur island	Intermediate degradation 2	**40.257**	**3.373**	**0.758**	34.300	17.302	**5.861**	**0.515**	**2.569**
Rakkhoshkhali	Intermediate degradation 2	**37.218**	**2.929**	**0.758**	33.340	15.595	**5.146**	**0.598**	**2.447**
Dashpur 2	Intermediate degradation 2	**28.599**	**2.864**	**0.726**	31.340	15.898	**5.873**	**0.595**	**3.273**
Island X	Intermediate degradation 2	**49.056**	2.294	0.706	40.520	12.083	4.295	0.566	3.449
Island X	Intermediate degradation 2	**50.108**	2.055	0.675	41.620	12.395	3.788	0.535	2.559
Island X	Intermediate degradation 2	**47.153**	1.779	0.654	43.250	12.200	3.535	0.436	2.806
Island X	Intermediate degradation 2	**40.298**	2.921	0.751	44.250	11.944	5.149	0.590	2.809
Island X	Intermediate degradation 2	**42.714**	2.821	**0.673**	42.560	11.795	4.281	0.462	3.274
Lakkhipur island	Intermediate degradation 2	**42.714**	**2.231**	**0.652**	42.960	11.406	**3.973**	**0.593**	**3.518**
Lakkhipur island	Intermediate degradation 2	**45.176**	**2.034**	**0.617**	42.620	11.900	**3.437**	**0.686**	**2.980**
Lakkhipur island	Intermediate degradation 2	**40.632**	**2.520**	**0.710**	45.620	11.005	**5.620**	**0.578**	**4.385**
Lakkhipur island	Intermediate degradation 2	**48.108**	**2.168**	**0.731**	43.260	10.901	**4.620**	**0.428**	**4.956**
Lakkhipur	Intermediate	**40.230**	**2.055**	**0.700**	43.680	11.819	**3.944**	**0.387**	**3.528**

island	degradation 2								
Rakkhoshkhali	Intermediate degradation 2	**40.298**	**1.767**	**0.683**	43.670	13.499	**3.272**	**0.631**	**2.317**
Rakkhoshkhali	Intermediate degradation 2	**42.230**	**2.868**	**0.728**	44.740	13.695	**5.876**	**0.591**	**3.321**
Rakkhoshkhali	Intermediate degradation 2	**38.243**	**2.557**	**0.684**	44.570	13.400	**4.813**	**0.530**	**3.715**
Rakkhoshkhali	Intermediate degradation 2	**45.431**	**2.193**	**0.675**	43.560	13.999	**4.083**	**0.428**	**4.245**
Rakkhoshkhali	Intermediate degradation 2	**36.572**	**1.830**	**0.653**	42.590	12.902	**3.547**	**0.334**	**4.563**
Dashpur 2	Intermediate degradation 2	**36.982**	**2.495**	**0.970**	43.210	16.375	**7.730**	**0.680**	**3.942**
Dashpur 2	Intermediate degradation 2	**39.572**	**1.804**	**1.008**	45.240	16.396	**7.055**	**0.505**	**4.215**
Dashpur 2	Intermediate degradation 2	**26.088**	**2.269**	**1.002**	41.240	16.105	**7.477**	**0.640**	**4.213**
Dashpur 2	Intermediate degradation 2	**29.088**	**2.532**	**0.963**	42.240	16.402	**7.477**	**0.395**	**4.653**
Dashpur 2	Intermediate degradation 2	**27.332**	**3.377**	**0.750**	42.410	16.596	**5.863**	**0.512**	**1.983**
Prentice	Intermediate degradation 2	**21.411**	**3.095**	**0.699**	42.650	12.140	**5.224**	**0.399**	**2.566**
Prentice	Intermediate degradation 2	**24.107**	**3.021**	**0.666**	43.520	14.040	**4.290**	**0.340**	**2.127**
Prentice	Intermediate degradation 2	**26.421**	**2.379**	**0.652**	45.840	16.860	**3.984**	**0.307**	**2.482**
Prentice	Intermediate degradation 2	**46.227**	**2.796**	**0.733**	43.630	12.970	**4.620**	**0.585**	**1.866**
Prentice	Intermediate degradation 2	**45.109**	**2.294**	**0.706**	40.520	13.260	**4.295**	**0.566**	**2.449**
Island X	Intermediate degradation 2	**41.066**	**2.055**	**0.675**	41.620	17.560	**3.788**	**0.535**	**2.559**
Island X	Intermediate degradation 2	**39.187**	**1.779**	**0.654**	43.250	12.750	**3.535**	**0.436**	**3.806**
Island X	Intermediate degradation 2	**21.547**	**2.921**	**0.751**	44.250	12.830	**5.149**	**0.590**	**2.409**
Island X	Intermediate degradation 2	**29.655**	**2.821**	**0.673**	42.560	12.140	**4.281**	**0.462**	**3.274**

Island X	Intermediate degradation 2	**42.384**	**2.231**	**0.652**	42.960	13.060	**3.973**	**0.393**	**3.518**
Rakkhoshkhali	Intermediate degradation 2	**41.887**	**2.034**	**0.617**	42.620	12.880	**3.437**	**0.386**	**2.980**
Rakkhoshkhali	Intermediate degradation 2	**36.138**	**2.520**	**0.710**	45.620	12.000	**5.620**	**0.578**	**4.385**
Rakkhoshkhali	Intermediate degradation 2	**48.289**	**2.168**	**0.731**	43.260	14.040	**4.620**	**0.428**	**4.956**
Rakkhoshkhali	Intermediate degradation 2	**41.186**	**2.055**	**0.700**	43.680	11.565	**3.944**	**0.387**	**3.528**
Rakkhoshkhali	Intermediate degradation 2	**35.345**	**1.767**	**0.683**	43.670	12.096	**3.272**	**0.331**	**4.317**
Dashpur 2	Intermediate degradation 2	**28.332**	**2.868**	**0.728**	44.740	12.388	**5.876**	**0.591**	**3.321**
Dashpur 2	Intermediate degradation 2	**36.876**	**2.557**	**0.684**	44.570	12.171	**4.813**	**0.530**	**4.215**
Dashpur 2	Intermediate degradation 2	**47.354**	**2.193**	**0.675**	43.560	13.062	**4.083**	**0.428**	**4.245**
Dashpur 2	Intermediate degradation 2	**49.134**	**1.830**	**0.653**	42.590	13.810	**3.547**	**0.334**	**4.563**
Dashpur2	Intermediate degradation 2	**44.545**	**2.495**	**0.970**	43.210	13.420	**7.730**	**0.380**	**3.942**
Prentice	Intermediate degradation 2	**30.566**	**1.804**	**1.008**	45.240	12.770	**7.055**	**0.305**	**4.215**
Prentice	Intermediate degradation 2	**48.345**	**2.269**	**1.002**	41.240	12.630	**7.477**	**0.340**	**4.213**
Prentice	Intermediate degradation 2	**42.159**	**2.532**	**0.963**	42.240	11.580	**7.477**	**0.695**	**4.653**
Ramganga	Maximal degradation	**17.613**	**1.897**	**0.592**	34.774	16.023	**3.289**	**0.180**	**8.091**
Atharogazi	Maximal degradation	**17.276**	**1.616**	**0.611**	33.314	15.800	**4.360**	**0.537**	**5.939**
Patharprotima	Maximal degradation	**12.793**	**2.015**	**0.614**	29.037	15.896	**3.436**	**0.335**	**6.863**
Gopalnagar	Maximal degradation	**11.642**	**2.108**	**0.609**	30.698	16.601	**4.532**	**0.342**	**3.802**
Brajaballavpur	Maximal degradation	**11.016**	**2.002**	**0.692**	32.840	15.796	**3.342**	**0.240**	**8.853**
Atharogazi	Maximal	**17.406**	**1.505**	**0.583**	45.650	17.787	**3.620**	**0.122**	**9.861**

	degradation								
Atharogazi	Maximal degradation	**15.456**	**1.453**	**0.570**	48.120	17.998	**2.944**	**0.188**	**6.117**
Atharogazi	Maximal degradation	**19.976**	**1.149**	**0.510**	47.520	18.097	**3.775**	**0.127**	**8.852**
Atharogazi	Maximal degradation	**12.448**	**2.015**	**0.612**	47.560	17.836	**3.437**	**0.334**	**4.178**
Atharogazi	Maximal degradation	**10.032**	**1.804**	**0.572**	48.260	17.216	**3.362**	**0.271**	**3.806**
Patharprotima	Maximal degradation	**17.269**	**1.669**	**0.570**	48.360	21.798	**4.937**	**0.166**	**6.322**
Patharprotima	Maximal degradation	**18.234**	**1.232**	**0.561**	47.560	20.701	**2.532**	**0.430**	**8.093**
Patharprotima	Maximal degradation	**16.327**	**2.023**	**0.699**	48.250	22.996	**3.347**	**0.238**	**5.938**
Patharprotima	Maximal degradation	**12.232**	**1.390**	**0.606**	47.280	20.598	**3.162**	**0.232**	**5.310**
Patharprotima	Maximal degradation	**15.287**	**1.390**	**0.531**	48.240	22.896	**2.698**	**0.197**	**6.861**
Gopalnagar	Maximal degradation	**12.448**	**1.016**	**0.514**	49.250	20.321	**2.385**	**0.185**	**6.450**
Gopalnagar	Maximal degradation	**14.976**	**2.106**	**0.603**	50.250	20.684	**4.535**	**0.340**	**5.291**
Gopalnagar	Maximal degradation	**10.955**	**1.980**	**0.542**	52.120	19.999	**3.598**	**0.229**	**6.171**
Gopalnagar	Maximal degradation	**10.096**	**1.608**	**0.521**	50.170	20.297	**3.216**	**0.215**	**6.194**
Gopalnagar	Maximal degradation	**16.276**	**1.223**	**0.482**	49.142	20.304	**2.134**	**0.205**	**8.405**
Brajaballavpur	Maximal degradation	**14.562**	**2.357**	**0.540**	46.268	19.126	**5.286**	**0.173**	**6.020**
Brajaballavpur	Maximal degradation	**13.358**	**1.892**	**0.645**	48.650	19.800	**3.724**	**0.452**	**7.183**
Brajaballavpur	Maximal degradation	**10.658**	**2.081**	**0.683**	47.250	18.897	**3.290**	**0.276**	**9.325**
Brajaballavpur	Maximal degradation	**17.577**	**1.980**	**0.704**	48.650	19.505	**3.984**	**0.196**	**8.446**
Brajaballavpur	Maximal degradation	**11.786**	**1.616**	**0.750**	47.250	18.304	**4.026**	**0.256**	**5.321**

Ramganga	Maximal degradation	**10.476**	**1.441**	**0.904**	45.680	19.020	**2.758**	**0.154**	**6.853**
Ramganga	Maximal degradation	**10.658**	**1.503**	**0.882**	47.240	18.310	**3.437**	**0.219**	**6.765**
Ramganga	Maximal degradation	**16.345**	**1.993**	**0.861**	48.250	18.640	**4.932**	**0.533**	**6.986**
Ramganga	Maximal degradation	**15.466**	**1.833**	**0.533**	46.386	17.550	**2.544**	**0.218**	**7.311**
Ramganga	Maximal degradation	**17.327**	**1.921**	**0.499**	47.399	16.990	**3.167**	**0.289**	**6.735**
Ramganga	Maximal degradation	**17.368**	**1.344**	**0.585**	49.332	17.830	**2.988**	**0.207**	**7.188**

Table S6B: The data on quantifiable eco-physiological resilience of mangrove forests used for CDFA, RDA, Bayesian linear regression analysis and Linear mixed effects models

Name of forests	Degradation stage	Plant species	Ecophysiological resilience	Values
Durbachoti	Pristine	*Avicennia alba*	Glycine betaine in (mg/g of DW)	14.160
Durbachoti				13.880
Durbachoti				12.411
G-Plot				19.735
G-Plot				14.715
G-Plot				18.401
Birat	Intermediate degradation 1			9.469
ShiberGhat				8.247
L-Plot				7.558
L-Plot				7.366
Prentice	Intermediate degradation 2			6.818
Island X				5.973
Lakkhipur island				5.003
Rakkhoshkhali				6.114
Island X				5.168
Rakkhoshkhali				5.088
Rakkhoshkhali				5.051
Brajaballavpur	Maximal degradation			4.440
Patharprotima				3.497
Gopalnagar				4.657
Gopalnagar				2.265
Gopalnagar				2.301
Gopalnagar				3.667
Bhagabatpur	Pristine		Unit of SOD / mg of protein	5.609
Birat	Intermediate degradation 1			6.675
ShiberGhat				6.880
Prentice	Intermediate degradation 2			8.013
Island X				7.667
Ramganga	Maximal degradation			5.317
Bhagabatpur	Pristine		Total Na+/K+	1.463
Birat	Intermediate degradation 1			2.088
Prentice	Intermediate degradation 2			1.593
Island X				3.093
Ramganga	Maximal degradation			2.318
Atharogazi				1.650
Bhagabatpur	Pristine		Total chlorophyll (mg/100 g of FW)	89.342
Lakkhipur				101.459
Birat	Intermediate degradation 1			80.457
Lakkhipur island	Intermediate degradation 2			62.565
Rakkhoshkhali				69.654
Ramganga	Maximal			16.591
Atharogazi				25.995
Bhagabatpur	Pristine		PEPC activity/RuBPC activity	1.921

Durbachoti			(µmol/minute/µg of protein)	1.893
Birat	Intermediate degradation 1			1.524
Dashpur1				1.526
Prentice	Intermediate degradation 2			1.432
Dashpur 2				1.433
Rakkhoshkhali				1.433
Rakkhoshkhali				1.433
Ramganga	Maximal			1.307
Ramganga				1.305
Lothian	Pristine		*BADH*(ΔCQ)	14.3
Lothian				14.22
Lothian				9.89
Durbachoti				16.18
Durbachoti				15.87
ShiberGhat	Intermediate degradation 1			8.36
ShiberGhat				8.3
ShiberGhat				8.23
Dashpur1				8.12
Dashpur1				7.91
Dashpur1				7.82
Birat				8.42
Birat				8.38
Rakkhoshkhali	Intermediate degradation 2			2.33
Rakkhoshkhali				2.31
Dashpur 2				2.3
Dashpur 2				2.27
Dashpur 2				2.27
Dashpur 2				2.24
Dashpur2				2.22
Prentice				2.15
Prentice				2.13
Prentice				2.11
Brajaballavpur	Maximal degradation			0.34
Ramganga				0.34
Ramganga				0.31
Ramganga				0.26
Ramganga				-0.71
G-Plot	Pristine		*MIPS*(ΔCQ)	2.49
Lothian				1.51
Lothian				2.78
L-Plot	Intermediate degradation 1			5.91
L-Plot				5.92
L-Plot				5.95
Lakkhipur island	Intermediate degradation 2			12.73
Lakkhipur island				12.75
Lakkhipur island				12.84
Lakkhipur island				13.49
Atharogazi	Maximal degradation			15.54
Atharogazi				15.57
Patharprotima				15.58

Lothian	Pristine	*Avicennia marina*	Glycine betaine in (mg/g of DW)	18.084
Lothian				17.203
Lothian				16.434
Dashpur1	Intermediate degradation 1			9.879
ShiberGhat				8.478
ShiberGhat				8.581
ShiberGhat				7.467
L-Plot				7.129
L-Plot				7.005
L-Plot				7.000
Island X	Intermediate degradation 2			6.254
Island X				6.998
Island X				6.912
Island X				6.834
Lakkhipur island				6.892
Lakkhipur island				6.332
Dashpur 2				5.556
Dashpur 2				5.299
Dashpur 2				5.688
Dashpur 2				6.568
Brajaballavpur	Maximal degradation			4.788
Brajaballavpur				4.228
Brajaballavpur				2.014
Ramganga				1.997
Ramganga				3.341
Ramganga				2.937
Ramganga				3.125
Ramganga				3.108
Ramganga				4.954
Lakkhipur	Pristine		Unit of SOD / mg of protein	7.284
ShiberGhat	Intermediate degradation 1			5.566
Dashpur1				7.031
Lakkhipur island	Intermediate degradation 2			5.721
Rakkhoshkhali				8.921
Patharprotima	Maximal degradation			4.387
Lakkhipur	Pristine		Total Na+/K+	0.870
ShiberGhat	Intermediate degradation 1			1.665
Lakkhipur island	Intermediate degradation 2			1.955
Rakkhoshkhali				3.058
Gopalnagar	Maximal degradation			1.710
Brajaballavpur				2.155
Durbachoti	Pristine		Total chlorophyll (mg/100 g of FW)	58.098
G-Plot				78.216
ShiberGhat	Intermediate degradation 1			92.567
Dashpur 2	Intermediate degradation 2			38.763
Island X				40.083
Island X				43.864

Island X				54.540
Patharprotima	Maximal degradation			32.873
Lakkhipur	Pristine		PEPC activity/RuBPC activity (µmol/minute/µg of protein	1.629
G-Plot				1.632
ShiberGhat	Intermediate degradation 1			0.525
Birat				0.520
Island X	Intermediate degradation 2			0.017
Dashpur 2				0.018
Rakkhoshkhali				0.017
Rakkhoshkhali				0.018
Atharogazi	Maximal degradation			0.000
Patharprotima				0.000
G-Plot	Pristine		*BADH*(ΔCQ)	15.53
G-Plot				14.77
G-Plot				14.76
G-Plot				14.76
Lothian				9.77
Dashpur1	Intermediate degradation 1			8.51
Dashpur1				8.49
Birat				8.43
Birat				8.43
Birat				8.43
Prentice	Intermediate degradation 2			2.83
Prentice				2.78
Island X				2.7
Island X				2.67
Island X				2.65
Island X				2.65
Island X				2.61
Rakkhoshkhali				2.56
Rakkhoshkhali				2.45
Rakkhoshkhali				2.35
Brajaballavpur	Maximal degradation			0.49
Brajaballavpur				0.43
Brajaballavpur				0.42
Brajaballavpur				0.38
Ramganga				-0.9
G-Plot	Pristine		*MIPS*(ΔCQ)	2.11
Lothian				2.29
Lakkhipur				2.33
L-Plot	Intermediate degradation 1			5.95
Dashpur1				6.03
Dashpur1				6.09
Island X	Intermediate degradation 2			10.56
Island X				10.74
Island X				9.12
Island X				8.04
Island X				8.04
Rakkhoshkhali				9.02
Patharprotima	Maximal degradation			15.65
Patharprotima				17.83
Patharprotima				17.95
Patharprotima				17.97

G-Plot	Pristine	*Avicennia officinalis*	Glycine betaine in (mg/g of DW)	10.893
G-Plot				15.304
Lothian				13.238
Lothian				16.962
ShiberGhat	Intermediate degradation 1			7.817
ShiberGhat				7.268
Birat				8.365
Birat				8.123
Birat				7.976
Birat				7.297
Dashpur 2	Intermediate degradation 2			5.230
Prentice				5.006
Prentice				5.347
Prentice				6.103
Island X				6.081
Island X				6.076
Island X				6.854
Island X				6.748
Rakkhoshkhali				6.099
Dashpur 2				5.183
Gopalnagar	Maximal degradation			3.450
Brajaballavpur				2.413
Brajaballavpur				4.394
G-Plot	Pristine		Unit of SOD / mg of protein	2.479
L-Plot	Intermediate degradation 1			5.863
Island X	Intermediate degradation 2			8.325
Dashpur 2				7.548
Patharprotima	Maximal degradation			8.969
Durbachoti	Pristine		Total Na+/K+	1.120
L-Plot	Intermediate degradation 1			1.321
Dashpur 2	Intermediate degradation 2			3.724
Island X				2.490
Atharogazi	Maximal degradation			3.157
Lothian	Pristine		Total chlorophyll (mg/100 g of FW)	89.524
L-Plot	Intermediate degradation 1			79.767
Island X	Intermediate degradation 2			55.993
Island X				47.378
Gopalnagar	Maximal degradation			45.610
Brajaballavpur				30.604
Durbachoti	Pristine		PEPC activity/RuBPC activity (µmol/minute/µg of protein	1.234
G-Plot				1.227
L-Plot	Intermediate degradation 1			0.439
Birat				0.438
Lakkhipur island	Intermediate degradation 2			0.053
Dashpur 2				0.052
Dashpur 2				0.054
Ramganga	Maximal degradation			0.000
Ramganga				0.000

Ramganga				0.000
G-Plot	Pristine		*BADH*(ΔCQ)	14.56
Lothian				14.44
Durbachoti				15.76
Durbachoti				16.23
Durbachoti				16.21
L-Plot	Intermediate degradation 1			8.67
L-Plot				8.64
Dashpur1				8.61
Dashpur1				8.56
Dashpur1				8.55
Dashpur 2	Intermediate degradation 2			4.76
Dashpur 2				3.44
Dashpur 2				3.41
Prentice				3.25
Prentice				3.17
Prentice				3.05
Gopalnagar	Maximal degradation			0.6
Gopalnagar				0.56
Gopalnagar				0.55
Ramganga				-1.1
Lakkhipur	Pristine		*MIPS*(ΔCQ)	2.74
Lakkhipur				2.76
Lakkhipur				2.87
Dashpur1	Intermediate degradation 1			6.13
Dashpur1				6.23
Dashpur1				6.28
Prentice	Intermediate degradation 2			10.47
Prentice				10.32
Prentice				11.16
Prentice				11.42
Prentice				10.24
Gopalnagar	Maximal degradation			14.09
Gopalnagar				13.67
Brajaballavpur				13.87
Ramganga				15.08
Ramganga				15.68
Lakkhipur	Pristine	*Rhizophora mucronata*	Leaf thickness (LT) µm	46.090
Lakkhipur				48.880
Lakkhipur				43.680
Lakkhipur				46.280
Birat	Intermediate degradation 1			77.850
Birat				76.360
Rakkhoshkhali	Intermediate degradation 2			179.320
Rakkhoshkhali				185.650
Dashpur 2				165.360
Brajaballavpur	Maximal degradation			200.690
Ramganga				214.340
Lakkhipur	Pristine		Water storage tissue (WST) µm	20.680
Lakkhipur				21.310
Lakkhipur				20.740
Lakkhipur				21.540
Birat	Intermediate			35.340

Birat	degradation 1			29.055
Rakkhoshkhali	Intermediate degradation 2			127.420
Rakkhoshkhali				130.240
Dashpur 2				108.500
Brajaballavpur	Maximal degradation			177.240
Ramganga				165.421
Lakkhipur	Pristine		Palisade tissue (PT) µm	18.090
Lakkhipur				18.250
Lakkhipur				13.680
Lakkhipur				17.050
Birat	Intermediate degradation 1			22.450
Birat				22.360
Rakkhoshkhali	Intermediate degradation 2			42.580
Rakkhoshkhali				46.360
Dashpur 2				47.320
Brajaballavpur	Maximal degradation			17.450
Ramganga				38.410
Lakkhipur	Pristine		Spongy tissue (ST) µm	6.320
Lakkhipur				7.320
Lakkhipur				8.260
Lakkhipur				6.690
Birat	Intermediate degradation 1			18.060
Birat				23.450
Rakkhoshkhali	Intermediate degradation 2			7.320
Rakkhoshkhali				7.050
Dashpur 2				7.540
Brajaballavpur	Maximal degradation			5.000
Ramganga				11.930
Lakkhipur	Pristine		PT/ST	2.862
Lakkhipur				2.493
Lakkhipur				1.656
Lakkhipur				2.549
Birat	Intermediate degradation 1			1.243
Birat				0.954
Rakkhoshkhali	Intermediate degradation 2			5.817
Rakkhoshkhali				6.576
Dashpur 2				6.276
Brajaballavpur	Maximal degradation			3.490
Ramganga				3.220
Lakkhipur	Pristine		PT/LT	0.392
Lakkhipur				0.373
Lakkhipur				0.313
Lakkhipur				0.368
Birat	Intermediate degradation 1			0.288
Birat				0.293
Rakkhoshkhali	Intermediate degradation 2			0.237
Rakkhoshkhali				0.250
Dashpur 2				0.286
Brajaballavpur	Maximal degradation			0.087
Ramganga				0.179
Lakkhipur	Pristine		ST/LT	0.137
Lakkhipur				0.150
Lakkhipur				0.189

Lakkhipur				0.145
Birat	Intermediate degradation 1			0.232
Birat				0.307
Rakkhoshkhali	Intermediate degradation 2			0.041
Rakkhoshkhali				0.038
Dashpur 2				0.046
Brajaballavpur	Maximal degradation			0.025
Ramganga				0.056
Lakkhipur	Pristine		WST/LT	0.449
Lakkhipur				0.436
Lakkhipur				0.475
Lakkhipur				0.465
Birat	Intermediate degradation 1			0.454
Birat				0.381
Rakkhoshkhali	Intermediate degradation 2			0.711
Rakkhoshkhali				0.702
Dashpur 2				0.656
Brajaballavpur	Maximal degradation			0.883
Ramganga				0.772
Lakkhipur	Pristine		*FBA*(ΔCQ)	4.34
Lakkhipur				4.53
Lothian				4.16
Lothian				4.14
G-Plot				5.36
L-Plot	Intermediate degradation 1			7.53
L-Plot				7.54
L-Plot				7.61
Birat				9.57
Birat				8.38
ShiberGhat				8.26
Island X	Intermediate degradation 2			11.81
Rakkhoshkhali				11.84
Rakkhoshkhali				11.98
Rakkhoshkhali				10.48
Prentice				10.78
Prentice				11.54
Island X				10.69
Lakkhipur island				11.24
Lakkhipur island				11.27
Patharprotima	Maximal degradation			13.97
Patharprotima				14.06
Patharprotima				14.73
Patharprotima				14.76
Patharprotima				14.81
Bhagabatpur	Pristine		*F26BP*(ΔCQ)	1.67
Lakkhipur				1.87
Lakkhipur				1.68
Durbachoti				1.88
Durbachoti				1.92
ShiberGhat	Intermediate degradation 1			3.98
ShiberGhat				3.94
L-Plot				4.48

L-Plot				4.24
Birat				3.68
Birat				4.42
Dashpur 2	Intermediate degradation 2			8.64
Dashpur 2				8.77
Dashpur2				8.45
Prentice				10.87
Prentice				7.96
Prentice				10.76
Prentice				7.98
Island X				7.94
Ramganga	Maximal degradation			14.05
Atharogazi				14.12
Patharprotima				14.18
Gopalnagar				14.3
Brajaballavpur				14.34
Durbachoti	Pristine	*Bruguiera cylindrica*	Unit of SOD / mg of protein	17.377
ShiberGhat	Intermediate degradation 1			16.241
Island X	Intermediate degradation 2			20.334
Island X				18.675
Atharogazi	Maximal degradation			18.998
Durbachoti	Pristine		Total Na+/K+	1.143
ShiberGhat	Intermediate degradation 1			1.326
Lakkhipur island	Intermediate degradation 2			1.838
Lakkhipur island				2.173
Atharogazi	Maximal degradation			2.344
Lakkhipur	Pristine		Total chlorophyll (mg/100 g of FW)	111.983
Durbachoti				107.523
ShiberGhat	Intermediate degradation 1			88.109
Rakkhoshkhali	Intermediate degradation 2			73.918
Rakkhoshkhali				73.964
Atharogazi	Maximal degradation			66.020
Atharogazi				55.495
Lothian	Pristine		PEPC activity/RuBPC activity (µmol/minute/µg of protein)	5.987
G-Plot				5.996
Dashpur1	Intermediate degradation 1			5.765
Birat				5.758
Dashpur 2	Intermediate degradation 2			5.666
Prentice				5.668
Atharogazi	Maximal degradation			5.560
Atharogazi				5.570
Ramganga				5.560
Durbachoti	Pristine		Leaf thickness (LT) µm	72.880
L-Plot	Intermediate degradation 1			77.278
Dashpur1				75.299
Dashpur1				76.000
Dashpur 2	Intermediate			172.320

Island X	degradation 2			172.220
Island X				181.980
Island X				192.560
Island X				183.780
Island X				172.890
Atharogazi	Maximal degradation			224.960
Patharprotima				233.090
Durbachoti	Pristine		Water storage tissue (WST) µm	24.400
L-Plot	Intermediate degradation 1			34.758
Dashpur1				33.889
Dashpur1				31.340
Dashpur 2	Intermediate degradation 2			96.670
Island X				114.900
Island X				105.380
Island X				118.360
Island X				109.310
Island X				108.370
Atharogazi	Maximal degradation			154.530
Patharprotima				165.550
Durbachoti	Pristine		Palisade tissue (PT) µm	25.030
L-Plot	Intermediate degradation 1			22.050
Dashpur1				20.070
Dashpur1				21.880
Dashpur 2	Intermediate degradation 2			67.090
Island X				48.090
Island X				67.150
Island X				65.990
Island X				67.360
Island X				55.980
Atharogazi	Maximal degradation			68.430
Patharprotima				65.540
Durbachoti	Pristine		Spongy tissue (ST) µm	22.450
L-Plot	Intermediate degradation 1			19.470
Dashpur1				20.340
Dashpur1				21.780
Dashpur 2	Intermediate degradation 2			7.560
Island X				8.230
Island X				8.450
Island X				8.210
Island X				7.110
Island X				7.540
Atharogazi	Maximal degradation			0.800
Patharprotima				1.000
Durbachoti	Pristine		PT/ST	1.115
L-Plot	Intermediate degradation 1			1.133
Dashpur1				0.987
Dashpur1				1.005
Dashpur 2	Intermediate degradation 2			8.874
Island X				5.843
Island X				7.947
Island X				8.038
Island X				9.474
Island X				7.424

Atharogazi	Maximal degradation			85.538
Patharprotima				65.540
Durbachoti	Pristine		PT/LT	0.343
L-Plot	Intermediate degradation 1			0.285
Dashpur1				0.267
Dashpur1				0.288
Dashpur 2	Intermediate degradation 2			0.389
Island X				0.279
Island X				0.369
Island X				0.343
Island X				0.367
Island X				0.324
Atharogazi	Maximal degradation			0.304
Patharprotima				0.281
Durbachoti	Pristine		ST/LT	0.308
L-Plot	Intermediate degradation 1			0.252
Dashpur1				0.270
Dashpur1				0.287
Dashpur 2	Intermediate degradation 2			0.044
Island X				0.048
Island X				0.046
Island X				0.043
Island X				0.039
Island X				0.044
Atharogazi	Maximal degradation			0.004
Patharprotima				0.004
Durbachoti	Pristine		WST/LT	0.335
L-Plot	Intermediate degradation 1			0.450
Dashpur1				0.450
Dashpur1				0.412
Dashpur 2	Intermediate degradation 2			0.561
Island X				0.667
Island X				0.579
Island X				0.615
Island X				0.595
Island X				0.627
Atharogazi	Maximal degradation			0.687
Patharprotima				0.710
Durbachoti	Pristine		*MIPS*(ΔCQ)	1.34
Durbachoti				1.62
G-Plot				2.42
G-Plot				2.54
ShiberGhat	Intermediate degradation 1			4.63
ShiberGhat				4.7
ShiberGhat				4.78
ShiberGhat				4.85
L-Plot				4.87
Prentice	Intermediate degradation 2			8.27
Island X				8.4
Prentice				9.79
Island X				9.82
Prentice				9.84
Island X				10.15

Island X				10.17
Island X				10.2
Brajaballavpur	Maximal degradation			15.32
Atharogazi				15.45
Atharogazi				15.46
Atharogazi				15.54
G-Plot	Pristine		*FBA*(ΔCQ)	5.38
G-Plot				5.45
G-Plot				5.75
G-Plot				5.89
Lothian				5.97
Dashpur1	Intermediate degradation 1			9.06
Birat				9.39
Birat				9.46
Birat				9.55
ShiberGhat				9.18
ShiberGhat				9.16
Dashpur1				9.18
Dashpur1				10.26
Lakkhipur island	Intermediate degradation 2			11.35
Lakkhipur island				11.67
Lakkhipur island				11.69
Rakkhoshkhali				11.88
Rakkhoshkhali				12.45
Rakkhoshkhali				12.5
Rakkhoshkhali				12.63
Dashpur 2				12.64
Prentice				12.88
Prentice				12.82
Gopalnagar	Maximal degradation			15.18
Gopalnagar				15.33
Gopalnagar				15.4
Gopalnagar				15.87
Brajaballavpur				15.91
Brajaballavpur				16.11
Brajaballavpur				16.2
Brajaballavpur				16.23
Brajaballavpur				16.36
Ramganga				16.38
Ramganga				16.42
Lakkhipur	Pristine		*F26BP*(ΔCQ)	3.23
Lakkhipur				3.28
Lakkhipur				3.41
Lothian				3.22
Lothian				2.21
Lothian				3
Dashpur1	Intermediate degradation 1			7.64
Dashpur1				7.62
Dashpur1				7.71
Dashpur1				7.32
Dashpur1				7.54

Birat				7.24
ShiberGhat				7.25
ShiberGhat				8.2
Rakkhoshkhali	Intermediate degradation 2			12.48
Rakkhoshkhali				12.62
Rakkhoshkhali				12.67
Rakkhoshkhali				12.88
Rakkhoshkhali				12.84
Dashpur 2				12.74
Dashpur 2				12.72
Dashpur 2				12.38
Prentice				12.48
Prentice				12.67
Ramganga	Maximal degradation			17.09
Ramganga				17.28
Ramganga				18.29
Ramganga				18.34
Ramganga				18.38
Ramganga				16.44
Durbachoti	Pristine	*Bruguiera gymnorrhiza*	Unit of SOD / mg of protein	8.199
Dashpur1	Intermediate degradation 1			7.055
Island X	Intermediate degradation 2			8.320
Ramganga	Maximal degradation			5.332
Durbachoti	Pristine		Total Na+/K+	1.481
L-Plot	Intermediate degradation 1			1.217
Lakkhipur island	Intermediate degradation 2			1.654
Rakkhoshkhali				1.552
Atharogazi	Maximal degradation			2.857
Lakkhipur	Pristine		Total chlorophyll (mg/100 g of FW)	56.714
ShiberGhat	Intermediate degradation 1			48.567
ShiberGhat				57.894
Lakkhipur island	Intermediate degradation 2			42.487
Rakkhoshkhali				45.678
Atharogazi	Maximal degradation			12.568
Brajaballavpur				16.924
Durbachoti	Pristine		PEPC activity/RuBPC activity (µmol/minute/µg of protein	1.097
L-Plot	Intermediate degradation 1			0.986
Dashpur1				0.987
Rakkhoshkhali	Intermediate degradation 2			0.774
Prentice				0.774
Dashpur 2				0.773
Rakkhoshkhali				0.773
Brajaballavpur	Maximal degradation			0.546
Brajaballavpur				0.547
Lakkhipur	Pristine		Leaf thickness (LT) µm	83.770
ShiberGhat	Intermediate degradation 1			112.090
L-Plot				108.540

L-Plot				111.700
Lakkhipur island	Intermediate degradation 2			180.040
Lakkhipur island				179.050
Lakkhipur island				192.850
Lakkhipur island				211.950
Lakkhipur island				191.950
Rakkhoshkhali				211.950
Gopalnagar	Maximal degradation			322.530
Brajaballavpur				345.740
Atharogazi				328.140
Lakkhipur	Pristine		Water storage tissue (WST) µm	19.280
ShiberGhat	Intermediate degradation 1			53.310
L-Plot				50.570
L-Plot				55.480
Lakkhipur island	Intermediate degradation 2			132.170
Lakkhipur island				128.950
Lakkhipur island				150.570
Lakkhipur island				167.360
Lakkhipur island				141.960
Rakkhoshkhali				162.960
Gopalnagar	Maximal degradation			273.320
Brajaballavpur				301.870
Atharogazi				280.820
Lakkhipur	Pristine		Palisade tissue (PT) µm	33.450
ShiberGhat	Intermediate degradation 1			38.120
L-Plot				40.760
L-Plot				37.770
Lakkhipur island	Intermediate degradation 2			40.660
Lakkhipur island				43.980
Lakkhipur island				35.540
Lakkhipur island				37.540
Lakkhipur island				42.540
Rakkhoshkhali				41.340
Gopalnagar	Maximal degradation			47.210
Brajaballavpur				41.870
Atharogazi				45.320
Lakkhipur	Pristine		Spongy tissue (ST) µm	30.540
ShiberGhat	Intermediate degradation 1			19.660
L-Plot				16.210

L-Plot				17.450
Lakkhipur island	Intermediate degradation 2			6.210
Lakkhipur island				5.120
Lakkhipur island				5.740
Lakkhipur island				6.050
Lakkhipur island				6.450
Rakkhoshkhali				6.650
Gopalnagar	Maximal degradation			1.300
Brajaballavpur				2.000
Atharogazi				1.900
Lakkhipur	Pristine		PT/ST	1.095
ShiberGhat	Intermediate degradation 1			1.939
L-Plot				2.514
L-Plot				2.164
Lakkhipur island	Intermediate degradation 2			6.548
Lakkhipur island				8.590
Lakkhipur island				6.192
Lakkhipur island				6.205
Lakkhipur island				6.595
Rakkhoshkhali				6.217
Gopalnagar	Maximal degradation			36.315
Brajaballavpur				20.935
Atharogazi				23.853
Lakkhipur	Pristine		PT/LT	0.399
ShiberGhat	Intermediate degradation 1			0.340
L-Plot				0.376
L-Plot				0.338
Lakkhipur island	Intermediate degradation 2			0.226
Lakkhipur island				0.246
Lakkhipur island				0.184
Lakkhipur island				0.177
Lakkhipur island				0.222
Rakkhoshkhali				0.195
Gopalnagar	Maximal degradation			0.146
Brajaballavpur				0.121
Atharogazi				0.138
Lakkhipur	Pristine		ST/LT	0.365
ShiberGhat	Intermediate degradation 1			0.175
L-Plot				0.149

L-Plot				0.156
Lakkhipur island	Intermediate degradation 2			0.034
Lakkhipur island				0.029
Lakkhipur island				0.030
Lakkhipur island				0.029
Lakkhipur island				0.034
Rakkhoshkhali				0.031
Gopalnagar	Maximal degradation			0.004
Brajaballavpur				0.006
Atharogazi				0.006
Lakkhipur	Pristine		WST/LT	0.230
ShiberGhat	Intermediate degradation 1			0.476
L-Plot				0.466
L-Plot				0.497
Lakkhipur island	Intermediate degradation 2			0.734
Lakkhipur island				0.720
Lakkhipur island				0.781
Lakkhipur island				0.790
Lakkhipur island				0.740
Rakkhoshkhali				0.769
Gopalnagar	Maximal degradation			0.847
Brajaballavpur				0.873
Atharogazi				0.856
Lothian	Pristine	*Ceriops tagal*	Soluble sugar to starch ratio	2.791
Lakkhipur				2.449
Lakkhipur				2.352
Lakkhipur				3.164
Lakkhipur				2.810
Lakkhipur				1.319
Durbachoti				1.044
Durbachoti				2.164
ShiberGhat	Intermediate degradation 1			5.235
ShiberGhat				5.979
ShiberGhat				4.893
ShiberGhat				5.816
ShiberGhat				4.895
Birat				4.921
L-Plot				5.033
Lakkhipur island	Intermediate degradation 2			7.287
Lakkhipur island				7.154
Rakkhoshkhali				6.872
Rakkhoshkhali				7.090

Rakkhoshkhali				7.695
Rakkhoshkhali				7.725
Rakkhoshkhali				7.695
Dashpur 2				6.638
Prentice				7.358
Island X				7.316
Ramganga	Maximal degradation			8.786
Ramganga				9.161
Ramganga				8.435
Ramganga				8.542
Ramganga				9.899
Lakkhipur	Pristine		Total chlorophyll (mg/100 g of FW)	61.532
Dashpur1	Intermediate degradation 1			60.067
Lakkhipur island	Intermediate degradation 2			35.675
Rakkhoshkhali				42.487
Lakkhipur island				48.563
Atharogazi	Maximal degradation			18.860
Durbachoti	Pristine		Leaf thickness (LT) µm	60.250
L-Plot	Intermediate degradation 1			118.070
L-Plot				129.070
L-Plot				121.450
Prentice	Intermediate degradation 2			149.570
Island X				149.360
Island X				159.450
Gopalnagar	Maximal degradation			199.650
Gopalnagar				200.360
Gopalnagar				191.780
Durbachoti	Pristine		Water storage tissue (WST) µm	7.050
L-Plot	Intermediate degradation 1			71.960
L-Plot				71.710
L-Plot				64.950
Prentice	Intermediate degradation 2			127.440
Island X				122.230
Island X				129.950
Gopalnagar	Maximal degradation			180.200
Gopalnagar				177.480
Gopalnagar				166.100
Durbachoti	Pristine		Palisade tissue (PT) µm	28.480
L-Plot	Intermediate degradation 1			45.110
L-Plot				18.020
L-Plot				17.850
Prentice	Intermediate degradation 2			12.450
Island X				16.980
Island X				20.050
Gopalnagar	Maximal degradation			17.450
Gopalnagar				20.880
Gopalnagar				21.680
Durbachoti	Pristine		Spongy tissue (ST) µm	24.220
L-Plot	Intermediate degradation 1			0.500
L-Plot				38.340

L-Plot				37.650
Prentice	Intermediate degradation 2			6.680
Island X				8.150
Island X				8.450
Gopalnagar	Maximal degradation			0.500
Gopalnagar				1.600
Gopalnagar				3.000
Durbachoti	Pristine		PT/ST	1.176
L-Plot	Intermediate degradation 1			90.220
L-Plot				0.470
L-Plot				0.474
Prentice	Intermediate degradation 2			1.864
Island X				2.083
Island X				2.373
Gopalnagar	Maximal degradation			34.900
Gopalnagar				13.050
Gopalnagar				7.227
Durbachoti	Pristine		PT/LT	0.473
L-Plot	Intermediate degradation 1			0.382
L-Plot				0.140
L-Plot				0.147
Prentice	Intermediate degradation 2			0.083
Island X				0.114
Island X				0.126
Gopalnagar	Maximal degradation			0.087
Gopalnagar				0.104
Gopalnagar				0.113
Durbachoti	Pristine		ST/LT	0.402
L-Plot	Intermediate degradation 1			0.004
L-Plot				0.297
L-Plot				0.310
Prentice	Intermediate degradation 2			0.045
Island X				0.055
Island X				0.053
Gopalnagar	Maximal degradation			0.003
Gopalnagar				0.008
Gopalnagar				0.016
Durbachoti	Pristine		WST/LT	0.117
L-Plot	Intermediate degradation 1			0.609
L-Plot				0.556
L-Plot				0.535
Prentice	Intermediate degradation 2			0.852
Island X				0.818
Island X				0.815
Gopalnagar	Maximal degradation			0.903
Gopalnagar				0.886
Gopalnagar				0.866
Lakkhipur	Pristine	*Ceriops decandra*	Unit of SOD / mg of protein	15.131
Dashpur1	Intermediate degradation 1			15.874
Island X	Intermediate degradation 2			19.886
Island X				15.742
Atharogazi	Maximal degradation			19.722

Durbachoti	Pristine		Total Na+/K+	1.729
ShiberGhat	Intermediate degradation 1			1.763
Prentice	Intermediate degradation 2			3.543
Gopalnagar	Maximal degradation			1.155
Lakkhipur	Pristine		Total chlorophyll (mg/100 g of FW)	57.702
Dashpur1	Intermediate degradation 1			50.674
Lakkhipur island	Intermediate degradation 2			48.378
Lakkhipur island				45.399
Atharogazi	Maximal degradation			24.941
Durbachoti	Pristine		PEPC activity/RuBPC activity (µmol/minute/µg of protein	0.424
L-Plot	Intermediate degradation 1			0.408
Dashpur1				0.409
Dashpur1				0.409
Rakkhoshkhali	Intermediate degradation 2			0.387
Rakkhoshkhali				0.387
Island X				0.386
Prentice				0.387
Gopalnagar	Maximal degradation			0.379
Brajaballavpur				0.380
Lothian	Pristine		Leaf thickness (LT) µm	58.360
Dashpur1	Intermediate degradation 1			75.920
Dashpur1				74.360
Dashpur1				75.690
Dashpur 2	Intermediate degradation 2			221.360
Prentice				209.880
Prentice				222.360
Prentice				233.450
Prentice				222.550
Patharprotima	Maximal degradation			281.660
Patharprotima				278.050
Gopalnagar				270.440
Lothian	Pristine		Water storage tissue (WST) µm	21.241
Dashpur1	Intermediate degradation 1			33.210
Dashpur1				27.900
Dashpur1				25.760
Dashpur 2	Intermediate degradation 2			152.960
Prentice				139.280
Prentice				152.390
Prentice				160.520
Prentice				147.880
Patharprotima	Maximal degradation			247.610
Patharprotima				239.600
Gopalnagar				234.990
Lothian	Pristine		Palisade tissue (PT) µm	19.500
Dashpur1	Intermediate degradation 1			17.350
Dashpur1				24.580
Dashpur1				23.250

Dashpur 2	Intermediate degradation 2			28.150
Prentice				30.050
Prentice				28.750
Prentice				31.450
Prentice				31.450
Patharprotima	Maximal degradation			32.050
Patharprotima				36.450
Gopalnagar				33.450
Lothian	Pristine		Spongy tissue (ST) µm	17.450
Dashpur1	Intermediate degradation 1			24.360
Dashpur1				20.880
Dashpur1				25.680
Dashpur 2	Intermediate degradation 2			38.250
Prentice				39.550
Prentice				40.220
Prentice				39.480
Prentice				41.220
Patharprotima	Maximal degradation			1.100
Patharprotima				0.800
Gopalnagar				0.700
Lothian	Pristine		PT/ST	1.117
Dashpur1	Intermediate degradation 1			0.712
Dashpur1				1.177
Dashpur1				0.905
Dashpur 2	Intermediate degradation 2			0.736
Prentice				0.760
Prentice				0.715
Prentice				0.797
Prentice				0.763
Patharprotima	Maximal degradation			29.136
Patharprotima				45.563
Gopalnagar				47.786
Lothian	Pristine		PT/LT	0.334
Dashpur1	Intermediate degradation 1			0.229
Dashpur1				0.331
Dashpur1				0.307
Dashpur 2	Intermediate degradation 2			0.127
Prentice				0.143
Prentice				0.129
Prentice				0.135
Prentice				0.141
Patharprotima	Maximal degradation			0.114
Patharprotima				0.131
Gopalnagar				0.124
Lothian	Pristine		ST/LT	0.299
Dashpur1	Intermediate degradation 1			0.321
Dashpur1				0.281
Dashpur1				0.339
Dashpur 2	Intermediate degradation 2			0.173
Prentice				0.188
Prentice				0.181
Prentice				0.169
Prentice				0.185

Patharprotima	Maximal degradation			0.004
Patharprotima				0.003
Gopalnagar				0.003
Lothian	Pristine		WST/LT	0.364
Dashpur1	Intermediate degradation 1			0.437
Dashpur1				0.375
Dashpur1				0.340
Dashpur 2	Intermediate degradation 2			0.691
Prentice				0.664
Prentice				0.685
Prentice				0.688
Prentice				0.664
Patharprotima	Maximal degradation			0.879
Patharprotima				0.862
Gopalnagar				0.869
Lothian	Pristine	*Sonneratia apetala*	Mannitol in (nmol/g of FW)	2856.655
Lothian				5732.091
ShiberGhat	Intermediate degradation 1			1942.462
Birat				1942.462
Birat				1859.164
Lakkhipur island	Intermediate degradation 2			1221.000
Rakkhoshkhali				1472.761
Rakkhoshkhali				1259.985
Rakkhoshkhali				1175.701
Ramganga	Maximal degradation			275.188
Atharogazi				330.544
Patharprotima				559.643
Gopalnagar				480.543
Lakkhipur	Pristine		Total Na+/K+	1.782
ShiberGhat	Intermediate degradation 1			3.675
Island X	Intermediate degradation 2			3.576
Lakkhipur island				2.710
Patharprotima	Maximal degradation			4.617
Durbachoti	Pristine		PEPC activity/RuBPC activity (µmol/minute/µg of protein	3.152
Lothian				3.162
L-Plot	Intermediate degradation 1			3.067
Dashpur1				3.066
Dashpur1				3.066
Rakkhoshkhali	Intermediate degradation 2			2.950
Rakkhoshkhali				2.940
Island X				2.940
Prentice				2.930
Brajaballavpur	Maximal degradation			2.897
Brajaballavpur				2.885
Lakkhipur	Pristine		Leaf thickness (LT) µm	59.520
ShiberGhat	Intermediate degradation 1			92.360
Dashpur1				98.360
Dashpur1				94.870
Rakkhoshkhali	Intermediate degradation 2			184.660
Rakkhoshkhali				202.440

Rakkhoshkhali				228.450
Rakkhoshkhali				199.470
Dashpur 2				199.020
Atharogazi	Maximal degradation			336.680
Atharogazi				317.450
Atharogazi				341.990
Lakkhipur	Pristine		Water storage tissue (WST) µm	10.990
ShiberGhat	Intermediate degradation 1			64.027
Dashpur1				67.580
Dashpur1				68.450
Rakkhoshkhali	Intermediate degradation 2			135.620
Rakkhoshkhali				153.880
Rakkhoshkhali				179.200
Rakkhoshkhali				149.910
Dashpur 2				148.810
Atharogazi	Maximal degradation			293.800
Atharogazi				276.800
Atharogazi				294.630
Lakkhipur	Pristine		Palisade tissue (PT) µm	27.880
ShiberGhat	Intermediate degradation 1			25.180
Dashpur1				24.630
Dashpur1				23.078
Rakkhoshkhali	Intermediate degradation 2			41.760
Rakkhoshkhali				45.560
Rakkhoshkhali				47.250
Rakkhoshkhali				47.560
Dashpur 2				48.210
Atharogazi	Maximal degradation			40.880
Atharogazi				38.650
Atharogazi				45.360
Lakkhipur	Pristine		Spongy tissue (ST) µm	19.650
ShiberGhat	Intermediate degradation 1			3.100
Dashpur1				4.000
Dashpur1				2.000
Rakkhoshkhali	Intermediate degradation 2			6.280
Rakkhoshkhali				0.800
Rakkhoshkhali				0.550
Rakkhoshkhali				0.720
Dashpur 2				1.000
Atharogazi	Maximal degradation			1.400
Atharogazi				0.700
Atharogazi				0.500
Lakkhipur	Pristine		PT/ST	1.419
ShiberGhat	Intermediate degradation 1			8.123
Dashpur1				6.158
Dashpur1				11.539
Rakkhoshkhali	Intermediate degradation 2			6.650
Rakkhoshkhali				56.950
Rakkhoshkhali				85.909
Rakkhoshkhali				66.056
Dashpur 2				48.210
Atharogazi	Maximal degradation			29.200
Atharogazi				55.214

Atharogazi				90.720
Lakkhipur	Pristine		PT/LT	0.468
ShiberGhat	Intermediate degradation 1			0.273
Dashpur1				0.250
Dashpur1				0.243
Rakkhoshkhali	Intermediate degradation 2			0.226
Rakkhoshkhali				0.225
Rakkhoshkhali				0.207
Rakkhoshkhali				0.238
Dashpur 2				0.242
Atharogazi	Maximal degradation			0.121
Atharogazi				0.122
Atharogazi				0.133
Lakkhipur	Pristine		ST/LT	0.330
ShiberGhat	Intermediate degradation 1			0.034
Dashpur1				0.041
Dashpur1				0.021
Rakkhoshkhali	Intermediate degradation 2			0.034
Rakkhoshkhali				0.004
Rakkhoshkhali				0.002
Rakkhoshkhali				0.004
Dashpur 2				0.005
Atharogazi	Maximal degradation			0.004
Atharogazi				0.002
Atharogazi				0.001
Lakkhipur	Pristine		WST/LT	0.185
ShiberGhat	Intermediate degradation 1			0.693
Dashpur1				0.687
Dashpur1				0.722
Rakkhoshkhali	Intermediate degradation 2			0.734
Rakkhoshkhali				0.760
Rakkhoshkhali				0.784
Rakkhoshkhali				0.752
Dashpur 2				0.748
Atharogazi	Maximal degradation			0.873
Atharogazi				0.872
Atharogazi				0.862
Durbachoti	Pristine	*Sonneratia caseolaris*	Total Na+/K+	2.064
ShiberGhat	Intermediate degradation 1			4.806
Lakkhipur island	Intermediate degradation 2			2.000
Dashpur 2				2.287
Atharogazi	Maximal degradation			2.791
G-Plot	Pristine		Leaf thickness (LT) µm	79.560
ShiberGhat	Intermediate degradation 1			111.000
ShiberGhat				113.010
ShiberGhat				100.980
ShiberGhat				119.230
Dashpur 2	Intermediate degradation 2			172.690
Dashpur 2				183.450
Dashpur 2				198.950
Atharogazi	Maximal			236.080

Patharprotima	degradation			255.660
Patharprotima				225.660
Patharprotima				239.450
G-Plot	Pristine		Water storage tissue (WST) µm	20.180
ShiberGhat	Intermediate degradation 1			53.700
ShiberGhat				61.980
ShiberGhat				48.440
ShiberGhat				63.240
Dashpur 2	Intermediate degradation 2			129.540
Dashpur 2				137.670
Dashpur 2				154.100
Atharogazi	Maximal degradation			193.450
Patharprotima				213.510
Patharprotima				182.380
Patharprotima				193.790
G-Plot	Pristine		Palisade tissue (PT) µm	56.980
ShiberGhat	Intermediate degradation 1			34.980
ShiberGhat				27.020
ShiberGhat				28.990
ShiberGhat				29.780
Dashpur 2	Intermediate degradation 2			41.150
Dashpur 2				43.780
Dashpur 2				42.850
Atharogazi	Maximal degradation			40.630
Patharprotima				40.150
Patharprotima				41.280
Patharprotima				43.660
G-Plot	Pristine		Spongy tissue (ST) µm	0.800
ShiberGhat	Intermediate degradation 1			21.320
ShiberGhat				23.010
ShiberGhat				22.550
ShiberGhat				25.210
Dashpur 2	Intermediate degradation 2			1.200
Dashpur 2				0.800
Dashpur 2				0.600
Atharogazi	Maximal degradation			0.900
Patharprotima				1.400
Patharprotima				1.800
Patharprotima				1.200
G-Plot	Pristine		PT/ST	71.225
ShiberGhat	Intermediate degradation 1			1.641
ShiberGhat				1.174
ShiberGhat				1.286
ShiberGhat				1.181
Dashpur 2	Intermediate degradation 2			34.292
Dashpur 2				54.725
Dashpur 2				71.417
Atharogazi	Maximal degradation			45.144
Patharprotima				28.679
Patharprotima				22.933
Patharprotima				36.383
G-Plot	Pristine		PT/LT	0.716
ShiberGhat	Intermediate			0.315

ShiberGhat	degradation 1			0.239
ShiberGhat				0.287
ShiberGhat				0.250
Dashpur 2	Intermediate degradation 2			0.238
Dashpur 2				0.239
Dashpur 2				0.215
Atharogazi	Maximal degradation			0.172
Patharprotima				0.157
Patharprotima				0.183
Patharprotima				0.182
G-Plot	Pristine		ST/LT	0.010
ShiberGhat	Intermediate degradation 1			0.192
ShiberGhat				0.204
ShiberGhat				0.223
ShiberGhat				0.211
Dashpur 2	Intermediate degradation 2			0.007
Dashpur 2				0.004
Dashpur 2				0.003
Atharogazi	Maximal degradation			0.004
Patharprotima				0.005
Patharprotima				0.008
Patharprotima				0.005
G-Plot	Pristine		WST/LT	0.254
ShiberGhat	Intermediate degradation 1			0.484
ShiberGhat				0.548
ShiberGhat				0.480
ShiberGhat				0.530
Dashpur 2	Intermediate degradation 2			0.750
Dashpur 2				0.750
Dashpur 2				0.775
Atharogazi	Maximal degradation			0.819
Patharprotima				0.835
Patharprotima				0.808
Patharprotima				0.809
Bhagabatpur	Pristine	*Heritiera fomes*	Glycine betaine in (mg/g of DW)	6.630
Durbachoti				7.470
Dashpur1	Intermediate degradation 1			4.220
Lakkhipur island	Intermediate degradation 2			3.885
Lakkhipur island				3.541
Dashpur 2				3.147
Dashpur 2				5.406
Dashpur 2				5.184
Prentice				4.350
Prentice				4.592
Ramganga	Maximal degradation			2.969
Atharogazi				2.798
Patharprotima				3.250
Bhagabatpur	Pristine		Soluble sugar to starch ratio	6.548
Lakkhipur				5.429
Birat	Intermediate degradation 1			7.988
ShiberGhat				7.777

L-Plot				6.738
Dashpur1				6.660
Dashpur1				6.738
Prentice	Intermediate degradation 2			8.088
Island X				8.879
Lakkhipur island				8.869
Lakkhipur island				7.064
Island X				7.779
Rakkhoshkhali				8.996
Dashpur 2				8.798
Ramganga	Maximal degradation			9.365
Atharogazi				9.943
Patharprotima				9.352
Gopalnagar				9.042
Lakkhipur	Pristine		Free amino acids (mg/g of FW)	0.725
Durbachoti				0.718
Durbachoti				1.178
ShiberGhat	Intermediate degradation 1			1.230
Dashpur1				1.668
Dashpur1				1.521
Prentice	Intermediate degradation 2			1.923
Prentice				1.747
Prentice				1.639
Island X				1.639
Patharprotima	Maximal degradation			4.440
Atharogazi				3.192
Bhagabatpur	Pristine		Mannitol in (nmol/g of FW)	3043.997
Lakkhipur				3316.035
Durbachoti				3356.879
Birat	Intermediate degradation 1			2356.809
ShiberGhat				2186.442
L-Plot				2186.442
Dashpur1				2152.673
Prentice	Intermediate degradation 2			1684.482
Island X				1175.701
Lakkhipur island				1498.090
Rakkhoshkhali				1365.779
Dashpur 2				1843.652
Island X				1523.580
Island X				1729.109
Brajaballavpur	Maximal degradation			734.029
Brajaballavpur				986.564
Ramganga				986.578
Lakkhipur	Pristine		Unit of SOD / mg of protein	10.168
ShiberGhat	Intermediate degradation 1			12.063
Prentice	Intermediate degradation 2			17.455
Prentice				15.986
Atharogazi	Maximal degradation			21.457
G-Plot	Pristine		Total Na+/K+	0.282

ShiberGhat	Intermediate degradation 1			0.991
Dashpur 2	Intermediate degradation 2			1.238
Prentice				1.654
Gopalnagar	Maximal degradation			2.089
G-Plot	Pristine		Total chlorophyll (mg/100 g of FW)	228.631
L-Plot	Intermediate degradation 1			250.578
L-Plot				222.778
L-Plot				236.784
Prentice	Intermediate degradation 2			150.674
Prentice				140.399
Prentice				148.758
Patharprotima	Maximal degradation			100.674
Gopalnagar				98.390
Gopalnagar				89.437
G-Plot	Pristine		PEPC activity/RuBPC activity (µmol/minute/µg of protein	1.728
G-Plot				1.717
Dashpur1	Intermediate degradation 1			0.764
Birat				0.772
Rakkhoshkhali	Intermediate degradation 2			0.347
Dashpur 2				0.347
Prentice				0.346
Gopalnagar	Maximal degradation			0.256
Brajaballavpur				0.245
Ramganga				0.245
Lakkhipur	Pristine		*BADH*(ΔCQ)	16.57
Lakkhipur				16.5
Lakkhipur				16.35
ShiberGhat	Intermediate degradation 1			8.99
L-Plot				8.96
L-Plot				8.8
L-Plot				8.76
Rakkhoshkhali	Intermediate degradation 2			6.43
Rakkhoshkhali				5.87
Rakkhoshkhali				5.86
Rakkhoshkhali				4.96
Dashpur 2				4.91
Dashpur 2				4.85
Patharprotima	Maximal degradation			0.71
Patharprotima				0.68
Patharprotima				0.67
Gopalnagar				0.67
Gopalnagar				0.67
Lakkhipur	Pristine	*Aegiceras corniculatum*	Unit of SOD / mg of protein	5.189
Dashpur1	Intermediate degradation 1			6.589
Dashpur 2	Intermediate degradation 2			8.655
Brajaballavpur	Maximal degradation			7.179
Lakkhipur	Pristine		Total Na+/K+	0.810
Dashpur1	Intermediate degradation 1			1.196

Island X	Intermediate degradation 2			2.267
Island X				2.046
Atharogazi	Maximal degradation			5.008
Durbachoti	Pristine		Total chlorophyll (mg/100 g of FW)	159.562
ShiberGhat	Intermediate degradation 1			95.453
ShiberGhat				86.389
Rakkhoshkhali	Intermediate degradation 2			85.675
Dashpur 2				80.564
Patharprotima	Maximal degradation			79.281
Patharprotima				89.484
Lakkhipur	Pristine		PEPC activity/RuBPC activity (µmol/minute/µg of protein	0.096
Lothian				0.095
ShiberGhat	Intermediate degradation 1			0.095
Dashpur1				0.095
Lakkhipur island	Intermediate degradation 2			0.095
Lakkhipur island				0.096
Island X				0.096
Gopalnagar	Maximal degradation			0.094
Gopalnagar				0.095
Lakkhipur	Pristine	*Aegialitis rotundifolia*	Free amino acids (mg/g of FW)	0.678
Lakkhipur				0.769
Lakkhipur				0.609
Birat	Intermediate degradation 1			1.713
ShiberGhat				1.798
L-Plot				1.635
Dashpur 2	Intermediate degradation 2			1.929
Dashpur 2				1.862
Dashpur 2				1.998
Prentice				1.978
Prentice				1.877
Atharogazi	Maximal degradation			4.139
Atharogazi				3.570
Atharogazi				4.792
G-Plot	Pristine		Mannitol in (nmol/g of FW)	5724.462
Lothian				3493.966
Lakkhipur				4276.710
Birat	Intermediate degradation 1			2156.089
ShiberGhat				2563.611
ShiberGhat				2133.566
Dashpur1				2156.089
Dashpur1				2045.676
Island X	Intermediate degradation 2			1523.580
Island X				1632.577
Island X				1798.728
Lakkhipur island				1945.886
Lakkhipur island				1988.368
Lakkhipur island				1833.627
Lakkhipur				1803.146

island				
Atharogazi	Maximal degradation			1160.000
Atharogazi				1091.600
Lakkhipur	Pristine		Unit of SOD / mg of protein	18.357
Dashpur1	Intermediate degradation 1			17.457
Rakkhoshkhali	Intermediate degradation 2			16.144
Atharogazi	Maximal degradation			15.154
Lakkhipur	Pristine		Total Na+/K+	0.703
Dashpur1	Intermediate degradation 1			2.348
Island X	Intermediate degradation 2			2.603
Patharprotima	Maximal degradation			2.577
Durbachoti	Pristine		Total chlorophyll (mg/100 g of FW)	136.024
L-Plot	Intermediate degradation 1			105.678
Dashpur1				120.548
Dashpur1				108.568
Dashpur 2	Intermediate degradation 2			87.564
Dashpur 2				83.830
Patharprotima	Maximal degradation			90.301
Durbachoti	Pristine		PEPC activity/RuBPC activity (µmol/minute/µg of protein	0.137
L-Plot	Intermediate degradation 1			0.128
Dashpur1				0.129
Lakkhipur island	Intermediate degradation 2			0.117
Lakkhipur island				0.116
Island X				0.116
Dashpur 2				0.116
Gopalnagar	Maximal degradation			0.105
Gopalnagar				0.105
Bhagabatpur	Pristine	*Xylocarpus mekongensis*	Proline in (mg/g of FW)	0.812
Lakkhipur				0.629
Lakkhipur				0.929
Lakkhipur				0.877
Durbachoti				0.916
Durbachoti				0.809
Birat	Intermediate degradation 1			1.177
ShiberGhat				1.234
L-Plot				1.160
Dashpur1				1.033
Dashpur1				1.240
ShiberGhat				1.125
ShiberGhat				1.113
ShiberGhat				1.027
ShiberGhat				1.115
Dashpur 2	Intermediate degradation 2			1.715
Rakkhoshkhali				1.680
Island X				1.735

Island X				1.793
Island X				1.573
Island X				1.604
Lakkhipur island				1.834
Prentice				1.666
Ramganga	Maximal degradation			2.060
Atharogazi				2.114
Patharprotima				1.980
Gopalnagar				2.140
Brajaballavpur				1.987
Durbachoti	Pristine		Unit of SOD / mg of protein	9.189
L-Plot	Intermediate degradation 1			13.542
ShiberGhat				12.228
Dashpur1				10.438
Rakkhoshkhali	Intermediate degradation 2			14.052
Rakkhoshkhali				17.441
Patharprotima	Maximal degradation			17.354
G-Plot	Pristine		Total Na+/K+	0.922
L-Plot	Intermediate degradation 1			1.393
Rakkhoshkhali	Intermediate degradation 2			1.729
Gopalnagar	Maximal degradation			1.075
Lakkhipur	Pristine		PEPC activity/RuBPC activity (µmol/minute/µg of protein)	0.511
ShiberGhat	Intermediate degradation 1			0.427
Birat				0.428
Island X	Intermediate degradation 2			0.334
Atharogazi	Maximal degradation			0.252
Atharogazi				0.251
Bhagabatpur	Pristine		Leaf thickness (LT) µm	56.937
Birat	Intermediate degradation 1			70.280
ShiberGhat				71.368
Dashpur1				69.268
Prentice	Intermediate degradation 2			111.934
Island X				115.289
Lakkhipur island				103.374
Rakkhoshkhali				109.328
Ramganga	Maximal degradation			163.378
Ramganga				178.650
Bhagabatpur	Pristine		Water storage tissue (WST) µm	20.167
Birat	Intermediate degradation 1			29.930
ShiberGhat				28.608
Dashpur1				29.018
Prentice	Intermediate degradation 2			68.404
Island X				73.159
Lakkhipur island				62.954
Rakkhoshkhali				63.268
Ramganga	Maximal			119.798

Ramganga	degradation			153.170
Bhagabatpur	Pristine		Palisade tissue (PT) µm	17.340
Birat	Intermediate degradation 1			20.330
ShiberGhat				21.870
Dashpur1				21.160
Prentice	Intermediate degradation 2			25.870
Island X				24.090
Lakkhipur island				23.090
Rakkhoshkhali				27.750
Ramganga	Maximal degradation			26.540
Ramganga				23.580
Bhagabatpur	Pristine		Spongy tissue (ST) µm	19.230
Birat	Intermediate degradation 1			19.020
ShiberGhat				19.890
Dashpur1				18.090
Prentice	Intermediate degradation 2			16.660
Island X				17.040
Lakkhipur island				16.330
Rakkhoshkhali				17.310
Ramganga	Maximal degradation			13.040
Ramganga				2.000
Bhagabatpur	Pristine		PT/ST	0.902
Birat	Intermediate degradation 1			1.069
ShiberGhat				1.100
Dashpur1				1.170
Prentice	Intermediate degradation 2			1.553
Island X				1.414
Lakkhipur island				1.414
Rakkhoshkhali				1.603
Ramganga	Maximal degradation			2.035
Ramganga				11.790
Bhagabatpur	Pristine		PT/LT	0.305
Birat	Intermediate degradation 1			0.289
ShiberGhat				0.306
Dashpur1				0.305
Prentice	Intermediate degradation 2			0.231
Island X				0.209
Lakkhipur island				0.223
Rakkhoshkhali				0.254
Ramganga	Maximal degradation			0.162
Ramganga				0.132
Bhagabatpur	Pristine		ST/LT	0.338
Birat	Intermediate degradation 1			0.271
ShiberGhat				0.279
Dashpur1				0.261
Prentice	Intermediate degradation 2			0.149
Island X				0.148
Lakkhipur island				0.158
Rakkhoshkhali				0.158

Ramganga	Maximal degradation			0.080
Ramganga				0.011
Bhagabatpur	Pristine		WST/LT	0.354
Birat	Intermediate degradation 1			0.426
ShiberGhat				0.401
Dashpur1				0.419
Prentice	Intermediate degradation 2			0.611
Island X				0.635
Lakkhipur island				0.609
Rakkhoshkhali				0.579
Ramganga	Maximal degradation			0.733
Ramganga				0.857
Lakkhipur	Pristine		*P5CS* (ΔCQ)	1.07
Lakkhipur				1.12
Lakkhipur				1.13
Birat	Intermediate degradation 1			2.64
ShiberGhat				2.69
L-Plot				2.89
Prentice	Intermediate degradation 2			7.34
Island X				7.56
Lakkhipur island				7.63
Rakkhoshkhali				8.6
Dashpur 2				9.16
Island X				9.24
Dashpur 2				6.87
Dashpur 2				7.48
Brajaballavpur	Maximal degradation			11.89
Brajaballavpur				12.78
Brajaballavpur				12.65
Bhagabatpur	Pristine	*Excoecaria agallocha*	Myo-inositol in (mg/g of DW)	8.277
Lakkhipur				8.030
Lakkhipur				5.528
Lakkhipur				5.532
Lakkhipur				7.590
Lothian				5.537
ShiberGhat	Intermediate degradation 1			15.304
L-Plot				13.962
L-Plot				11.314
L-Plot				15.726
L-Plot				12.760
L-Plot				12.750
Dashpur1				15.284
ShiberGhat				11.632
Dashpur1				15.595
Dashpur1				14.764
Prentice	Intermediate degradation 2			18.925
Island X				17.520
Lakkhipur island				19.313
Rakkhoshkhali				16.347
Dashpur 2				17.949
Island X				16.340

Dashpur 2				15.433
Ramganga	Maximal degradation			22.137
Atharogazi				21.815
Patharprotima				21.766
Gopalnagar				30.574
Brajaballavpur				30.598
Atharogazi				25.464
Atharogazi				25.591
G-Plot	Pristine		Free amino acids (mg/g of FW)	0.947
Lothian				0.671
Lakkhipur				0.951
ShiberGhat	Intermediate degradation 1			1.519
ShiberGhat				1.518
Dashpur1				1.574
Rakkhoshkhali	Intermediate degradation 2			1.938
Rakkhoshkhali				1.922
Lakkhipur island				3.790
Rakkhoshkhali				1.824
Rakkhoshkhali				2.483
Dashpur 2				2.479
Gopalnagar	Maximal degradation			4.263
Gopalnagar				3.451
Brajaballavpur				3.600
Brajaballavpur				3.331
Lakkhipur	Pristine		Mannitol in (nmol/g of FW)	10236.715
Lakkhipur				7347.821
Lakkhipur				8272.487
Lakkhipur				7177.023
Durbachoti				8147.330
ShiberGhat	Intermediate degradation 1			4476.198
ShiberGhat				3066.620
L-Plot				3800.214
ShiberGhat				3120.689
Rakkhoshkhali	Intermediate degradation 2			1761.431
Dashpur 2				1744.751
Rakkhoshkhali				2658.924
Dashpur 2				2080.332
Dashpur 2				1976.411
Prentice				1527.283
Brajaballavpur	Maximal degradation			138.414
Atharogazi				147.000
Atharogazi				150.567
Lothian	Pristine		Unit of SOD / mg of protein	28.426
L-Plot	Intermediate degradation 1			27.850
Rakkhoshkhali	Intermediate degradation 2			28.961
Rakkhoshkhali				23.653
Atharogazi	Maximal degradation			28.457
Gopalnagar				27.585
Lakkhipur	Pristine		Total Na+/K+	0.133
Dashpur1	Intermediate degradation 1			1.135
Rakkhoshkhali	Intermediate			0.753

Prentice	degradation 2			0.682
Brajaballavpur	Maximal degradation			0.754
G-Plot	Pristine		Total chlorophyll (mg/100 g of FW)	43.675
Dashpur1	Intermediate degradation 1			54.328
Rakkhoshkhali	Intermediate degradation 2			65.348
Gopalnagar	Maximal degradation			62.917
Durbachoti	Pristine		Leaf thickness (LT) µm	58.480
Durbachoti				37.640
ShiberGhat	Intermediate degradation 1			94.250
ShiberGhat				84.270
Island X	Intermediate degradation 2			225.340
Island X				220.450
Island X				240.350
Rakkhoshkhali				271.680
Rakkhoshkhali				220.360
Rakkhoshkhali				232.480
Brajaballavpur	Maximal degradation			365.660
Brajaballavpur				334.150
Brajaballavpur				356.140
Durbachoti	Pristine		Water storage tissue (WST) µm	7.148
Durbachoti				1.088
ShiberGhat	Intermediate degradation 1			66.450
ShiberGhat				73.680
Island X	Intermediate degradation 2			186.700
Island X				182.980
Island X				205.230
Rakkhoshkhali				235.560
Rakkhoshkhali				180.030
Rakkhoshkhali				190.120
Brajaballavpur	Maximal degradation			337.540
Brajaballavpur				310.050
Brajaballavpur				328.250
Durbachoti	Pristine		Palisade tissue (PT) µm	30.450
Durbachoti				16.080
ShiberGhat	Intermediate degradation 1			25.150
ShiberGhat				5.780
Island X	Intermediate degradation 2			24.900
Island X				23.350
Island X				21.680
Rakkhoshkhali				22.650
Rakkhoshkhali				26.680
Rakkhoshkhali				25.780
Brajaballavpur	Maximal degradation			26.120
Brajaballavpur				21.065
Brajaballavpur				25.890
Durbachoti	Pristine		Spongy tissue (ST) µm	20.550
Durbachoti				19.680
ShiberGhat	Intermediate degradation 1			2.800
ShiberGhat				2.000
Island X	Intermediate			12.740

Island X	degradation 2			12.120
Island X				12.440
Rakkhoshkhali				12.470
Rakkhoshkhali				13.650
Rakkhoshkhali				13.580
Brajaballavpur	Maximal degradation			2.000
Brajaballavpur				1.900
Brajaballavpur				2.600
Durbachoti	Pristine		PT/ST	1.482
Durbachoti				0.817
ShiberGhat	Intermediate degradation 1			8.982
ShiberGhat				2.890
Island X	Intermediate degradation 2			1.954
Island X				1.927
Island X				1.743
Rakkhoshkhali				1.816
Rakkhoshkhali				1.955
Rakkhoshkhali				1.898
Brajaballavpur	Maximal degradation			13.060
Brajaballavpur				11.087
Brajaballavpur				9.958
Durbachoti	Pristine		PT/LT	0.521
Durbachoti				0.427
ShiberGhat	Intermediate degradation 1			0.267
ShiberGhat				0.069
Island X	Intermediate degradation 2			0.110
Island X				0.106
Island X				0.090
Rakkhoshkhali				0.083
Rakkhoshkhali				0.121
Rakkhoshkhali				0.111
Brajaballavpur	Maximal degradation			0.071
Brajaballavpur				0.063
Brajaballavpur				0.073
Durbachoti	Pristine		ST/LT	0.351
Durbachoti				0.523
ShiberGhat	Intermediate degradation 1			0.030
ShiberGhat				0.024
Island X	Intermediate degradation 2			0.057
Island X				0.055
Island X				0.052
Rakkhoshkhali				0.046
Rakkhoshkhali				0.062
Rakkhoshkhali				0.058
Brajaballavpur	Maximal degradation			0.005
Brajaballavpur				0.006
Brajaballavpur				0.007
Durbachoti	Pristine		WST/LT	0.122
Durbachoti				0.029
ShiberGhat	Intermediate degradation 1			0.705
ShiberGhat				0.874
Island X	Intermediate degradation 2			0.829
Island X				0.830

Island X				0.854
Rakkhoshkhali				0.867
Rakkhoshkhali				0.817
Rakkhoshkhali				0.818
Brajaballavpur	Maximal degradation			0.923
Brajaballavpur				0.928
Brajaballavpur				0.922
Bhagabatpur	Pristine		*SUS*(ΔCQ)	2.03
Lakkhipur				2.18
Durbachoti				2.39
G-Plot				2.46
Lothian				2.5
Lakkhipur				2.52
Birat	Intermediate degradation 1			4.28
ShiberGhat				4.29
L-Plot				5.18
Dashpur1				5.26
Dashpur1				5.39
ShiberGhat				5.46
Dashpur 2	Intermediate degradation 2			8.48
Dashpur2				7.81
Prentice				9.66
Prentice				11.78
Island X				8.92
Island X				8.99
Island X				7.82
Island X				9.12
Rakkhoshkhali				9.18
Rakkhoshkhali				9.32
Prentice				7.7
Island X				8.13
Lakkhipur island				8.14
Rakkhoshkhali				8.2
Patharprotima	Maximal degradation			11.81
Gopalnagar				11.86
Brajaballavpur				11.88
Atharogazi				12.2
Atharogazi				12.37
Atharogazi				12.77
G-Plot	Pristine	*Acanthus ilicifolius*	Glycine betaine in (mg/g of DW)	11.783
Lothian				11.649
Lakkhipur				10.234
Lakkhipur				19.187
Dashpur1	Intermediate degradation 1			7.297
Dashpur1				9.976
Dashpur1				8.124
Dashpur1				9.004
Birat				8.134
Dashpur1				9.896
Dashpur1				7.784
Dashpur 2	Intermediate degradation 2			4.350
Island X				3.539
Rakkhoshkhali				3.147

Rakkhoshkhali				4.249
Rakkhoshkhali				4.306
Rakkhoshkhali				3.475
Rakkhoshkhali				3.539
Dashpur 2				3.218
Rakkhoshkhali				4.745
Rakkhoshkhali				5.056
Patharprotima	Maximal degradation			1.009
Patharprotima				1.054
Patharprotima				1.084
Lakkhipur	Pristine		Pinitol in (µg/g) of FW	967.560
Lakkhipur				945.986
ShiberGhat	Intermediate degradation 1			789.540
ShiberGhat				790.347
ShiberGhat				789.997
Prentice	Intermediate degradation 2			1060.914
Prentice				1079.009
Prentice				694.105
Rakkhoshkhali				694.678
Rakkhoshkhali				683.736
Rakkhoshkhali				684.357
Rakkhoshkhali				680.275
Rakkhoshkhali				676.666
Island X				694.876
Island X				683.939
Brajaballavpur	Maximal degradation			615.112
Brajaballavpur				564.329
Brajaballavpur				625.879
Durbachoti	Pristine		Unit of SOD / mg of protein	7.910
L-Plot	Intermediate degradation 1			11.662
Prentice	Intermediate degradation 2			19.477
Island X				20.537
Ramganga	Maximal degradation			26.164
Brajaballavpur				26.841
Lothian	Pristine		Total Na+/K+	1.125
Dashpur1	Intermediate degradation 1			0.991
L-Plot				0.966
Lakkhipur island	Intermediate degradation 2			1.341
Rakkhoshkhali				1.238
Patharprotima	Maximal degradation			1.728
G-Plot	Pristine		Total chlorophyll (mg/100 g of FW)	36.643
Dashpur1	Intermediate degradation 1			54.388
Dashpur 2	Intermediate degradation 2			49.398
Gopalnagar	Maximal degradation			49.075
Lakkhipur	Pristine		PEPC activity/RuBPC activity (µmol/minute/µg of protein	0.576
ShiberGhat	Intermediate degradation 1			0.500
L-Plot				0.512
ShiberGhat				0.511

Island X	Intermediate degradation 2			0.422
Patharprotima	Maximal degradation			0.275
Patharprotima				0.265
Lakkhipur	Pristine		*SUS*(ΔCQ)	2.73
Lakkhipur				2.81
Lakkhipur				3.06
Lakkhipur				3.28
Durbachoti				3.33
Durbachoti				3.34
ShiberGhat	Intermediate degradation 1			5.84
ShiberGhat				5.85
ShiberGhat				5.92
ShiberGhat				6.06
L-Plot				6.11
L-Plot				6.16
Prentice	Intermediate degradation 2			11.79
Dashpur 2				8.38
Island X				8.61
Island X				8.63
Island X				8.92
Island X				8.99
Island X				9
Lakkhipur island				9.12
Lakkhipur island				9.15
Lakkhipur island				9.19
Lakkhipur island				9.25
Lakkhipur island				9.77
Rakkhoshkhali				9.94
Atharogazi	Maximal degradation			12.86
Atharogazi				13.09
Patharprotima				13.13
Patharprotima				13.19
Patharprotima				13.28
Patharprotima				13.75
Gopalnagar				12.88
Gopalnagar				12.96
G-Plot	Pristine	*Volkameria inermis*	Proline in (mg/g of FW)	0.391
Lothian				0.232
G-Plot				0.348
ShiberGhat	Intermediate degradation 1			0.641
ShiberGhat				0.727
ShiberGhat				0.745
Dashpur1				0.606
Dashpur1				0.646
Island X	Intermediate degradation 2			1.198
Island X				1.762
Rakkhoshkhali				1.477
Rakkhoshkhali				1.688

Rakkhoshkhali				1.274
Rakkhoshkhali				1.906
Rakkhoshkhali				1.992
Prentice				1.358
Island X				1.233
Lakkhipur island				1.704
Ramganga	Maximal degradation			2.934
Ramganga				2.480
Ramganga				2.000
Durbachoti	Pristine		Myo-inositol in (mg/g of DW)	3.507
G-Plot				2.345
G-Plot				3.562
Lothian				3.559
Lothian				6.930
Lothian				6.924
Dashpur1	Intermediate degradation 1			13.467
ShiberGhat				14.177
ShiberGhat				10.242
ShiberGhat				14.079
Island X	Intermediate degradation 2			22.088
Island X				18.423
Island X				24.556
Prentice				17.416
Dashpur 2				24.693
Dashpur 2				17.416
Prentice				18.087
Gopalnagar	Maximal degradation			28.313
Gopalnagar				28.385
Gopalnagar				34.374
Gopalnagar				34.349
Brajaballavpur				30.463
G-Plot	Pristine		Soluble sugar to starch ratio	2.989
G-Plot				2.878
Lothian				2.638
Lothian				3.017
L-Plot	Intermediate degradation 1			5.452
L-Plot				4.512
L-Plot				5.299
L-Plot				4.921
Dashpur1				5.141
Dashpur1				5.178
Dashpur 2	Intermediate degradation 2			6.725
Prentice				6.469
Dashpur 2				6.470
Dashpur 2				6.997
Prentice				7.748
Prentice				7.688
Prentice				7.010
Prentice				7.116
Island X				6.830
Ramganga	Maximal degradation			11.744
Patharprotima				10.149
Patharprotima				10.348

Patharprotima				10.009
Atharogazi				12.589
Atharogazi				12.575
Durbachoti	Pristine		Free amino acids (mg/g of FW)	0.497
Durbachoti				0.595
ShiberGhat	Intermediate degradation 1			1.455
Birat				1.168
Birat				1.167
Lakkhipur island	Intermediate degradation 2			3.003
Lakkhipur island				2.034
Prentice				2.000
Ramganga	Maximal degradation			9.973
Atharogazi				9.968
Patharprotima				7.250
Gopalnagar				4.446
Durbachoti	Pristine		Unit of SOD / mg of protein	4.190
L-Plot	Intermediate degradation 1			6.882
Island X	Intermediate degradation 2			5.643
Dashpur 2				5.442
Patharprotima	Maximal degradation			3.820
Lothian	Pristine		Total Na+/K+	5.212
Dashpur1	Intermediate degradation 1			3.342
ShiberGhat				3.040
Rakkhoshkhali	Intermediate degradation 2			1.579
Prentice				0.648
Ramganga	Maximal degradation			0.700
Lakkhipur	Pristine		Total chlorophyll (mg/100 g of FW)	79.946
Birat	Intermediate degradation 1			81.074
Rakkhoshkhali	Intermediate degradation 2			79.398
Gopalnagar	Maximal degradation			77.614
Brajaballavpur				85.969
G-Plot	Pristine		Leaf thickness (LT) µm	35.140
G-Plot				38.960
Lothian				33.470
Lothian				46.330
Lothian				39.650
Lothian				42.250
Lothian				43.080
Birat	Intermediate degradation 1			53.020
Birat				77.650
Dashpur2	Intermediate degradation 2			125.520
Prentice				120.650
Prentice				124.470
Prentice				119.650
Ramganga	Maximal degradation			156.450
Ramganga				155.740
G-Plot	Pristine		Water storage tissue (WST) µm	6.166

G-Plot				5.034
Lothian				8.420
Lothian				2.147
Lothian				5.281
Lothian				1.111
Lothian				5.370
Birat	Intermediate degradation 1			23.640
Birat				48.020
Dashpur2	Intermediate degradation 2			100.160
Prentice				96.280
Prentice				98.990
Prentice				98.170
Ramganga	Maximal degradation			130.000
Ramganga				130.270
G-Plot	Pristine		Palisade tissue (PT) µm	15.120
G-Plot				17.250
Lothian				14.370
Lothian				22.280
Lothian				20.360
Lothian				23.480
Lothian				20.360
Birat	Intermediate degradation 1			25.310
Birat				25.050
Dashpur2	Intermediate degradation 2			22.060
Prentice				21.680
Prentice				21.360
Prentice				20.069
Ramganga	Maximal degradation			20.480
Ramganga				22.780
G-Plot	Pristine		Spongy tissue (ST) µm	13.360
G-Plot				16.370
Lothian				9.680
Lothian				21.580
Lothian				13.480
Lothian				16.690
Lothian				16.350
Birat	Intermediate degradation 1			2.000
Birat				3.200
Dashpur2	Intermediate degradation 2			0.900
Prentice				1.700
Prentice				2.000
Prentice				0.800
Ramganga	Maximal degradation			4.000
Ramganga				2.000
G-Plot	Pristine		PT/ST	1.132
G-Plot				1.054
Lothian				1.485
Lothian				1.032
Lothian				1.510
Lothian				1.407
Lothian				1.245
Birat	Intermediate degradation 1			12.655
Birat				7.828

Dashpur2	Intermediate degradation 2			24.511
Prentice				12.753
Prentice				10.680
Prentice				25.086
Ramganga	Maximal degradation			5.120
Ramganga				11.390
G-Plot	Pristine		PT/LT	0.430
G-Plot				0.443
Lothian				0.429
Lothian				0.481
Lothian				0.513
Lothian				0.556
Lothian				0.473
Birat	Intermediate degradation 1			0.477
Birat				0.323
Dashpur2	Intermediate degradation 2			0.176
Prentice				0.180
Prentice				0.172
Prentice				0.168
Ramganga	Maximal degradation			0.131
Ramganga				0.146
G-Plot	Pristine		ST/LT	0.380
G-Plot				0.420
Lothian				0.289
Lothian				0.466
Lothian				0.340
Lothian				0.395
Lothian				0.380
Birat	Intermediate degradation 1			0.038
Birat				0.041
Dashpur2	Intermediate degradation 2			0.007
Prentice				0.014
Prentice				0.016
Prentice				0.007
Ramganga	Maximal degradation			0.026
Ramganga				0.013
G-Plot	Pristine		WST/LT	0.175
G-Plot				0.129
Lothian				0.252
Lothian				0.046
Lothian				0.133
Lothian				0.026
Lothian				0.125
Birat	Intermediate degradation 1			0.446
Birat				0.618
Dashpur2	Intermediate degradation 2			0.798
Prentice				0.798
Prentice				0.795
Prentice				0.820
Ramganga	Maximal degradation			0.831
Ramganga				0.836
Lakkhipur	Pristine	*Thespesia populnea*	Glycine betaine in (mg/g of DW)	10.648
Lakkhipur				14.227

Lakkhipur				20.043
Lakkhipur				20.949
Durbachoti				14.523
Durbachoti				14.189
L-Plot	Intermediate degradation 1			8.365
Dashpur1				8.123
ShiberGhat				8.485
ShiberGhat				8.942
ShiberGhat				8.124
Dashpur1				8.024
Lakkhipur island	Intermediate degradation 2			5.541
Prentice				6.536
Prentice				3.195
Prentice				4.990
Gopalnagar	Maximal degradation			3.261
Brajaballavpur				1.844
Atharogazi				1.850
Atharogazi				2.520
Atharogazi				2.530
Atharogazi				3.199
Atharogazi				3.257
Bhagabatpur	Pristine		Free amino acids (mg/g of FW)	0.512
G-Plot				0.590
Dashpur1	Intermediate degradation 1			1.162
Dashpur1				1.162
Dashpur1				1.022
Rakkhoshkhali	Intermediate degradation 2			2.487
Dashpur 2				2.887
Island X				3.758
Island X				3.826
Rakkhoshkhali				2.323
Island X				2.635
Ramganga	Maximal degradation			3.459
Ramganga				3.463
Brajaballavpur				4.279
Lakkhipur	Pristine		Unit of SOD / mg of protein	16.893
Birat	Intermediate degradation 1			15.261
Dashpur 2	Intermediate degradation 2			20.621
Ramganga	Maximal degradation			14.794
G-Plot	Pristine		Total Na+/K+	1.632
Birat	Intermediate degradation 1			0.950
Rakkhoshkhali	Intermediate degradation 2			2.007
Patharprotima	Maximal degradation			2.134
G-Plot	Pristine		Total chlorophyll (mg/100 g of FW)	58.851
Birat	Intermediate degradation 1			50.785
Birat				43.674
Rakkhoshkhali	Intermediate			52.569

Dashpur 2	degradation 2			44.670
Patharprotima	Maximal degradation			44.657
Ramganga				46.389
Bhagabatpur	Pristine		*MIPS*(ΔCQ)	1.2
Lakkhipur				1.25
Durbachoti				1.31
Birat	Intermediate degradation 1			4.07
ShiberGhat				4.09
L-Plot				4.17
Dashpur1				4.43
Dashpur1				4.46
ShiberGhat				4.52
Rakkhoshkhali	Intermediate degradation 2			8.34
Dashpur 2				8.54
Dashpur 2				9.43
Dashpur 2				9.61
Dashpur 2				7.14
Dashpur 2				8.24
Prentice				7.97
Island X				8.16
Ramganga	Maximal degradation			15.14
Atharogazi				15.22
Patharprotima				15.23
Gopalnagar				15.31
Durbachoti	Pristine	*Derris trifoliata*	Proline in (mg/g of FW)	0.410
Durbachoti				0.222
Durbachoti				0.301
G-Plot				0.279
G-Plot				0.467
G-Plot				0.246
G-Plot				0.202
ShiberGhat	Intermediate degradation 1			0.638
L-Plot				0.701
L-Plot				0.605
L-Plot				0.640
L-Plot				0.573
L-Plot				0.698
Dashpur1				0.724
Lakkhipur island	Intermediate degradation 2			1.340
Lakkhipur island				1.577
Lakkhipur island				1.961
Lakkhipur island				1.028
Rakkhoshkhali				1.029
Rakkhoshkhali				1.032
Island X				1.680
Dashpur 2				1.045
Dashpur 2				1.034
Prentice				1.033
Atharogazi	Maximal degradation			2.205
Atharogazi				2.546

Atharogazi				3.367
Atharogazi				3.189
G-Plot	Pristine		Pinitol in (µg/g) of FW	926.436
Lothian				967.787
Lothian				945.355
Dashpur1	Intermediate degradation 1			1206.675
Dashpur1				1247.735
Dashpur1				1246.974
Dashpur1				1253.820
Dashpur1				1280.657
Island X	Intermediate degradation 2			1103.115
Island X				1081.725
Island X				1080.570
Island X				1079.794
Island X				1060.418
Dashpur 2				1578.826
Dashpur 2				1600.747
Dashpur 2				1599.174
Dashpur 2				1545.875
Dashpur 2				1676.043
Gopalnagar	Maximal degradation			887.713
Gopalnagar				950.776
Brajaballavpur				655.121
Brajaballavpur				615.002
Ramganga				1067.597
Ramganga				985.690
G-Plot	Pristine		Free amino acids (mg/g of FW)	0.591
G-Plot				0.662
Dashpur1	Intermediate degradation 1			2.090
L-Plot				1.981
L-Plot				1.977
Island X	Intermediate degradation 2			2.404
Island X				2.872
Rakkhoshkhali				2.893
Dashpur 2				2.785
Patharprotima	Maximal degradation			4.349
Patharprotima				6.094
Ramganga				6.204
G-Plot	Pristine		Mannitol in (nmol/g of FW)	6170.085
Lothian				6468.437
Lothian				14357.794
Lothian				11857.073
Dashpur1	Intermediate degradation 1			3326.319
Dashpur1				2576.318
Dashpur1				2925.272
Birat				2927.177
Island X	Intermediate degradation 2			1293.391
Island X				1191.305
Island X				1632.428
Island X				1421.035
Island X				1501.079
Rakkhoshkhali				1865.979
Rakkhoshkhali				1365.779

Rakkhoshkhali				1523.580
Rakkhoshkhali				1555.964
Prentice				1769.735
Gopalnagar	Maximal degradation			130.727
Gopalnagar				119.046
Ramganga				264.422
Ramganga				146.279
Ramganga				117.670
G-Plot	Pristine		Unit of SOD / mg of protein	18.269
Dashpur1	Intermediate degradation 1			12.659
Dashpur 2	Intermediate degradation 2			35.438
Prentice				25.642
Brajaballavpur	Maximal degradation			73.593
Ramganga				68.849
Lakkhipur	Pristine		Total Na+/K+	0.569
Birat	Intermediate degradation 1			0.198
Dashpur 2	Intermediate degradation 2			0.126
Dashpur 2				0.205
Brajaballavpur	Maximal degradation			0.121
Lothian	Pristine		Total chlorophyll (mg/100 g of FW)	264.688
G-Plot				209.204
Dashpur1	Intermediate degradation 1			180.564
Birat				188.093
Birat				190.540
Island X	Intermediate degradation 2			207.540
Island X				201.389
Rakkhoshkhali				200.892
Rakkhoshkhali				197.430
Brajaballavpur	Maximal degradation			214.164
Brajaballavpur				198.485
Lakkhipur	Pristine		PEPC activity/RuBPC activity (µmol/minute/µg of protein	0.165
Lothian				0.166
ShiberGhat	Intermediate degradation 1			0.156
ShiberGhat				0.156
Island X	Intermediate degradation 2			0.149
Island X				0.149
Prentice				0.148
Dashpur 2				0.149
Patharprotima	Maximal degradation			0.146
Patharprotima				0.145
Bhagabatpur	Pristine		*P5CS* (ΔCQ)	1.47
Lakkhipur				1.62
Durbachoti				1.71
Dashpur1	Intermediate degradation 1			3.47
Dashpur1				3.52
ShiberGhat				3.58
Dashpur 2	Intermediate degradation 2			8.65
Dashpur 2				9.51
Prentice				8.56
Prentice				9.47
Prentice				7.64

Prentice				8.57
Island X				7.81
Patharprotima	Maximal degradation			11.09
Gopalnagar				11.15
Brajaballavpur				11.16
Lothian	Pristine		*BADH*(ΔCQ)	16.77
Lakkhipur				16.66
Lakkhipur				16.58
ShiberGhat	Intermediate degradation 1			9.42
ShiberGhat				9.36
ShiberGhat				9.23
ShiberGhat				9.13
Island X	Intermediate degradation 2			7.32
Island X				7.23
Lakkhipur island				7.08
Lakkhipur island				6.9
Lakkhipur island				6.87
Lakkhipur island				6.83
Lakkhipur island				6.75
Rakkhoshkhali				6.7
Atharogazi	Maximal degradation			0.97
Atharogazi				0.97
Atharogazi				0.96
Atharogazi				0.92
Patharprotima				0.87
Patharprotima				0.78
G-Plot	Pristine		*F16BP*(ΔCQ)	4.91
Lothian				4.94
Lakkhipur				4.97
Lothian				5.32
Lothian				4.98
ShiberGhat	Intermediate degradation 1			7.9
ShiberGhat				7.93
ShiberGhat				7.94
ShiberGhat				7.92
ShiberGhat				7.94
ShiberGhat				7.68
Dashpur1				8.15
Island X	Intermediate degradation 2			11.05
Island X				11.19
Island X				11.21
Lakkhipur island				11.22
Lakkhipur island				11.28
Lakkhipur island				11.34
Lakkhipur island				11.41

Lakkhipur island				11.54
Gopalnagar	Maximal degradation			13.09
Brajaballavpur				12.11
Atharogazi				13.2
Atharogazi				13.62
Atharogazi				13.74
Atharogazi				13.87
Patharprotima				13.96
Durbachoti	Pristine		*SUS*(ΔCQ)	3.42
Durbachoti				3.6
Durbachoti				3.96
G-Plot				3.98
G-Plot				3.98
G-Plot				4.02
L-Plot	Intermediate degradation 1			6.21
L-Plot				6.87
L-Plot				6.9
Dashpur1				6.91
Dashpur1				6.93
Dashpur1				6.98
Dashpur1				7.02
Dashpur1				7.24
Rakkhoshkhali	Intermediate degradation 2			9.96
Rakkhoshkhali				10
Rakkhoshkhali				10.01
Rakkhoshkhali				10.07
Dashpur 2				10.1
Dashpur 2				10.11
Dashpur 2				10.75
Dashpur 2				10.8
Dashpur 2				10.82
Prentice				10.92
Ramganga	Maximal degradation			14.45
Atharogazi				14.46
Patharprotima				14.04
Gopalnagar				14.06
Gopalnagar				14.17
Gopalnagar				14.19
Brajaballavpur				14.24
Brajaballavpur				14.36
Bhagabatpur	Pristine	*Dalbergia spinosa*	Pinitol in (μg/g) of FW	1373.699
Lakkhipur				1486.993
Durbachoti				1487.269
G-Plot				1489.675
Lothian				1598.432
G-Plot				1373.387
G-Plot				1487.376
G-Plot				1489.378
Birat	Intermediate degradation 1			1206.340
ShiberGhat				1247.005
L-Plot				1246.913
Dashpur1				1253.950
Dashpur1				1280.450

ShiberGhat				1206.435
Dashpur1				1224.268
Dashpur1				1246.888
Dashpur1				1280.655
Prentice	Intermediate degradation 2			1103.547
Lakkhipur island				1080.999
Island X				1081.154
Rakkhoshkhali				1060.678
Dashpur 2				1079.238
Rakkhoshkhali				1103.847
Rakkhoshkhali				1081.454
Rakkhoshkhali				1080.399
Rakkhoshkhali				1060.278
Rakkhoshkhali				1079.738
Ramganga	Maximal degradation			983.426
Atharogazi				940.437
Patharprotima				940.044
Gopalnagar				983.926
Gopalnagar				940.137
Gopalnagar				940.894
G-Plot	Pristine		Myo-inositol in (mg/g of DW)	7.856
Dashpur1	Intermediate degradation 1			14.801
Dashpur1				12.886
Birat				12.051
Birat				13.769
Birat				10.822
Dashpur 2	Intermediate degradation 2			24.204
Dashpur 2				26.175
Dashpur 2				21.135
Prentice				20.927
Prentice				18.980
Prentice				17.118
Prentice				16.862
Prentice				18.087
Rakkhoshkhali				24.245
Rakkhoshkhali				26.035
Patharprotima	Maximal degradation			35.873
Patharprotima				32.053
Patharprotima				32.077
Patharprotima				29.254
Gopalnagar				29.088
Durbachoti	Pristine		Free amino acids (mg/g of FW)	0.747
Birat	Intermediate degradation 1			1.219
Birat				1.212
Birat				1.587
ShiberGhat				1.324
Dashpur 2	Intermediate degradation 2			2.872
Rakkhoshkhali				3.072
Dashpur 2				3.978
Lakkhipur island				3.079
Prentice				2.487
Prentice				2.893

Prentice				3.826
Island X				2.323
Rakkhoshkhali				2.636
Rakkhoshkhali				2.633
Ramganga	Maximal degradation			3.473
Ramganga				4.443
Durbachoti	Pristine		Mannitol in (nmol/g of FW)	4044.972
G-Plot				5300.961
G-Plot				5802.427
G-Plot				6007.568
G-Plot				8178.689
L-Plot	Intermediate degradation 1			2987.510
L-Plot				3130.542
L-Plot				2588.130
Dashpur1				2968.228
Dashpur1				2719.724
Dashpur 2	Intermediate degradation 2			2295.779
Prentice				2060.755
Prentice				1970.247
Prentice				2103.966
Prentice				1815.426
Prentice				1748.034
Dashpur 2				1702.683
Rakkhoshkhali				1744.751
Dashpur 2				1976.411
Prentice				1815.426
Atharogazi	Maximal degradation			441.225
Patharprotima				378.385
Patharprotima				229.672
Patharprotima				442.508
Patharprotima				378.797
Patharprotima				428.366
Gopalnagar				131.735
Gopalnagar				171.059
Lothian	Pristine		Unit of SOD / mg of protein	3.189
Dashpur1	Intermediate degradation 1			9.421
Birat				8.132
Prentice	Intermediate degradation 2			9.452
Prentice				9.993
Dashpur 2				7.854
Atharogazi	Maximal degradation			6.129
Lothian	Pristine		Total Na+/K+	1.715
Dashpur1	Intermediate degradation 1			1.729
Dashpur 2	Intermediate degradation 2			5.000
Dashpur 2				5.356
Dashpur 2				4.782
Brajaballavpur	Maximal degradation			4.753
Lothian	Pristine		PEPC activity/RuBPC activity (µmol/minute/µg of protein)	0.569
Lothian				0.559
ShiberGhat	Intermediate degradation 1			0.456

Prentice	Intermediate degradation 2			0.504
Dashpur 2				0.504
Patharprotima	Maximal degradation			0.499
Lakkhipur	Pristine		*P5CS* (ΔCQ)	1.23
Durbachoti				1.32
Durbachoti				1.4
Birat	Intermediate degradation 1			2.889
Birat				3.889
Birat				3.78
Rakkhoshkhali	Intermediate degradation 2			8.77
Rakkhoshkhali				8.09
Rakkhoshkhali				8.65
Dashpur 2				7.44
Dashpur 2				9.19
Dashpur 2				9.82
Dashpur 2				7.88
Ramganga	Maximal degradation			11.01
Atharogazi				11.07
Durbachoti	Pristine	*Caesalpinia crista*	Pinitol in (µg/g) of FW	3045.314
Durbachoti				2982.883
Durbachoti				2982.988
Durbachoti				2896.980
Durbachoti				3006.356
Lothian				3045.566
Lothian				2982.567
Lothian				2896.399
L-Plot	Intermediate degradation 1			2200.786
L-Plot				2245.203
L-Plot				2245.207
L-Plot				2367.950
L-Plot				2166.874
Birat				2200.677
Birat				2245.355
ShiberGhat				2367.569
ShiberGhat				2166.788
Lakkhipur island	Intermediate degradation 2			1578.875
Lakkhipur island				1600.494
Lakkhipur island				1599.997
Lakkhipur island				1676.843
Lakkhipur island				1545.765
Prentice				1578.275
Prentice				1600.249
Prentice				1599.500
Prentice				1676.984
Prentice				1545.177
Patharprotima	Maximal degradation			1067.660
Patharprotima				985.970
Patharprotima				1096.876

Patharprotima				1050.169
Patharprotima				1050.199
Ramganga				1096.668
Ramganga				1050.317
Ramganga				1050.560
Ramganga				950.109
Durbachoti	Pristine		Myo-inositol in (mg/g of DW)	7.520
G-Plot				7.429
G-Plot				7.864
Birat	Intermediate degradation 1			11.634
Birat				11.558
ShiberGhat				13.467
Rakkhoshkhali	Intermediate degradation 2			24.360
Rakkhoshkhali				23.784
Rakkhoshkhali				23.384
Rakkhoshkhali				26.727
Rakkhoshkhali				20.876
Dashpur 2				19.959
Dashpur 2				19.995
Rakkhoshkhali				23.384
Patharprotima	Maximal degradation			35.342
Ramganga				30.469
Ramganga				34.468
Ramganga				38.268
Durbachoti	Pristine		Mannitol in (nmol/g of FW)	6832.844
Durbachoti				6511.797
Durbachoti				5143.619
L-Plot	Intermediate degradation 1			3286.241
ShiberGhat				2588.130
ShiberGhat				3519.750
Dashpur1				2576.318
Birat				2156.089
Dashpur1				4163.434
Dashpur 2	Intermediate degradation 2			2524.516
Dashpur 2				2722.000
Dashpur 2				2560.750
Dashpur 2				2128.364
Brajaballavpur	Maximal degradation			641.803
Brajaballavpur				702.519
Brajaballavpur				712.064
Gopalnagar				704.998
Ramganga				393.698
Ramganga				468.724
Lothian	Pristine		Unit of SOD / mg of protein	37.707
Birat	Intermediate degradation 1			40.652
Dashpur 2	Intermediate degradation 2			26.167
Island X				24.866
Rakkhoshkhali				25.322
Dashpur 2				24.866
Patharprotima	Maximal degradation			39.000
Lothian	Pristine		Total Na+/K+	0.122
Dashpur1	Intermediate			0.275

	degradation 1			
Island X	Intermediate degradation 2			0.479
Brajaballavpur	Maximal degradation			0.219
Lothian	Pristine		Total chlorophyll (mg/100 g of FW)	98.150
Lothian				73.065
ShiberGhat	Intermediate degradation 1			98.084
Prentice	Intermediate degradation 2			99.390
Island X				100.480
Dashpur 2				105.348
Dashpur 2				99.378
Dashpur 2				98.348
Prentice				112.684
Ramganga	Maximal degradation			100.548
Ramganga				102.987
G-Plot	Pristine	*Intsia bijuga*	Unit of SOD / mg of protein	45.450
Birat	Intermediate degradation 1			42.121
Birat				45.765
Island X	Intermediate degradation 2			37.675
Dashpur 2				38.891
Ramganga	Maximal degradation			45.632
Ramganga				49.396
G-Plot	Pristine		Total Na+/K+	0.248
Birat	Intermediate degradation 1			0.106
Birat				0.297
Island X	Intermediate degradation 2			0.405
Island X				0.204
Ramganga	Maximal degradation			0.753
Ramganga				0.377
G-Plot	Pristine		*P5CS* (ΔCQ)	1.5
G-Plot				1.96
G-Plot				1.67
Dashpur1	Intermediate degradation 1			3.56
Birat				3.87
ShiberGhat				4.21
Island X	Intermediate degradation 2			9.31
Island X				9.39
Island X				9.39
Island X				9.48
Lakkhipur island				9.49
Lakkhipur island				9.57
Atharogazi	Maximal degradation			11.27
Atharogazi				11.62
Atharogazi				12.03
Bhagabatpur	Pristine		*BADH*(ΔCQ)	16.92
Lakkhipur				16.89
Durbachoti				16.84
G-Plot				16.8
Birat	Intermediate degradation 1			9.64
ShiberGhat				9.55

L-Plot				9.54
Dashpur1				9.45
Dashpur1				9.42
Prentice	Intermediate degradation 2			7.79
Island X				7.77
Lakkhipur island				7.69
Rakkhoshkhali				7.67
Dashpur 2				7.57
Island X				7.46
Island X				7.37
Island X				7.34
Ramganga	Maximal degradation			2.09
Atharogazi				2.05
Patharprotima				1.71
Gopalnagar				1.6
Brajaballavpur				1.55
Atharogazi				1.03
Durbachoti	Pristine		*F26BP*(ΔCQ)	2.02
G-Plot				2.05
Durbachoti				1.93
Durbachoti				2.12
Durbachoti				2.1
Birat	Intermediate degradation 1			4.05
ShiberGhat				4.25
L-Plot				4.39
Dashpur1				5.96
Dashpur1				6.17
ShiberGhat				7.1
Prentice	Intermediate degradation 2			8.4
Island X				8.45
Lakkhipur island				9.15
Rakkhoshkhali				9.15
Dashpur 2				9.24
Island X				9.52
Island X				9.72
Island X				9.77
Island X				9.81
Atharogazi	Maximal degradation			14.35
Atharogazi				15.73
Atharogazi				15.83
Atharogazi				15.84
Atharogazi				15.84
Lakkhipur	Pristine	*Tamarix aphylla*	Pinitol in (µg/g) of FW	865.283
Lakkhipur				926.276
Lakkhipur				927.001
Birat	Intermediate degradation 1			789.685
Birat				780.537
Birat				790.674
Island X	Intermediate degradation 2			684.007
Island X				680.060
Island X				676.880
Gopalnagar	Maximal			887.326

Brajaballavpur	degradation			950.560
Atharogazi				655.810
Durbachoti	Pristine	*Nypa fruticans*	Soluble sugar to starch ratio	3.776
Durbachoti				2.968
Durbachoti				2.137
G-Plot				3.134
G-Plot				3.528
G-Plot				3.477
Birat	Intermediate degradation 1			6.369
Birat				5.037
Dashpur1				5.538
Island X	Intermediate degradation 2			7.056
Island X				7.892
Island X				7.569
Dashpur 2				7.892
Rakkhoshkhali				7.310
Rakkhoshkhali				7.256
Lakkhipur island				7.198
Lakkhipur island				7.209
Patharprotima	Maximal degradation			9.610
Patharprotima				9.433
Gopalnagar				8.520
Gopalnagar				8.139
Gopalnagar				9.606
Brajaballavpur				8.179
Brajaballavpur				9.006
Brajaballavpur				9.148
Lothian	Pristine		Unit of SOD / mg of protein	16.358
ShiberGhat	Intermediate degradation 1			13.233
Lakkhipur island	Intermediate degradation 2			16.456
Prentice				15.867
Patharprotima	Maximal degradation			15.674
Durbachoti	Pristine	*Phoenix paludosa*	Myo-inositol in (mg/g of DW)	2.144
G-Plot				3.348
Lothian				3.270
Lakkhipur				6.357
Birat	Intermediate degradation 1			13.041
ShiberGhat				14.807
L-Plot				14.661
Dashpur1				15.350
Island X	Intermediate degradation 2			18.999
Island X				19.356
Rakkhoshkhali				16.366
Rakkhoshkhali				16.353
Dashpur 2				18.064
Dashpur 2				19.627
Brajaballavpur	Maximal degradation			22.332
Brajaballavpur				22.990
Brajaballavpur				23.898

Brajaballavpur				21.467
Ramganga				22.452
Ramganga				21.658
Ramganga				20.753
Durbachoti	Pristine		Soluble sugar to starch ratio	4.980
G-Plot				3.793
Dashpur1	Intermediate degradation 1			6.760
Dashpur1				5.432
Birat				5.152
ShiberGhat				5.190
Island X	Intermediate degradation 2			7.465
Dashpur 2				7.779
Dashpur 2				7.056
Dashpur 2				7.892
Dashpur 2				7.569
Rakkhoshkhali				7.064
Brajaballavpur	Maximal degradation			9.126
Atharogazi				9.043
Atharogazi				9.133
Atharogazi				8.153
G-Plot	Pristine		Free amino acids (mg/g of FW)	0.749
Lothian				1.099
Lothian				0.758
ShiberGhat	Intermediate degradation 1			1.785
ShiberGhat				1.419
L-Plot				1.528
Rakkhoshkhali	Intermediate degradation 2			1.824
Dashpur 2				1.751
Island X				1.783
Atharogazi	Maximal degradation			3.826
Patharprotima				3.313
Gopalnagar				3.171
Gopalnagar				4.284
G-Plot	Pristine		Unit of SOD / mg of protein	19.638
Dashpur1	Intermediate degradation 1			19.361
Rakkhoshkhali	Intermediate degradation 2			18.901
Rakkhoshkhali				19.654
Gopalnagar	Maximal degradation			20.783
G-Plot	Pristine		Total Na+/K+	0.619
L-Plot	Intermediate degradation 1			0.564
Prentice	Intermediate degradation 2			1.216
Rakkhoshkhali				1.374
Gopalnagar	Maximal degradation			1.998
Durbachoti	Pristine		Total chlorophyll (mg/100 g of FW)	149.384
L-Plot	Intermediate degradation 1			112.564
Dashpur 2	Intermediate degradation 2			105.674
Dashpur 2				90.038
Prentice				90.454
Ramganga	Maximal			90.799

Ramganga	degradation			107.480
Lakkhipur	Pristine		PEPC activity/RuBPC activity (µmol/minute/µg of protein)	0.505
G-Plot				0.502
ShiberGhat	Intermediate degradation 1			0.455
Island X	Intermediate degradation 2			0.436
Lakkhipur island				0.436
Prentice				0.434
Island X				0.436
Dashpur 2				0.435
Atharogazi	Maximal degradation			0.404
Lothian	Pristine	*Porteresia coarctata*	Proline in (mg/g of FW)	0.593
Lakkhipur				0.513
Lakkhipur				0.615
Lakkhipur				0.751
Lothian				0.391
Lothian				0.705
Birat	Intermediate degradation 1			0.913
Birat				0.926
Birat				0.954
Birat				0.948
Birat				0.960
Dashpur1				0.961
Dashpur 2	Intermediate degradation 2			1.358
Dashpur 2				1.233
Dashpur 2				1.704
Dashpur 2				1.680
Prentice				1.896
Prentice				1.947
Prentice				1.622
Island X				1.638
Island X				1.931
Island X				1.217
Atharogazi	Maximal degradation			4.386
Brajaballavpur				2.701
Brajaballavpur				3.726
Brajaballavpur				3.800
Brajaballavpur				4.593
Brajaballavpur				4.670
Ramganga				3.668
Ramganga				3.230
Ramganga				3.475
Lothian	Pristine		Soluble sugar to starch ratio	3.118
Lothian				2.995
Lothian				3.647
Dashpur1	Intermediate degradation 1			4.932
Dashpur1				4.921
Birat				4.849
ShiberGhat				4.230
ShiberGhat				4.968
Dashpur1				4.708
Island X	Intermediate			6.826

Island X	degradation 2			6.404
Island X				6.416
Rakkhoshkhali				6.429
Rakkhoshkhali				6.228
Dashpur 2				6.469
Prentice				6.465
Prentice				6.476
Gopalnagar	Maximal degradation			9.991
Gopalnagar				10.115
Brajaballavpur				9.458
Brajaballavpur				9.699
G-Plot	Pristine		Unit of SOD / mg of protein	15.269
ShiberGhat	Intermediate degradation 1			16.784
Prentice	Intermediate degradation 2			18.122
Dashpur 2				19.763
Gopalnagar	Maximal degradation			24.330
Lothian	Pristine		Total Na+/K+	1.540
ShiberGhat	Intermediate degradation 1			1.625
Prentice	Intermediate degradation 2			2.860
Island X				3.072
Ramganga	Maximal degradation			1.988
Ramganga				0.954
Lothian	Pristine		*P5CS* (ΔCQ)	3.34
Lothian				3.36
Lothian				3.4
ShiberGhat	Intermediate degradation 1			5.37
L-Plot				5.49
L-Plot				5.59
L-Plot				5.61
L-Plot				5.66
L-Plot				5.72
Dashpur 2	Intermediate degradation 2			10.97
Prentice				10.44
Island X				10.11
Island X				11.09
Island X				11.12
Island X				10.8
Rakkhoshkhali				10.38
Gopalnagar	Maximal degradation			14.83
Gopalnagar				16.66
Gopalnagar				16.81
Gopalnagar				17.06
Brajaballavpur				18.65
Ramganga				15.76
Ramganga				15.77
Ramganga				16.88
Lakkhipur	Pristine		*MIPS*(ΔCQ)	3.2
Durbachoti				3.73
Durbachoti				3.86
Durbachoti				3.97
ShiberGhat	Intermediate			7.85

ShiberGhat	degradation 1			7.89
ShiberGhat				7.9
Dashpur1				7.93
Dashpur1				7.95
Dashpur1				7.96
Rakkhoshkhali	Intermediate degradation 2			12.4
Rakkhoshkhali				11.02
Rakkhoshkhali				10.08
Dashpur 2				11.66
Dashpur2				11.62
Prentice				11.54
Prentice				11.82
Prentice				10.06
Gopalnagar	Maximal degradation			18.02
Gopalnagar				18.04
Gopalnagar				18.06
Brajaballavpur				18.67
Brajaballavpur				18.94
Brajaballavpur				18.62
Bhagabatpur	Pristine		*F16BP*(ΔCQ)	2.5
Lakkhipur				2.53
Durbachoti				2.57
Lakkhipur				2.61
Durbachoti				2.44
G-Plot				2.34
G-Plot				2.22
Birat	Intermediate degradation 1			7
ShiberGhat				7.09
L-Plot				7.11
Dashpur1				7.58
Dashpur1				7.78
ShiberGhat				7.82
Dashpur1				5.86
Dashpur1				5.98
Dashpur1				5.26
Dashpur1				7.1
Prentice	Intermediate degradation 2			9.21
Island X				9.24
Lakkhipur island				9.37
Rakkhoshkhali				8.98
Rakkhoshkhali				8.96
Rakkhoshkhali				9.34
Rakkhoshkhali				9.37
Rakkhoshkhali				9.38
Dashpur 2				9.28
Island X				10.62
Rakkhoshkhali				10.68
Rakkhoshkhali				10.26
Rakkhoshkhali				9.65
Rakkhoshkhali				10.48
Patharprotima	Maximal degradation			11.98
Patharprotima				11.98
Patharprotima				11.87

Gopalnagar				12.87
Gopalnagar				12.76
Gopalnagar				12.65
Gopalnagar				12.54
Lakkhipur	Pristine		*FBA*(ΔCQ)	3.39
Lakkhipur				4.2
Lakkhipur				4.25
Lothian				3.68
Lothian				3.84
ShiberGhat	Intermediate degradation 1			6.52
ShiberGhat				6.62
ShiberGhat				6.78
ShiberGhat				7.09
L-Plot				7.22
ShiberGhat				7.25
Prentice	Intermediate degradation 2			10.03
Island X				10.05
Lakkhipur island				10.09
Rakkhoshkhali				10.13
Dashpur 2				10.27
Island X				10.32
Island X				10.33
Island X				10.52
Island X				10.65
Atharogazi	Maximal degradation			13.64
Atharogazi				13.68
Atharogazi				13.8
Atharogazi				13.89
Atharogazi				13.95
Lothian	Pristine		*F26BP*(ΔCQ)	2.12
Lakkhipur				2.14
G-Plot				2
G-Plot				2.54
G-Plot				2.52
Birat	Intermediate degradation 1			4.42
Birat				4.44
ShiberGhat				4.28
ShiberGhat				4.28
ShiberGhat				4.98
Island X	Intermediate degradation 2			9.86
Lakkhipur island				9.88
Lakkhipur island				9.91
Lakkhipur island				9.99
Lakkhipur island				10.11
Lakkhipur island				10.11
Rakkhoshkhali				10.16
Rakkhoshkhali				10.17
Patharprotima	Maximal			15.88

Patharprotima	degradation			15.88
Patharprotima				15.91
Patharprotima				16
Patharprotima				16.02
Gopalnagar				16.07
G-Plot	Pristine	*Myriostachya wightiana*	Pinitol in (µg/g) of FW	86.838
ShiberGhat	Intermediate degradation 1			795.500
ShiberGhat				786.000
Dashpur 2	Intermediate degradation 2			694.176
Dashpur 2				683.739
Dashpur 2				684.107
Dashpur 2				680.906
Dashpur 2				676.188
Atharogazi	Maximal degradation			615.523
Atharogazi				615.522
Atharogazi				564.900
Atharogazi				625.860
G-Plot	Pristine		Unit of SOD / mg of protein	16.384
Dashpur1	Intermediate degradation 1			20.044
Rakkhoshkhali	Intermediate degradation 2			30.227
Gopalnagar	Maximal degradation			50.324
Lothian	Pristine		Total Na+/K+	2.951
ShiberGhat	Intermediate degradation 1			2.985
Dashpur1				2.799
Rakkhoshkhali	Intermediate degradation 2			3.564
Rakkhoshkhali				3.752
Dashpur 2				3.549
Patharprotima	Maximal degradation			3.979
Patharprotima				2.962
Gopalnagar				2.853
Lakkhipur	Pristine		*F16BP*(ΔCQ)	5.01
Lakkhipur				5.21
Lakkhipur				5.28
G-Plot				5.24
G-Plot				5.26
G-Plot				5.14
Lothian				5.88
ShiberGhat	Intermediate degradation 1			8.05
L-Plot				8.06
L-Plot				8.18
L-Plot				8.47
L-Plot				8.53
L-Plot				8.64
Birat				8.16
Birat				8.18
Birat				8.11
Prentice	Intermediate degradation 2			11.32
Prentice				11.36
Prentice				11.54
Prentice				11.51

Prentice				11.24
Island X				11.48
Island X				11.42
Island X				11.49
Island X				10.64
Dashpur 2				11.62
Dashpur 2				11.98
Dashpur 2				11.63
Rakkhoshkhali				10.48
Dashpur 2				11.82
Gopalnagar	Maximal degradation			13.98
Brajaballavpur				13.87
Brajaballavpur				13.76
Brajaballavpur				13.65
Brajaballavpur				13.54
Ramganga				13.54
Ramganga				13.12
Ramganga				13.16
Ramganga				13.28
Durbachoti	Pristine	*Sporobolus virginicus*	Proline in (mg/g of FW)	0.332
Lothian				0.348
Lothian				0.593
Dashpur1	Intermediate degradation 1			0.937
Dashpur1				0.943
Dashpur1				0.906
Dashpur1				0.759
Rakkhoshkhali	Intermediate degradation 2			1.744
Rakkhoshkhali				1.457
Rakkhoshkhali				1.880
Dashpur 2				1.714
Dashpur 2				1.238
Dashpur 2				1.026
Dashpur 2				1.030
Prentice				1.034
Prentice				1.047
Prentice				1.029
Patharprotima	Maximal degradation			3.634
Patharprotima				3.578
Patharprotima				3.606
Patharprotima				3.634
Patharprotima				3.606
Gopalnagar				3.549
Gopalnagar				2.383
Gopalnagar				2.415
Gopalnagar				2.415
Gopalnagar				2.446
Lothian	Pristine		Free amino acids (mg/g of FW)	0.769
Lothian				1.175
G-Plot				0.745
ShiberGhat	Intermediate degradation 1			2.856
L-Plot				2.103
L-Plot				2.146
Prentice	Intermediate			1.996

Island X	degradation 2			3.798
Rakkhoshkhali				3.790
Brajaballavpur	Maximal degradation			6.416
Brajaballavpur				5.900
Ramganga				6.099
Lothian	Pristine		Unit of SOD / mg of protein	25.280
ShiberGhat	Intermediate degradation 1			24.399
Lakkhipur island	Intermediate degradation 2			31.632
Lakkhipur island				30.674
Gopalnagar	Maximal degradation			32.137
Brajaballavpur				30.472
G-Plot	Pristine		*P5CS* (ΔCQ)	2.36
Durbachoti				2.32
Durbachoti				2.37
Durbachoti				2.5
Lothian				2.456
ShiberGhat	Intermediate degradation 1			5.97
ShiberGhat				5.28
ShiberGhat				5.55
Dashpur1				5.23
Dashpur1				5.789
Dashpur1				5.344
Lakkhipur island	Intermediate degradation 2			10.02
Rakkhoshkhali				10.12
Rakkhoshkhali				10.21
Rakkhoshkhali				10.21
Rakkhoshkhali				10.28
Rakkhoshkhali				10.46
Brajaballavpur	Maximal degradation			13.62
Ramganga				14.67
Ramganga				14.87
Ramganga				15.67
Durbachoti	Pristine		*FBA*(ΔCQ)	4.58
Durbachoti				4.6
Durbachoti				5.23
Durbachoti				5.3
Durbachoti				5.36
L-Plot	Intermediate degradation 1			8.11
Dashpur1				8.13
Dashpur1				8.32
Dashpur1				8.96
Dashpur1				8.98
Rakkhoshkhali	Intermediate degradation 2			10.58
Rakkhoshkhali				10.46
Dashpur 2				10.3
Dashpur 2				10.42
Dashpur 2				11.94
Dashpur 2				11.82
Dashpur2				10.62
Prentice				10.22

Prentice				9.12
Prentice				10.16
Ramganga	Maximal degradation			13.68
Ramganga				14.32
Ramganga				15.82
Ramganga				15.64
Gopalnagar				15.88
G-Plot	Pristine		*F26BP*(ΔCQ)	2.56
G-Plot				2.68
Lothian				2.88
Lothian				3.1
L-Plot	Intermediate degradation 1			5.92
L-Plot				5.96
L-Plot				5.98
Dashpur1				6.18
Dashpur1				6.22
Dashpur1				6.19
Rakkhoshkhali	Intermediate degradation 2			10.24
Rakkhoshkhali				10.3
Rakkhoshkhali				10.35
Dashpur 2				10.35
Dashpur 2				10.37
Dashpur 2				10.41
Dashpur 2				12.21
Prentice				12.82
Prentice				12.85
Island X				10.24
Island X				10.14
Island X				10.54
Island X				7.92
Gopalnagar	Maximal degradation			16.07
Gopalnagar				16.11
Gopalnagar				16.58
Gopalnagar				16.66
Brajaballavpur				16.72
Brajaballavpur				16.76
Brajaballavpur				16.85
Brajaballavpur				16.92
Brajaballavpur				16.93
G-Plot	Pristine		*SUS*(ΔCQ)	4.04
G-Plot				4.07
Lothian				4.13
Lothian				4.16
Lothian				4.17
Lothian				4.2
Lothian				4.22
Birat	Intermediate degradation 1			7.37
Birat				7.39
Birat				7.39
Birat				7.48
Birat				7.5
ShiberGhat				7.55
ShiberGhat				7.59

ShiberGhat				7.64
Dashpur1				7.64
Dashpur1				7.64
Dashpur1				7.69
Prentice	Intermediate degradation 2			11.22
Prentice				11.43
Prentice				11.78
Prentice				10.46
Island X				10.34
Rakkhoshkhali				11.22
Rakkhoshkhali				10.28
Rakkhoshkhali				10.26
Rakkhoshkhali				10.62
Dashpur 2				10.24
Dashpur 2				10.26
Dashpur 2				11.24
Brajaballavpur	Maximal degradation			14.4
Brajaballavpur				13.72
Brajaballavpur				13.74
Ramganga				14.89
Ramganga				14.99
Ramganga				15.09
Ramganga				15.34
Ramganga				15.4
Ramganga				15.53
Lothian	Pristine	*Paspalum vaginatum*	Free amino acids (mg/g of FW)	1.087
Lakkhipur				0.970
Durbachoti				0.983
Dashpur1	Intermediate degradation 1			2.159
Dashpur1				2.058
Dashpur1				2.661
Island X	Intermediate degradation 2			2.476
Dashpur 2				3.072
Dashpur 2				3.079
Lakkhipur island				3.813
Patharprotima	Maximal degradation			5.922
Patharprotima				7.266
Gopalnagar				8.792
Durbachoti	Pristine		Unit of SOD / mg of protein	19.367
L-Plot	Intermediate degradation 1			25.963
Lakkhipur island	Intermediate degradation 2			28.557
Lakkhipur island				25.119
Brajaballavpur	Maximal degradation			35.486
Brajaballavpur				28.149
Lothian	Pristine		*P5CS* (ΔCQ)	2.15
Lakkhipur				2.06
G-Plot				2
G-Plot				2.22
Lothian				2.14
ShiberGhat	Intermediate			4.41

ShiberGhat	degradation 1			4.5
Dashpur1				4.987
Dashpur1				4.899
Dashpur1				4.78
Dashpur1				4.87
Birat				5.655
Dashpur2	Intermediate degradation 2			10.76
Prentice				10.07
Prentice				8.61
Prentice				9.27
Rakkhoshkhali				9.22
Lakkhipur island				9.92
Lakkhipur island				9.99
Atharogazi	Maximal degradation			12.06
Atharogazi				12.18
Patharprotima				12.34
Patharprotima				12.61
Patharprotima				13.95
Patharprotima				13.96
Patharprotima				14.02
Gopalnagar				14.58
Lothian	Pristine		*MIPS*(ΔCQ)	2.82
Lothian				3.17
Lothian				2.31
G-Plot				3.11
G-Plot				3.21
Birat	Intermediate degradation 1			6.31
Birat				6.31
Birat				6.51
Birat				7.72
Birat				7.84
Lakkhipur island	Intermediate degradation 2			13.58
Rakkhoshkhali				13.74
Rakkhoshkhali				11.24
Rakkhoshkhali				11.81
Rakkhoshkhali				12.43
Rakkhoshkhali				8.14
Dashpur 2				9.22
Dashpur 2				8.51
Dashpur 2				11.78
Brajaballavpur	Maximal degradation			18.02
Ramganga				18.62
Ramganga				17.09
Ramganga				17.53
Ramganga				17.59
Durbachoti	Pristine		*F16BP*(ΔCQ)	4.86
Durbachoti				4.88
Durbachoti				4.12
Durbachoti				4.28
Lothian				4.28
Lothian				4.62

Dashpur1	Intermediate degradation 1			7.12
Birat				7.04
Birat				7.18
Dashpur1				7.62
Dashpur1				7.14
Rakkhoshkhali	Intermediate degradation 2			10.59
Dashpur 2				10.83
Island X				10.96
Island X				10.98
Dashpur 2				10.36
Dashpur 2				10.22
Dashpur 2				10.44
Dashpur 2				10.27
Dashpur2				10.59
Prentice				10.49
Prentice				10.46
Prentice				10.34
Ramganga	Maximal degradation			12.74
Atharogazi				12.85
Patharprotima				12.9
Atharogazi				12.78
Patharprotima				12.94
Brajaballavpur				12.87
Ramganga				12.43
Ramganga				12.65
Lothian	Pristine	*Finlaysonia obovata*	Myo-inositol in (mg/g of DW)	6.927
Lakkhipur				7.374
Durbachoti				7.368
Durbachoti				5.785
Durbachoti				8.368
ShiberGhat	Intermediate degradation 1			13.485
ShiberGhat				15.643
Dashpur1				11.968
Dashpur1				15.047
Dashpur1				14.350
Island X	Intermediate degradation 2			24.493
Island X				21.005
Island X				17.460
Island X				18.498
Lakkhipur island				24.875
Lakkhipur island				24.916
Lakkhipur island				25.001
Lakkhipur island				21.864
Lakkhipur island				17.010
Prentice				24.493
Atharogazi	Maximal degradation			57.271
Atharogazi				57.136
Atharogazi				57.091
Lothian	Pristine		Unit of SOD / mg of protein	39.014

ShiberGhat	Intermediate degradation 1			30.686
Rakkhoshkhali	Intermediate degradation 2			25.756
Dashpur 2				20.564
Gopalnagar	Maximal degradation			25.164
Durbachoti	Pristine		Total Na+/K+	2.134
Dashpur1	Intermediate degradation 1			1.234
Prentice	Intermediate degradation 2			0.975
Island X				0.700
Ramganga	Maximal degradation			0.907
Lothian	Pristine		Total chlorophyll (mg/100 g of FW)	53.022
Dashpur1	Intermediate degradation 1			67.342
Dashpur1				55.378
Island X	Intermediate degradation 2			33.898
Island X				32.850
Brajaballavpur	Maximal degradation			25.232
Durbachoti	Pristine		Leaf thickness (LT) µm	38.480
Durbachoti				22.400
G-Plot				21.360
G-Plot				36.850
G-Plot				23.480
Dashpur1	Intermediate degradation 1			132.250
Birat				139.880
Dashpur1				148.650
Dashpur 2	Intermediate degradation 2			195.850
Dashpur 2				187.350
Dashpur 2				189.250
Brajaballavpur	Maximal degradation			215.560
Brajaballavpur				241.020
Ramganga				221.470
Ramganga				220.680
Durbachoti	Pristine		Water storage tissue (WST) µm	1.470
Durbachoti				8.292
G-Plot				5.298
G-Plot				0.850
G-Plot				3.141
Dashpur1	Intermediate degradation 1			68.820
Birat				65.720
Dashpur1				92.020
Dashpur 2	Intermediate degradation 2			137.140
Dashpur 2				131.520
Dashpur 2				131.490
Brajaballavpur	Maximal degradation			165.300
Brajaballavpur				170.870
Ramganga				162.220
Ramganga				169.000
Durbachoti	Pristine		Palisade tissue (PT) µm	18.450
Durbachoti				7.180
G-Plot				8.780
G-Plot				14.320

G-Plot				10.570
Dashpur1	Intermediate degradation 1			21.450
Birat				44.690
Dashpur1				54.630
Dashpur 2	Intermediate degradation 2			50.650
Dashpur 2				42.350
Dashpur 2				46.780
Brajaballavpur	Maximal degradation			38.260
Brajaballavpur				53.150
Ramganga				52.250
Ramganga				49.680
Durbachoti	Pristine		Spongy tissue (ST) µm	17.560
Durbachoti				6.300
G-Plot				6.600
G-Plot				21.680
G-Plot				9.500
Dashpur1	Intermediate degradation 1			40.980
Birat				28.470
Dashpur1				1.100
Dashpur 2	Intermediate degradation 2			7.060
Dashpur 2				7.480
Dashpur 2				7.980
Brajaballavpur	Maximal degradation			12.000
Brajaballavpur				16.000
Ramganga				6.000
Ramganga				2.000
Durbachoti	Pristine		PT/ST	1.051
Durbachoti				1.140
G-Plot				1.330
G-Plot				0.661
G-Plot				1.113
Dashpur1	Intermediate degradation 1			0.523
Birat				1.570
Dashpur1				49.664
Dashpur 2	Intermediate degradation 2			7.174
Dashpur 2				5.662
Dashpur 2				5.862
Brajaballavpur	Maximal degradation			3.188
Brajaballavpur				3.322
Ramganga				8.708
Ramganga				24.840
Durbachoti	Pristine		PT/LT	0.479
Durbachoti				0.321
G-Plot				0.411
G-Plot				0.389
G-Plot				0.450
Dashpur1	Intermediate degradation 1			0.162
Birat				0.319
Dashpur1				0.368
Dashpur 2	Intermediate degradation 2			0.259
Dashpur 2				0.226
Dashpur 2				0.247
Brajaballavpur	Maximal			0.177

Brajaballavpur	degradation			0.221
Ramganga				0.236
Ramganga				0.225
Durbachoti	Pristine		ST/LT	0.456
Durbachoti				0.281
G-Plot				0.309
G-Plot				0.588
G-Plot				0.405
Dashpur1	Intermediate degradation 1			0.310
Birat				0.204
Dashpur1				0.007
Dashpur 2	Intermediate degradation 2			0.036
Dashpur 2				0.040
Dashpur 2				0.042
Brajaballavpur	Maximal degradation			0.056
Brajaballavpur				0.066
Ramganga				0.027
Ramganga				0.009
Durbachoti	Pristine		WST/LT	0.038
Durbachoti				0.370
G-Plot				0.248
G-Plot				0.023
G-Plot				0.134
Dashpur1	Intermediate degradation 1			0.520
Birat				0.470
Dashpur1				0.619
Dashpur 2	Intermediate degradation 2			0.700
Dashpur 2				0.702
Dashpur 2				0.695
Brajaballavpur	Maximal degradation			0.767
Brajaballavpur				0.709
Ramganga				0.732
Ramganga				0.766
Bhagabatpur	Pristine		*FBA*(ΔCQ)	3.12
Lakkhipur				3.14
Durbachoti				3.21
G-Plot				3.22
Lothian				3.34
Birat	Intermediate degradation 1			6.02
ShiberGhat				6.13
L-Plot				6.13
Dashpur1				6.15
Dashpur1				6.16
ShiberGhat				6.32
Rakkhoshkhali	Intermediate degradation 2			9.58
Dashpur 2				9.68
Dashpur 2				9.85
Dashpur 2				10
Dashpur 2				10.56
Prentice				9.48
Island X				9.16
Island X				9.18
Island X				9.1

Island X				11.48
Ramganga	Maximal degradation			13.21
Atharogazi				13.26
Patharprotima				13.31
Gopalnagar				13.34
Brajaballavpur				13.45
G-Plot	Pristine	*Acronychia pedunculata*	Total Na+/K+	0.489
L-Plot	Intermediate degradation 1			0.579
Dashpur1				0.439
Birat				0.676
Island X	Intermediate degradation 2			0.570
Dashpur 2				0.695
Dashpur 2				0.700
Brajaballavpur	Maximal degradation			0.459
Lothian	Pristine		Total chlorophyll (mg/100 g of FW)	75.453
Durbachoti				67.546
ShiberGhat	Intermediate degradation 1			77.784
ShiberGhat				75.438
Dashpur1				75.767
Prentice	Intermediate degradation 2			76.458
Island X				78.653
Prentice				80.283
Prentice				66.385
Ramganga	Maximal degradation			77.574
